# Community acceptance of environmental larviciding against malaria with *Bacillus thuringiensis israelensis* in rural Burkina Faso – A knowledge, attitudes and practices study

**DOI:** 10.1080/16549716.2021.1988279

**Published:** 2021-12-20

**Authors:** Peter Dambach, Issouf Traoré, Hélène Sawadogo, Pascal Zabré, Sharvari Shukla, Rainer Sauerborn, Norbert Becker, Revati Phalkey

**Affiliations:** a Heidelberg Institute of Global Health, University of Heidelberg, Heidelberg, Germany; b Centre De Recherche En Santé De Nouna, Nouna, Burkina Faso; c Institut De Formations Initiale Et Continue, Université Thomas Sankara, Ouagadougou, Burkina Faso; d Symbiosis Statistical Institute, Symbiosis International (Deemed University); e German Mosquito Control Association (KABS), Speyer, Germany; f Centre for Organismal Studies, University of Heidelberg, Heidelberg, Germany; g Epidemiology and Public Health Division, University of Nottingham, Nottingham, UK; h Climate Change and Health Group, Public Health England, Chilton, UK

**Keywords:** Malaria, vector control, larval source management, community acceptability, Burkina fasos

## Abstract

**Background:**

Malaria control is based on early treatment of cases and on vector control. The current measures for malaria vector control in Africa are mainly based on long-lasting insecticidal nets (LLINs) and to a much smaller extent on indoor residual spraying (IRS). While bed net use is widely distributed and its role is intensively researched, Bti-based larviciding is a relatively novel tool in Africa. In this study, we analyze the perception and acceptability of Bti-based larval source management under different larviciding scenarios that were performed in a health district in Burkina Faso.

**Objective:**

To research people’s perception and acceptance regarding biological larviciding interventions against malaria in their communities.

**Methods:**

A cross-sectional study was undertaken using a total of 634 administered questionnaires. Data were collected in a total of 36 rural villages and in seven town quarters of the semi-urban town of Nouna.

**Results:**

Respondents had basic to good knowledge regarding malaria transmission and how to protect oneself against it. More than 90% reported sleeping under a bed net, while other measures such as mosquito coils and insecticides were only used by a minority. The majority of community members reported high perceived reductions in mosquito abundance and the number of malaria episodes. There was a high willingness to contribute financially to larviciding interventions among interviewees.

**Conclusions:**

This study showed that biological larviciding interventions are welcomed by the population that they are regarded as an effective and safe means to reduce mosquito abundance and malaria transmission. A routine implementation would, despite low intervention costs, require community ownership and contribution.

## Background

**Figure 1.**
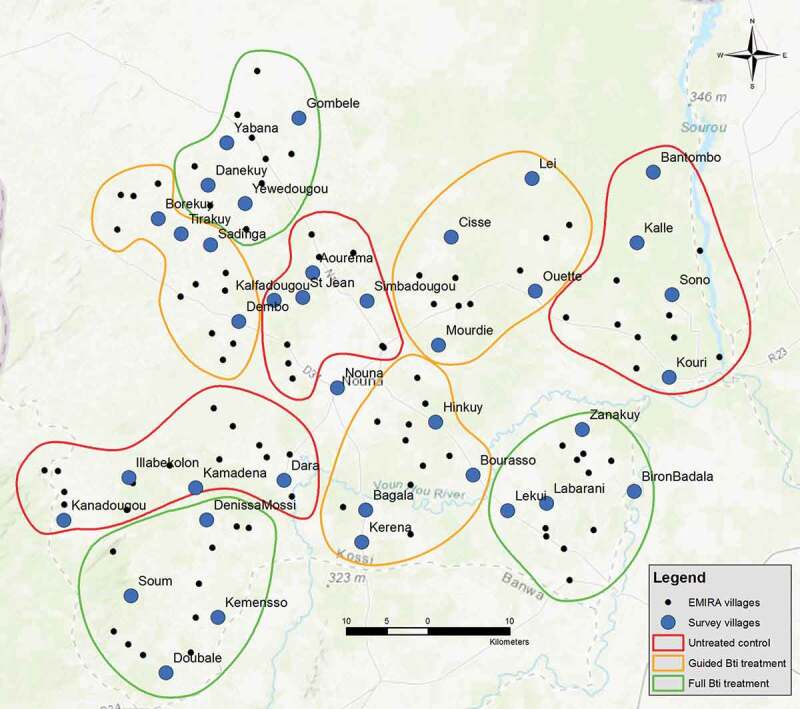
Map of the study region showing intervention clusters and villages in which questionnaires were administered. In each village represented by a blue dot, 15 questionnaire interviews were performed

**Figure 2.**
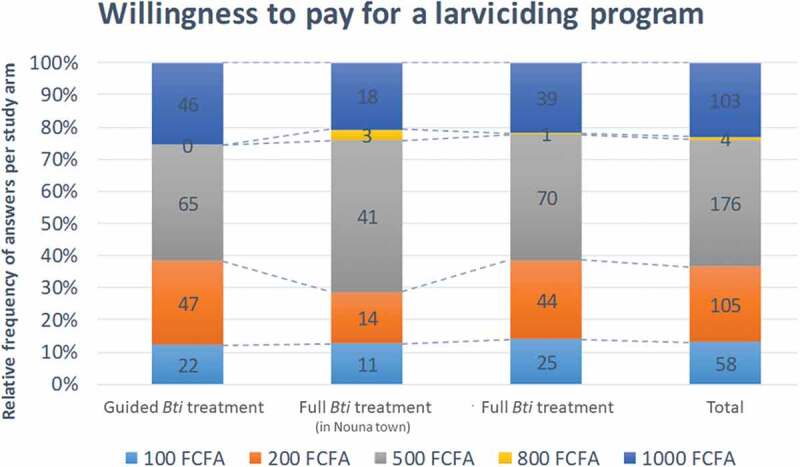
Willingness to pay for a larviciding program within the greater nouna region

Despite recent efforts, malaria is still a major public health challenge, particularly in sub-Saharan Africa. In 2019, there were an estimated 409,000 malaria deaths globally, of which approximately 95% occurred in Africa [1]. The highest prevalence of cases and, in particular, deaths occur among children under the age of five (90%) representing the leading cause of child mortality in Africa. Almost half a century after the ceded goal of malaria eradication via DDT use was set; larviciding has become an important component in the control of a multitude of vector borne infectious diseases. In its current deployment, however, the types of larvicides used shifted away from chemical insecticides that can be harmful to the environment, towards ecologically sound toxins based on *Bacillus thuringiensis israelensis (Bti)* and *Bacillus sphaericus (Bs)*, which selectively kill Culicidae larvae and cause no harm to flora and fauna. For several reasons microbial larviciding is an attractive malaria control tool. In contrast to most chemical larvicides, Bti has a low resistance potential and virtually no losses in efficacy have been observed [2–5]. Depending on the environment, the local climate and the distribution of stagnant surface water, larviciding with Bti can be an important additional tool for vector population control. In many environmental settings, larvae are found at high densities in their respective breeding sites and can be easily accessed, therefore adult vector populations can be reduced by orders of magnitude with this Larval Source Management (LSM) [6–10]. Combined with long-lasting impregnated nets (LLINs) a twofold reduction in new malaria infections was achieved compared to LLINs alone [11]. Further studies showed that LSM as an additional component in vector control programs led to a reduction of malaria of more than 50% [12]. Cost estimations of several studies in Africa indicate that larviciding is not only cost-effective but also cost-competitive with other malaria control strategies [7,9,13]. If supported by the community, LSM has the potential to be a promising and sustainable method [14]Figure 1, Figure 2.

In rural areas, where health and environmental intervention campaigns often rely on the involvement of the local population, people’s understanding and acceptance can be a crucial factor for a campaign’s success. The more malaria control approaches move away from centrally managed ‘one size fits all’ approaches towards locally conducted and managed programs, the more their success relies on the cooperation and understanding of each affected person [15]. Furthermore, it is important to gather information on people’s knowledge of, their attitude towards and the perceived success of a larviciding program. Currently, there are only few data available on communities’ general perception on larviciding programs [16,17], while their perceived successfulness in terms of vector and malaria reduction has been left uninvestigated.

## Objective

The objective of this study was to evaluate the population’s perception on *Bti* based larviciding for malaria control and to assess the community acceptance within the Nouna health district. Furthermore, we wanted to collect evidence on people’s trust and confidence in the effectiveness of such a program and their willingness of getting involved and to contribute financially or with labor. Here, the quantitative results collected with guided questionnaires are presented. Qualitative results from in-depth interviews and focus group discussions have been published elsewhere [18].

## Methods

### Study design and participants

The research was conducted within the Nouna health district, situated in the Kossi province in North-Western Burkina Faso. Except for the semi-urban district capital of Nouna, all surrounding villages have rural characteristics and their number of inhabitants ranges from several hundred to a few thousand. People’s access to basic healthcare is available through a total of 47 rural health centers (CSPS). The Sudano-Sahelian climate features annual precipitations of around 800 mm, which fall during the rainy season between June and September. The period of highest rainfalls equally marks the time of highest abundance of malaria vector mosquitoes, leading to high transmission until October. Malaria is holo-endemic within the region, and entomological inoculation rates (EIR) and the number of infective bites per person per year have been reported to be as high as several hundred [19,20]. During the dry season between November and April malaria transmission is low. The main vectors for malaria are *Anopheles gambiae* sl. with more than 98% and to a much smaller extent *A. funestus* and *A. nili*. Malaria transmission occurs throughout the year, with a seasonal peak during the late rainy season in August [21]. Within the study region Insecticide Treated Nets (ITNs) are used, and intermittent preventive treatment in pregnancy (IPTp), and early diagnosis and treatment of malaria cases are performed [22]. Indoor residual spraying (IRS) is virtually absent.

In the study area there exists a long-standing collaboration between rural communities and the research center, which allows for constant and detailed exchange of information on both sides. For the purpose of this study, on-site visits of a communications officer and regular radio broadcasts were used to increase project visibility and to inform about project goals and basic study procedures (e.g. visiting of spray teams for malaria control and biological nature of the used larvicides).

The research project EMIRA (Ecologic Malaria Reduction for Africa) [23] of which this study is a part was granted ethical clearance from the national ethics committee of Burkina Faso, the local ethics committee in Nouna, Kossi and the ethics board of the Heidelberg University, Heidelberg, Germany. Informed consent was obtained from survey participants. Confidentiality and privacy of participants was maintained throughout the study, and study participants were assured of their rights to withdraw from the questionnaire-based interview at any time.

## Data collection

This evaluation took place towards the end of the second year of a two-year larviciding intervention within the Nouna health district [23,24]. The 127 rural study villages were distributed into clusters. One-third received spraying with the biological larvicide Bacillus thuringiensis israelensis (Bti) in all breeding sites within and around villages; one-third received selective treatment of the breeding sites with the assumed highest vector larvae productivity [25] and one-third served as an untreated control group. The district capital Nouna received full treatment.

Data collection was performed within two weeks’ time and was accomplished before the last larviciding cycle ended, to assure minimal bias in people’s retrospection. Survey questionnaires were designed for this study and piloted in Nouna and in three of the rural study villages and then used for a total of 36 villages and five of the seven town quarters of Nouna. The same villages, in which adult mosquitoes were collected in light traps [6], were used for the administration of questionnaires to allow for comparison of perceived and entomologically monitored reduction in mosquito abundance. Results of the achieved mosquito reductions in this trial are published elsewhere [6,26,27]. In each of the villages, 15 households were visited, and the family head asked for permission to conduct the questionnaire interviews. A total of 634 questionnaires were filled out. Respondents in the untreated control arm were not presented with the questionnaire section on larviciding success. The survey questions were asked orally by experienced investigators who work with the local research center. Questions were asked either in French, the local trade language Dioula, or if needed, in one of the other local languages by a native speaker. The questionnaires collected information on the knowledge, attitudes and practices of community members regarding malaria, including transmission, risk factors related to malaria, and larviciding.

## Data Analysis

Data were entered into an Access database, undergoing quality control via cross-checking of a second data entry clerk.

We presented the data of household characteristics, participants knowledge, attitude and practices regarding malaria and its vector by treatment arms. Data are represented by frequencies and percentages. For statistical analysis, differences in various households and other characteristics between treatment groups were tested by Chi-square test, incorporating Yate’s correction as applicable. A significance level of less than 0.05 is considered significant. Analyses were carried out using STATA version 17.0 software.

## Results

### Socio-demographic characteristic of households

A total of 634 heads of households were interviewed, with a share of 98% male (Table 1). The mean number of persons living in one household was 11 (SD ±7). On average, there lived 2 (SD ± 2) children under five in each of the visited households. The majority of households owned two or more mobile phones (63%), a radio (62.7%), two or more bicycles (63.8%), and a motorbike (37%). Running water from the local water provider was only available for a small proportion of households living in the semi-urban city of Nouna. Except for Nouna, where electricity is available from the grid, a high number of households had solar panels at their disposal (55.5%). Around 90% of households had two or more mosquito nets available.

**Table 1.** Household characteristics

		Treatment arms (%)					
		Untreated control	Guided treatment	Full treatment semi-urban	Full treatment	Total	p-value
	**n = 634**	**183**	**180**	**91**	**180**	**634**	
**Sex**	**Sex**						
	**Female**	25 (13.7)	25 (13.9)	34 (37.4)	18 (10.0)	102 (16.1)	**0.0001**
	**Male**	158 (86.3)	155 (86.1)	57 (62.6)	162 (90.0)	532 (83.9)	
**Education**	**Educational level**						
	No formal education	149 (82.3)	117 (65.0)	49 (53.8)	139 (77.2)	454 (71.8)	**0.0001**
	Primary school	29 (16.0)	53 (29.4)	28 (30.8)	38 (21.1)	148 (23.4)	
	Secondary school	3 (1.7)	10 (5.6)	14 (15.4)	3 (1.7)	30 (4.7)	
	University	0 (0)	0 (0)	0 (0)	0 (0)	0 (0)	
**No U5 children in household**	**U5 children**						
	0	22 (12.0)	26 (14.4)	20 (22.0)	39 (21.8)	107 (16.9)	0.0009
	1	41 (22.4)	53 (29.4)	31 (34.1)	45 (25.1)	170 (26.9)	
	2	48 (26.2)	61 (33.9)	24 (26.4)	38 (21.2)	171 (27.0)	
	3	32 (17.5)	21 (11.7)	11 (12.1)	29 (16.2)	93 (14.7)	
	>3	40 (22.3)	19 (10.6)	5 (5.3)	28 (15.7)	92 (14.5))	
**Vehicles**	**Bicycle**						
	Zero	4 (2.2)	3 (1.7)	–	11 (6.1)	18 (2.8)	0.0001
	One	56(30.6)	78 (43.3)	19 (20.9)	58 (32.2)	211 (33.3)	
	Two or more	123 (67.2)	99 (55.0)	72 (79.1)	111 (61.7)	405 (63.9)	
	**Motorbike**						
	Zero	80 (43.7)	84 (46.7)	36 (39.6)	68 (37.8)	288 (42.3)	0.019
	One	69 (37.7)	82 (45.6)	39 (42.9)	91 (50.6)	281 (44.3)	
	Two or more	34 (18.6)	14 (7.8)	16 (17.6)	21 (11.7)	85 (13.4)	
**Communication**	**Mobile phone**						
	Zero	21 (11.7)	28 (15.6)	1 (1.1)	20 (11.1)	70 (11.0)	0.0001
	One	58 (31.7)	71 (39.4)	17 (18.7)	63 (35.0)	209 (33.0)	
	Two or more	104 (56.8)	81 (45.0)	73 (80.2)	97 (53.9)	355 (56.0)	
	**Television**						
	Zero	131 (71.6)	143 (79.4)	51 (56.0)	152 (84.4)	477 (75.2)	0.0001
	One	42 (23.0)	34 (18.9)	31 (34.1)	27 (15.0)	134 (21.1)	
	Two or more	10 (5.5)	3 (1.7)	9 (9.9)	1 (0.6)	23 (3.6)	
	**Radio**	121(66.1)	104(58.1)	63(69.2)	109(60.6)	397 (62.7)	0.205
**Infrastructure**	**Used rooms**	159(86.9)	134(74.4)	89(97.8)	165(91.7)	547 (86.3)	0.005
	**Water connection**	NA	NA	8(8.7)	NA	8 (8.7)	–
	**Electricity**	0(0.0)	3(1.7)	35(38.5)	0(0.0)	38 (6.0)	
	**Solar panel**	123(67.2)	102(57.0)	20(22.0)	106(58.9)	351 (55.5)	0.0001
	**Mosquito nets per HH**						
	Zero	4 (2.2)	7 (3.9)	1 (1.1)	12 (6.7)	24 (3.8)	0.124
	One	18 (9.8)	13 (7.2)	9 (10.0)	22 (12.2)	62 (9.8)	
	Two or more	161 (88.0)	160 (88.9)	80 (88.9)	146 (81.1)	547 (86.4)	
**Livestock**	**Poultry**						
	No poultry	9 (4.9)	19 (10.6)	10 (11.0)	5 (2.8)	43 (6.8)	0.0001
	≤20	137 (74.9)	137 (76.1)	76 (83.5)	132 (73.3)	482 (76.0)	
	>20	37 (20.2)	24 (13.3)	5 (5.5)	43 (23.9)	109 (17.2)	
	**Sheep**						
	No	91 (49.7)	105 (58.3)	45 (49.5)	90 (50.0)	331 (52.2)	0.421
	One	11 (6.0)	13 (7.2)	4 (4.4)	10 (5.6)	38 (6.0)	
	Two or more	81 (44.3)	62 (34.4)	42 (46.2)	80 (44.4)	265 (41.8)	
	**Goats**						
	No	58 (31.7)	58 (32.2)	77 (84.6)	52 (28.9)	245 (38.6)	0.0001
	One	10 (5.5)	21 (11.7)	1 (1.1)	12 (6.7)	44 (6.9)	
	Two or more	115 (62.8)	101 (56.1)	13 (14.3)	116 (64.4)	345 (54.4)	
	**Cattle**						
	Nil	62 (33.9)	56 (31.1)	46 (50.5)	50 (27.8)	214 (33.8)	0.008
	One	10 (5.5)	16 (8.9)	5 (5.5)	10 (5.6)	41 (6.5)	
	Two or more	111 (60.7)	108 (60.0)	40 (44.0)	120 (66.7)	379 (59.8)	
	**Horses**	6(3.3)	5(2.8)	0(0.0)	14(7.8)	25 (3.9)	0.001
	**Donkeys**	138(75.4)	147(81.7)	51(56.0)	128(71.1)	464 (73.2)	0.0001
**Agriculture**	**Land parcels**	37(20.2)	35(19.4)	57(62.6)	42(23.3)	171 (27.0)	0.0001
	**Arable land**	183(100.0)	179(99.4)	82(90.1)	178(98.9)	622 (98.1)	0.0001

Note: Values are n(%). p-value by using Chi-square test.

## Understanding of malaria transmission

A majority of participants demonstrated basic knowledge of malaria and ways to protect themselves against it (Table 2, Table 3, Table 4). Despite correctly identifying most of the environmental risk factors for vector abundance and malaria transmission, there are some misconceptions prevailing. In general, the understanding that malaria is transmitted by infected mosquito bites was high (94%). However, less than half of the participants (46%) were aware that different stages of mosquito larvae exist. Although a high proportion (85.9%) associated stagnant water with mosquito abundance, this understanding was higher among rural participants (90%) compared to their urban counterparts (68%). Overall, less than half (40%) made the link between rainfall patterns and disease. Depending on the study arm, between 5% and 13% of respondents perceived food as a possible mode of malaria transmission, and about 22.3% identified other sources.

**Table 2.** Knowledge of malaria and its vectors

	Treatment arms (%)					
	Untreated control	Guided treatment	Full treatment semi-urban	Full treatment	Total	p-value
**n = 634**	**183**	**180**	**91**	**180**	**634**	
**How is malaria transmitted?**						
Mosquito bites	165(90.2)	173(97.2)	86(94.5)	170(94.4)	594 (94.0)	0.004
Food	24(13.1)	10(5.6)	12(13.2)	9(5.0)	55 (8.7)	0.007
other sources	52(28.4)	26(14.6)	20(22.0)	43(23.9)	141 (22.3)	0.017
Don’t Know	4(2.2)	1(0.6)	0(0.0)	2(1.1)	7 (1.1)	–
**How do mosquitos emerge?**						
Directly from egg	48(26.4)	75(42.6)	20(22.2)	65(36.9)	208 (33.3)	0.001
They go through different stages	88(48.4)	81(46.0)	43(47.8)	75(42.6)	287 (46)	0.721
Other	5(2.7)	0(0.0)	1(1.1)	2(2.2)	8 (1.3)	–
Don’t know	44(24.2)	22(12.5)	28(31.1)	36(20.5)	130 (20.8)	0.002
**What do you think are factors that favor mosquito development?**						
Rainfall	89(48.6)	44(24.7)	48(52.7)	72(40.2)	253 (40.1)	0.0001
Stagnant water	159(86.9)	168(94.4)	62(68.1)	153(85.5)	542 (85.9)	0.0001
Cleanliness of village	9(4.9)	10(5.6)	8(8.8)	5(2.8)	32 (5.1)	0.206
Humidity	27(14.8)	24(13.5)	24(26.4)	35(19.6)	110 (17.4)	0.045
**Where do you think one can find mosquito larvae?**						
Stagnant water	167(91.3)	174(96.7)	89(97.8)	174(97.2)	604 (95.4)	0.024
Forest	4(2.2)	3(1.7)	1(1.1)	1(0.6)	9 (1.4)	–
Pit latrines	102(55.7)	98(54.4)	79(86.8)	85(47.5)	364 (57.5)	0.0001
In vegetation around house	33(18.0)	24(13.3)	9(9.9)	20(11.2)	86 (13.6)	0.176
Don’t know	10 (5.5)	3 (1.7)	1 (1.1)	3 (1.7)	604 (95.4)	–
**What can one do to avoid contracting malaria?**						
Sleep under a bed net	153(83.6)	168(93.9)	87(95.6)	162(90.0)	570 (90)	0.002
Take medication	18(9.8)	6(3.4)	16(17.6)	4(2.2)	44 (7)	0.0001
Sanitize living conditions	118(64.5)	105(58.7)	70(76.9)	113(62.8)	406 (64.1)	0.025
Spray chemicals	32(17.5)	30(16.8)	19(20.9)	28(15.6)	109 (17.2)	0.751
Apply repellents on skin or clothes	11(6.0)	4(2.2)	10(11.0)	12(6.7)	37 (5.8)	0.026
Use mosquito coils	22(12.0)	29(16.2)	14(15.4)	23(12.8)	88 (13.9)	0.642
Nothing	1(0.5)	0(0.0)	1(1.1)	1(0.6)	3 (0.5)	–
Other	33(18.0)	15(8.4)	9(9.9)	20(11.1)	77 (12.2)	0.036
Don’t Know	1(0.5)	1(0.6)	0(0.0)	0(0.0)	2 (0.3)	–
**Do you have knowledge on larvicides?**						
YES	Not asked	79(44.4)	10(12.2)	65(37.1)	154 (35.4)	0.05
NO		98(55.1)	72(87.8)	104(59.4)	274 (63)	
Don’t know		1(0.6)	0(0.0)	6(3.4)	7 (1.6)	

Note: Some of the questions had the option for multiple answers. Values are n(%). p-value by using Chi-square test.

**Table 3.** Perception of and attitude towards malaria and vector control

	**Treatment arms (%)**					
	**Untreated control** **183**	**Guided treatment**	**Full treatment semi-urban**	**Full treatment**	**Total**	***p*-value**
	**183**	**180**	**91**	**180**	**634**	
**Can reducing mosquitoes help in reducing malaria?**						
Yes	180(97.8)	172(96.1)	87(95.6)	179(99.4)	618 (97.5)	0.100
No	2(1.1)	6(3.4)	4(4.4)	1(0.6)	13 (2.0)	
Don’t know	2(1.1)	1(0.6)	0(0.0)	0(0.0)	3 (0.5)	
**What do you think which activities can reduce the presence of mosquito larvae?**						
Drain/dry up stagnant waters	115(63.9)	118(66.7)	75(82.4)	88(50.0)	409 (64.6)	0.0001
Cut grass/bushes around houses	33(18.3)	9(5.1)	3(3.3)	11(6.3)	84 (13.3)	0.0001
Use of larvicides	76(42.2)	137(77.4)	70(76.9)	134(76.1)	219 (34.6)	0.0001
Remove waste from patios	90(50.0)	68(38.4)	34(37.4)	39(22.2)	313 (49.4)	0.0001
Don´t know	14(7.8)	7(4.0)	3(3.3)	6(3.4)	219 (34.6)	0.205
**What do you think can reduce the number of mosquitoes?**						
Drain/dry up stagnant waters	117(63.9)	119(66.1)	80(87.9)	93(52.0)	409 (64.6)	0.0001
Cut grass/bushes around houses	36(19.7)	20(11.1)	14(15.4)	14(7.8)	84 (13.3)	0.007
Use of insecticides	77(42.1)	37(20.6)	57(62.6)	48(26.8)	219 (34.6)	0.0001
Use of larvicides	40(21.9)	111(61.7)	38(41.8)	124(69.3)	313 (49.4)	0.0001
Remove waste from patios	76(41.5)	60(33.3)	38(41.8)	45(25.1)	219 (34.6)	0.004
Use of mosquito nets	108(59.0)	98(54.4)	46(50.5)	93(52.0)	345 (54.5)	0.466
**n**	**–**	**180**	**91**	**180**	**451**	
**Do you trust/believe in larvicides?**						
YES	Not asked	169(93.9)	72(79.1)	159(88.3)	400 (88.7)	0.082
NO		4(2.2)	6(6.6)	7(3.9)	17 (3.8)	
Don’t know		7(3.9)	13(14.6)	14(7.8)	34 (7.5)	
**Do you think that larvicide application will reduce the risk for infection with malaria?**						
YES	Not asked	174(97.6)	70(76.9)	163(90.6)	407 (90.2)	0.0001
NO		2(1.1)	13(14.3)	4(2.2)	19 (4.2)	
Don’t know		4(2.2)	8(8.8)	13(7.2)	25 (5.5)	
**Do you trust that the larvicide is inoffensive against humans and animals?**						
Very probable	Not Asked	68(37.8)	26(28.6)	78(43.3)	172 (38.1)	0.0001
Probable		4(2.2)	36(39.6)	23(12.8)	63 (14.0)	
Neutral		8(4.4)	20 (22.0)	15 (8.3)	43 (9.5)	
Not Probable		33(18.3)	9 (9.9)	30 (16.7)	72 (16.0)	
Very important		67(37.2)	0(0.0)	34 (18.9)	101 (22.4)	
**Do you think transmission risk of malaria is more understood after the intervention?**						
YES	Not Asked	120(66.7)	51 (56.0)	125 (69.4)	296 (65.6)	0.0001
NO		49 (27.2)	8 (8.8)	37 (20.6)	94 (20.8)	
No change		11 (6.1)	32 (35.2)	18 (10.0)	61 (13.5)	

Note: Values are n(%). p-value by using Chi-square test.

**Table 4.** Practices regarding malaria and vector control

	Treatment arms (%)					
	Untreated control	Guided treatment	Full treatment semi-urban	Full treatment	Total	p
**n = 634**	**183**	**180**	**91**	**180**	**634**	
**What do you do to protect yourself against malaria?**						
Sleep under a bed net	160(87.4)	175(97.2)	86(95.6)	165(91.7)	586 (92.6)	0.002
Take medication	13(7.1)	5(2.8)	19(21.1)	6(3.3)	43 (6.8)	0.0001
Sanitize living conditions	117(63.9)	97(53.9)	62(68.9)	106(58.9)	382 (60.3)	0.070
Spray chemicals	35(19.1)	16(8.9)	14(15.6)	22(12.2)	87 (13.7)	0.034
Apply repellents on skin or clothes	7(3.8)	1(0.6)	4(4.4)	1(0.6)	13 (2.1)	–
Use mosquito coils	32(17.5)	44(24.4)	20(22.2)	36(20.0)	132 (20.9)	0.416
Nothing	2(1.1)	1(0.6)	1(1.1)	1(0.6)	5 (0.8)	–
Other	39(21.3)	10(5.6)	5(5.6)	17(9.4)	71 (11.2)	0.001
Insecticide spraying outside HH	6(3.3)	10(5.6)	0(0.0)	6(3.3)	22 (3.5)	–
Don´t know	0(0.0)	2(1.1)	0(0.0)	1(0.6)	3 (0.5)	–
**Do you use mosquito nets in your household?**						
YES	175(95.6)	175 (97.2)	86(94.5)	164 (91.1)	600 (94.6)	0.074
NO	8(4.4)	5(2.8)	5(5.5)	16(8.9)	34 (5.4)	
n		**180**	**91**	**180**	**451**	
**Would you/did you grant access to the sprayers to treat water bodies close to you house?**	Not Asked					
YES		177(98.3)	78 (85.7)	171 (95.0)	426 (94.5)	0.001
NO		2(1.1)	1 (1.1)	3(1.7)	6 (1.3)	
Don’t know		1 (0.6)	12 (13.2)	6 (3.3)	19 (4.2)	
**Would you be willing to financially contribute to such a project and with how much per year? (n = 446)**	Not Asked					
100		22 (12.2)	11 (12.6)	25 (14.0)	58 (13)	0.119
200		47 (26.1)	14 (16.1)	44 (24.6)	105 (23.5)	
500		65 (36.1)	41 (47.1)	70 (39.1)	176 (39.5)	
800		0 (0.0)	3 (3.4)	1 (0.6)	4 (0.9)	
1000		46 (25.6)	18 (20.7)	39 (21.8)	103 (23.1)	
**Would you be willing to personally participate in spraying activities**						
YES	Not Asked	167 (92.8)	62 (68.1)	163 (90.6)	392 (86.9)	0.0001
NO		9 (5.0)	19 (20.9)	10 (5.6)	38 (8.4)	
Depends on payment		4 (2.2)	10 (11.0)	7 (3.9)	21 (4.7)	
**How is your utilization of mosquito nets compared to before the intervention?**	Not Asked					
More often		61 (33.9)	16 (17.6)	75 (41.7)	152 (33.7)	0.0001
No change		49 (27.2)	58 (63.7)	54 (30.0)	161 (35.7)	
Less often		70 (38.9)	17 (18.7)	51 (28.3)	138 (30.6)	

Note: Values are n(%). p-value by using Chi-square test.

A majority of the respondents demonstrated a high level of understanding with regard to personal protective measures against mosquito bites. Over 90% reported sleeping under a bed net and 64% stated they sanitize the living space. However, very few identified such vector control measures to protect against malaria as spraying chemicals (17.2%), use of mosquito coils (13.9%), or repellents (5.8%) (Table 2). The majority of the respondents were not aware that larvicidal approaches for mosquito control exist (63%).

## Understanding of malaria vector control

Almost all respondents (97.5%) agreed that reducing mosquito populations will reduce disease prevalence. In line with their perception of what favors larval/mosquito populations, the majority (64.6%) mentioned draining of stagnant waters as an effective way to reduce mosquito populations, and more than half of the respondents identified the removal of waste from patios.

Over 88.7% of respondents expressed trust in the effectiveness of larvicidal chemicals for the control of malaria, which was not reflected in their understanding of the use of chemicals as a vector control mechanism. A similar proportion across all study arms (90.2%) reported that larvicidal activities may reduce the risk for malaria infection. Few respondents (38.1%) considered larvicides as harmful for humans and animals, although respondents from the semi-urban area expressed less concern compared to their rural counterparts. A majority of the respondents (65.6%) thought that the carrying out of larvicidal activities within the region improved their understanding of malaria transmission and its control.

## Personal protective action against malaria

A majority reported the use of bed nets (92.6%) and improving sanitation around the homes (60.3%) as the main personal and household-based protective measures against malaria, while only 20.9% stated using mosquito coils. In line with the general understanding of the role of stagnant waters for malaria transmission, a majority (97.7%) of the participants from the villages treated with Bti, were willing to grant access to sprayers to treat waterbodies close to their homes. This understanding was similar across the urban and rural households. Similarly, a majority (86.9%) were also willing to participate in the spraying activities themselves indicative of their acceptance of the approach.

When asked about their willingness to pay for larvicidal activities, a majority (63.5%) was willing to pay between FCFA 500–1000 (US$ 0.85–1.70 in Sept. 2015/€ 0.76–1.52 pegged) annually, indicative of community willingness to contribute. Around 40% were willing to contribute with FCFA 500 per year. For the semi-urban setting in Nouna, this share was higher, at around 47% of respondents. Contributions of FCFA 1000 and FCFA 200 were named by 23% of respondents each.

## Acceptance of *Bti* as a vector control measure (intervention villages only)

Almost all respondents in villages with performed treatment (99.1%) were aware of the ongoing Bti-larviciding campaign in their village (Table 5). Most witnessed larviciding at least once during the 2 years (83.5%). The majority (96.6%) rated the usefulness of the activity as good or very good at individual as well as community level and a similar proportion thought that the sprayers performed their tasks correctly (95.5%). More than 88.8% of the respondents confirmed the presence of mosquitoes at the time of the intervention, and 87.6% reported observing changes thereafter. More than half (62.7%) of them thought the mosquito numbers had gone down with 26.2% suggesting them to be much less as compared to the previous years. On the other hand, a small proportion (8.2%) reported no change in mosquito abundance after the intervention. This was also reflected in their perception of its effect on the number of malaria cases in their village. More than 66.2% reported them to have reduced, 19.9% reported them to be much less and 11.4% suggesting no observed changes. Very few respondents suggested that the number of mosquitoes (3.1%) and malaria cases (2.5%) had increased post intervention.

**Table 5.** Awareness and acceptance of the vector control activities

	**Treatment arms (%)**				
	**Guided treatment**	**Full treatment semi-urban**	**Full treatment**	**Total**	p-value
**n = 451**	**180**	**91**	**180**	**451**	
**Did you know about larviciding actions taking place in your village to eliminate mosquitoes?**					
YES	180(100.0)	87(95.6)	180(100.0)	447 (99.1)	0.0168
NO	0(0.0)	4(4.4)	0(0.0)	4 (0.9)	
**Did you already observe community-based sprayers in you village/sector?**					
YES	177(98.3)	85 (93.4)	179(99.4)	441 (99.4)	0.005
NO	3(1.7)	6(6.6)	1 (0.6)	10 (2.2)	
**How did you hear the first time about this activity?**					
Saw it myself	150(83.3)	76(87.4)	151(83.9)	377 (84.3)	0.671
By someone from my village	15(8.3)	20(23.0)	17(9.4)	52 (11.6)	0.003
By a household member	10(5.6)	12(13.8)	8(4.4)	30 (6.7)	0.054
By a health worker	24(13.3)	0(0.0)	28(15.0)	51 (11.4)	0.0001
From the radio	6(3.3)	0(0.0)	2(1.1)	8 (1.8)	–
Other	4(2.2)	0(0.0)	7(3.9)	11 (2.5)	–
**What do you think about this intervention? (n = 444)**					
Very good	112(62.6)	15(17.6)	83(46.1)	210 (47.3)	0.0001
Good	63(35.2)	65(76.5)	91(50.6)	219 (49.3)	
Ok/BAD	4(2.2)	5(5.9)	6(3.4)	15 (3.5)	
**What do you think is the community’s opinion on this intervention?**					
Very good	94(52.2)	15 (16.5)	69(38.3)	178 (39.5)	0.0001
Good	80(44.4)	70 (76.9)	105 (58.3)	255 (56.5)	
Ok	6(3.3)	6(6.6)	6(3.3)	18 (4)	
**Did you observe any changes after the intervention started? (n = 436)**					
YES	166(93.8)	59(73.8)	157(87.7)	382 (87.6)	0.01
NO	9(5.1)	16(20.0)	15(8.4)	40 (9.2)	
Don’t know	2(1.1)	5(6.3)	7(3.9)	14 (3.2)	
**Do you think the sprayers are performing their tasks correctly?**					
YES	179(99.4)	77 (84.4)	175 (97.2)	431 (95.6)	0.0001
NO	0(0.0)	2(2.2)	2(1.1)	4 (0.8)	
Don’t know	1(0.6)	12(13.3)	3(1.7)	16 (3.6)	
**Are there mosquitoes in your village or sector?**					
YES	148(82.2)	84 (92.3)	165(91.7)	397 (88.0)	0.0001
NO	32(17.8)	3(3.3)	14(7.8)	49 (10.9)	
Don’t know	0(0.0)	4 (4.4)	1(0.6)	5 (1.1)	
**How do you perceive mosquito abundance compared to the preceding years?**					
Much less	71(39.4)	16(17.6)	30(16.7)	117 (25.9)	0.0001
Less	104(57.8)	55(60.4)	124(68.9)	283 (62.7)	
No change	4(2.2)	20(22.0)	13(7.2)	37 (8.2)	
More+ Much more	1 (0.6)	0(0.0)	13 (7.2)	14 (3.1)	
**How do you perceive the number of malaria cases compared to the last years?** **(n = 447)**					
Much less	56(31.1)	7(8.0)	26(14.4)	89 (19.9)	0.0001
Less	115(63.9)	54(62.1)	127(70.6)	296 (66.2)	
No change	9(5.0)	23(26.4)	19(10.6)	51 (11.4)	
More	0(0.0)	0(0.0)	3(100.0)	3 (0.7)	
Much more	0(0.0)	3(3.4)	5(2.8)	8 (1.8)	

Note: For questions that were not answered by all respondents, the number of respondents is indicated. Values are n(%). p-value by using Chi-square test.

## Discussion

The results show that most of the people within the greater Nouna region have basic to good knowledge of malaria transmission and can link it to the adult mosquitoes as well as to factors that favor their development and survival, such as stagnant water, rainfall and humidity. Most of the respondents correctly identified stagnant water and pit latrines as breeding sites for mosquitoes, while the concept of mosquitoes developing through larval stages was only known to less than half of the respondents. Although many people are not aware of the life cycle of mosquitoes or can differentiate between species that transmit malaria and those that do not, an understanding of mosquito- and transmission prone environments seems to be very present. This is in line with observations from other studies [17,28–30], while only few research reported the link between mosquitoes and malaria infection being not correctly identified by the majority of interviewees [31]. Generally, knowledge on classical personal protective measures against malaria, such as bed nets, sanitized living conditions, chemicals and repellents was good, while most people were not aware about the approach of larviciding before the intervention had started. This is likely to have its reason in the fact that this was the first use of larviciding in the region, while other interventions have been introduced through distribution campaigns or were locally used in scientific trials. Similar observations were made by Mboera and colleagues who ascribed the lack of knowledge on larviciding to the low level of community involvement in the trial preceding their own study [17]. This indicates that scientific or routine interventions are likely to have an informative and educational role in malaria control, which should be taken advantage of when designing and carrying out such interventions.

Equally, there was awareness of the need for individual protective measures against the vector and the disease, which is in line with findings from a qualitative study in the same region, that used in-depth interviews and focus group discussions [18]. However, good knowledge and awareness about the importance and effectiveness of preventive measures is not an indicator for their use and can be substantially lower [29]. Almost all respondents either already used mosquito nets in their household or ascribed high importance to them, which was equally observed in a survey on bed net use in the region [22]. Interestingly, the effect of spraying interventions on the personal use of mosquito nets was diverse. While roughly a third of the respondents used them more often after the intervention had started, another third used them less often, while for the rest, no change was reported. Two different effects could be the underlying cause for this heterogeneity, on the one hand, an increased awareness of malaria transmission and hence an increased use of bed nets and, on the other hand, a feeling of safety that led to a decreased use among some participants. This phenomenon was equally reported from a study in Dar es Salaam, Tanzania, where bed net use declined by 5% following larviciding interventions [16]. The perception of the importance of malaria control measures is likely to change with the perceived risk of contracting malaria. Furthermore, several studies have shown that, as the level of malaria transmission decreases, so does the perception about the importance of control activities [29,32].

When asked about the change in mosquito abundance compared to the pre-intervention year, most respondents answered with ‘much less’ and ‘less’. These perceived high reductions are reflected by the catches of adult female *Anopheles* mosquitoes in light traps in the same villages that were found to be 61% (guided treatment) and 70% (full treatment) lower compared to before the intervention [6]. In the semi-urban town of Nouna, however, almost 20% of respondents reported no change in perceived mosquito abundance compared to the preceding years. Catches in light traps showed that the mosquito abundance in Nouna was indeed higher compared to the rural villages but a high share of the mosquito genera caught were non-malaria transmitting *Culex* mosquitoes that breed in the strongly polluted typical semi-urban breeding sites, such as pit latrines and dirty puddles and *Aedes* mosquitoes that breed in likewise typically semi-urban habitats, such as water filled pots, old car tires, and drinking water containers. Many of those breeding habitats are particularly present within private compounds, where spraying activities were not carried out. Future interventions need to take this into account and consider extending spraying activities to nuisance and non-*Anopheles* mosquitoes in private compounds, which would be generally welcomed by the overwhelming majority of participants (98%). This is important because it is likely to impact people’s perception on the success of larviciding interventions, since they cannot distinguish different mosquito genera and assess their potential risk for malaria transmission. Furthermore, this extended larviciding would allow for a more synergistic targeting of vectors of other mosquito-borne diseases, such as dengue, Zika, yellow fever and filariasis [33,34].

The population’s willingness to financially contribute to larviciding interventions was strong, and more than 20% of the respondents indicated that they were willing to pay a yearly fee, which would fully cover and even surpass the annual per capita intervention costs for Bti-based larviciding [13]. The high observed willingness to pay obtained through questionnaire-based interviews was reported similarly during focus group discussions and in-depth interviews in the same study region [18]. A study from urban Tanzania equally reported a high willingness to contribute in a larviciding program, while interviewees raised concerns about the correct use of the money and corruption [17]. Answers to questions on willingness to pay need to be interpreted with care because of the hypothetical nature of the question and the possible deviating behavior when it comes to actual payment. However, the findings presented here are advocating for a strong involvement of communities for sustaining locally implemented vector control activities, such as larviciding. A couple of important prerequisites seem important to be met: i) the intervention needs to be carried out by a trustworthy source, such as the MoH, or a regional government, ii) ideally it should be financed by external donors for some time because the population is unlikely to pay for an intervention that still has to be proven beneficial for them, iii) it needs to be monitored and evaluated to inform the population about its effectiveness and facilitate their decision to buy in and keep supporting it [24].

A strength of this study is its sample size, comprised of the guided administration of 634 individual questionnaires in a total of 36 rural villages and the village of Nouna. The villages in which questionnaires were administered were the same as those that were randomly chosen for mosquito catches, which allows to compare the perceived mosquito reduction with those measured using light traps. Limitations of this study include the following: Since in several cases, questionnaire administration was carried out when other members of the family were present, participants might have felt pressure to answer in favor of the larviciding project. Similarly, respondents might have felt influenced by the presence of interviewers from outsidethe communities. Our study results show that the performed larviciding intervention showed positive resonance among the population in villages and the semi-urban town of Nouna. People reported a significant drop in mosquito abundance and are positively minded towards the program. People showed willingness to involve and willingness to contribute financially, some with an amount that would fully cover the intervention costs. People would welcome a routine implementation and continuation of such a larviciding program.

## Supplementary Material

Supplemental Material

## Figures and Tables

**Table:** 

*Bs*	*Bacillus sphaericus*
*Bti*	*Bacillus thuringiensis israelensis*
CSPS	Centre de Santé et Promotion Sociale (Rural Health Center)
EIR	Entomological Inoculation Rate
EMIRA	Ecologic Malaria Reduction for Africa
IPTp	Intermittent Preventive Treatment in pregnancy
IRS	Indoor Residual Spraying
ITN	Insecticide Treated Net
LLIN	Long Lasting Insecticidal Net
LSM	Larval Source Management

## Paper context

Biological larviciding against malaria vectors is an additional option to support current vector control strategies that are implemented on the personal or household level (ITNs, repellants, window screens). However, little is known about the perception and acceptability of people towards approaches that depend on the backing of an entire community or village.

## Additional material

Additional file 1. Questionnaire (English)
