# Supplementary material for: Community acceptance of environmental larviciding against malaria with Bacillus thuringiensis israelensis in rural Burkina Faso – A knowledge, attitudes and practices study
Source: Glob Health Action. 2021 Dec 20;14(1):1988279. doi: 10.1080/16549716.2021.1988279 (PMC8725727; doi:10.1080/16549716.2021.1988279)
Supplement: Supplemental Material [file ZGHA_A_1988279_SM4755.docx]

**Supplementary materials**

Supplementary content 1: Questionnaire (English translation of the original, French version)

|  | | | |  | | | | | | | |  | | | | | | | | | | | | | | | | |  | | | | | | | |  | | | | | | | | | | | | | | | | | | | |  | | | | | | | | | | |  | | | | | | |  | | | | | | | | | | | | | |  | | | | | | | | | | | |  | | | | | | | | | | | | | | | | | | | | | | | | |  | | | | | | | | | | | | | | | |  | | | | | | | | | | | |  | | | | | | | | | | | | | | | | | | |  | | | | | | | | | | | | | | | |  | | | | | | | |  | | | | | | | | | | | | | | |  | | | | | | | | | | | | | | | | | | | | | | | | | |  | | | | | | | | | | | |  | | | | | | | | | | | | | | | | | | |  | | | | | | | | | | | | |  | | | | | | | |  | | | | | | | | | | | | | | | | | | | | | | |  | | | | | | | | | | | | | | | | | | |  | | | | | | | | | | | |  | | | | | | | | | | | |  | | | | | | | | | | | | |  | | | | | | | | | | | | | | | | | | | | | |  | | | | | | | | | | | | | | |  | | | | | | | | | | | | | | | | |  | | | | | | | | | | | | | | | | |  | | | | | | | | | | | | | | | |  |  |  |  |  |  |  |  |  |  |  |  |  |  |  |  |  |
| --- | --- | --- | --- | --- | --- | --- | --- | --- | --- | --- | --- | --- | --- | --- | --- | --- | --- | --- | --- | --- | --- | --- | --- | --- | --- | --- | --- | --- | --- | --- | --- | --- | --- | --- | --- | --- | --- | --- | --- | --- | --- | --- | --- | --- | --- | --- | --- | --- | --- | --- | --- | --- | --- | --- | --- | --- | --- | --- | --- | --- | --- | --- | --- | --- | --- | --- | --- | --- | --- | --- | --- | --- | --- | --- | --- | --- | --- | --- | --- | --- | --- | --- | --- | --- | --- | --- | --- | --- | --- | --- | --- | --- | --- | --- | --- | --- | --- | --- | --- | --- | --- | --- | --- | --- | --- | --- | --- | --- | --- | --- | --- | --- | --- | --- | --- | --- | --- | --- | --- | --- | --- | --- | --- | --- | --- | --- | --- | --- | --- | --- | --- | --- | --- | --- | --- | --- | --- | --- | --- | --- | --- | --- | --- | --- | --- | --- | --- | --- | --- | --- | --- | --- | --- | --- | --- | --- | --- | --- | --- | --- | --- | --- | --- | --- | --- | --- | --- | --- | --- | --- | --- | --- | --- | --- | --- | --- | --- | --- | --- | --- | --- | --- | --- | --- | --- | --- | --- | --- | --- | --- | --- | --- | --- | --- | --- | --- | --- | --- | --- | --- | --- | --- | --- | --- | --- | --- | --- | --- | --- | --- | --- | --- | --- | --- | --- | --- | --- | --- | --- | --- | --- | --- | --- | --- | --- | --- | --- | --- | --- | --- | --- | --- | --- | --- | --- | --- | --- | --- | --- | --- | --- | --- | --- | --- | --- | --- | --- | --- | --- | --- | --- | --- | --- | --- | --- | --- | --- | --- | --- | --- | --- | --- | --- | --- | --- | --- | --- | --- | --- | --- | --- | --- | --- | --- | --- | --- | --- | --- | --- | --- | --- | --- | --- | --- | --- | --- | --- | --- | --- | --- | --- | --- | --- | --- | --- | --- | --- | --- | --- | --- | --- | --- | --- | --- | --- | --- | --- | --- | --- | --- | --- | --- | --- | --- | --- | --- | --- | --- | --- | --- | --- | --- | --- | --- | --- | --- | --- | --- | --- | --- | --- | --- | --- | --- | --- | --- | --- | --- | --- | --- | --- | --- | --- | --- | --- | --- | --- | --- | --- | --- | --- | --- | --- | --- | --- | --- | --- | --- | --- | --- | --- | --- | --- | --- | --- | --- | --- | --- | --- | --- | --- | --- | --- | --- | --- | --- | --- | --- | --- | --- | --- | --- | --- | --- | --- | --- | --- | --- | --- | --- | --- | --- | --- | --- | --- | --- | --- | --- | --- | --- | --- | --- | --- | --- | --- | --- | --- | --- | --- | --- | --- | --- | --- | --- | --- | --- | --- | --- | --- | --- | --- | --- | --- | --- | --- | --- | --- | --- | --- | --- | --- | --- | --- | --- | --- | --- | --- | --- | --- | --- | --- | --- | --- | --- | --- | --- | --- | --- | --- | --- | --- | --- | --- | --- | --- | --- | --- | --- | --- | --- | --- | --- | --- | --- | --- | --- | --- | --- | --- | --- | --- | --- |
|  | | | |  | | | | | | | |  | | | | | | | | | | | | | | | | |  | | | | | | | |  | | | | | | | | | | | | | | | | | | | |  | | | | | | | | | | |  | | | | | | |  | | | | | | | | | | | | | |  | | | | | | | | | | | |  | | | | | | | | | | | | | | | | | | | | | | | | |  | | | | | | | | | | | | | | | |  | | | | | | | | | | | |  | | | | | | | | | | | | | | | | | | |  | | | | | | | | | | | | | | | |  | | | | | | | |  | | | | | | | | | | | | | | |  | | | | | | | | | | | | | | | | | | | | | | | | | |  | | | | | | | | | | | |  | | | | | | | | | | | | | | | | | | |  | | | | | | | | | | | | |  | | | | | | | |  | | | | | | | | | | | | | | | | | | | | | | |  | | | | | | | | | | | | | | | | | | |  | | | | | | | | | | | |  | | | | | | | | | | | |  | | | | | | | | | | | | |  | | | | | | | | | | | | | | | | | | | | | |  | | | | | | | | | | | | | | |  | | | | | | | | | | | | | | | | |  | | | | | | | | | | | | | | | | |  | | | | | | | | | | | | | | | |  |  |  |  |  |  |  |  |  |  |  |  |  |  |  |  |  |
| **CENTRE DE RECHERCHE EN SANTE DE NOUNA** | | | | | | | | | | | | | | | | | | | | | | | | | | | | | | | | | | | | | | | | | | | | | | | | | | | | | | | | | | | | | | | | | | | | | | | | | | | | | | | | | | | | | | | | | | | | | | | | | | | | | | | | | | | | | | | | | | | | | | | | | | | | | | | | | | | | | | | | | |  | | | | | | | | | | | | | | |  | | | | | | | | | | | | | | |  | | | | | | | | | | | | |  | | | | | | | | | | | | | | | | | | | | | | | | |  | | | | | | | | | | | | |  | | | | | | | | | | | |  | | | | | | | | | | | | | | | | | | | | | | | **BURKINA FASO** | | | | | | | | | | | | | | | | | | | | | | | | | | | | | | | | | | | | | | | | | | | | | | | | | | | | | | |  | | | | | | | | | | | | | | | | |  | | | | | | | | | | | | | | | | | |  | | | | | | | | | | | |  | | | | | | | | | | | | | | | | | |  | | | | | | | | | | | | |  | | | | | | | | | | | | | | | | | |  | | | | | | | | | | | | | | | | | |  | | | | | | | | | | | | | | | | |  | | | | | | | | | | | | | | | | | | | | |  | | | | | | | | | | | |
|  | | | |  | | | | | | | |  | | | | | | | | | | | | | | | | |  | | | | | | | |  | | | | | | | | | | | | | | | | | | | |  | | | | | | | | | | |  | | | | | | |  | | | | | | | | | | | | | |  | | | | | | | | | | | |  | | | | | | | | | | | | | | | | | | | | | | | | |  | | | | | | | | | | | | | | | |  | | | | | | | | | | | |  | | | | | | | | | | | | | | | | | | |  | | | | | | | | | | | | | | | |  | | | | | | | |  | | | | | | | | | | | | | | |  | | | | | | | | | | | | | | | | | | | | | | | | | | Unité-Progrès-Justice | | | | | | | | | | | | | | | | | | | | | | | | | | | | | | | | | | | | | | | | | | | | | | | | | | | | | | | | | | | | | | | | | | | | | | | | | | | | | | | | | | | | | | | | | | | | | |  | | | | | | | | | | | |  | | | | | | | | | | | |  | | | | | | | | | | | | |  | | | | | | | | | | | | | | | | | | | | | |  | | | | | | | | | | | | | | |  | | | | | | | | | | | | | | | | |  | | | | | | | | | | | | | | | | |  | | | | | | | | | | | | | | | |  |  |  |  |  |  |  |  |  |  |  |  |  |  |  |  |  |
|  | | | |  | | | | | | | |  | | | | | | | | | | | | | | | | |  | | | | | | | |  | | | | | | | | | | | | | | | | | | | |  | | | | | | | | | | |  | | | | | | |  | | | | | | | | | | | | | |  | | | | | | | | | | | |  | | | | | | | | | | | | | | | | | | | | | | | | |  | | | | | | | | | | | | | | | |  | | | | | | | | | | | |  | | | | | | | | | | | | | | | | | | |  | | | | | | | | | | | | | | | |  | | | | | | | |  | | | | | | | | | | | | | | |  | | | | | | | | | | | | | | | | | | | | | | | | | |  | | | | | | | | | | | |  | | | | | | | | | | | | | | | | | | |  | | | | | | | | | | | | |  | | | | | | | |  | | | | | | | | | | | | | | | | | | | | | | |  | | | | | | | | | | | | | | | | | | |  | | | | | | | | | | | |  | | | | | | | | | | | |  | | | | | | | | | | | | |  | | | | | | | | | | | | | | | | | | | | | |  | | | | | | | | | | | | | | |  | | | | | | | | | | | | | | | | |  | | | | | | | | | | | | | | | | |  | | | | | | | | | | | | | | | |  |  |  |  |  |  |  |  |  |  |  |  |  |  |  |  |  |
|  | | | |  | | | | | | | |  | | | | | | | | | | | | | | | | |  | | | | | | | |  | | | | | | | | | | | | | | | | | | | |  | | | | | | | | | | |  | | | | | | |  | | | | | | | | | | | | | |  | | | | | | | | | | | |  | | | | | | | | | | | | | | | | | | | | | | | | |  | | | | | | | | | | | | | | | |  | | | | | | | | | | | |  | | | | | | | | | | | | | | | | | | |  | | | | | | | | | | | | | | | |  | | | | | | | |  | | | | | | | | | | | | | | |  | | | | | | | | | | | | | | | | | | | | | | | | | |  | | | | | | | | | | | |  | | | | | | | | | | | | | | | | | | |  | | | | | | | | | | | | |  | | | | | | | |  | | | | | | | | | | | | | | | | | | | | | | |  | | | | | | | | | | | | | | | | | | |  | | | | | | | | | | | |  | | | | | | | | | | | |  | | | | | | | | | | | | |  | | | | | | | | | | | | | | | | | | | | | |  | | | | | | | | | | | | | | |  | | | | | | | | | | | | | | | | |  | | | | | | | | | | | | | | | | |  | | | | | | | | | | | | | | | |  |  |  |  |  |  |  |  |  |  |  |  |  |  |  |  |  |
| **Community Knowledge and Acceptance of Larviciding for** | | | | | | | | | | | | | | | | | | | | | | | | | | | | | | | | | | | | | | | | | | | | | | | | | | | | | | | | | | | | | | | | | | | | | | | | | | | | | | | | | | | | | | | | | | | | | | | | | | | | | | | | | | | | | | | | | | | | | | | | | | | | | | | | | | | | | | | | | | | | | | | | | | | | | | | | | | | | | | | | | | | | | | | | | | | | | | | | | | | | | | | | | | | | | | | | | | | | | | | | | | | | | | | | | | | | | | | | | | | | | | | | | | | | |  | | | | | | | | | | | | | | | | | | | | |  | | | | | | | | | | | | | | | |  | | | | | | | | | | | | | |  | | | | | | | | | | | | | | |  | | | | | | | | | | |  | | | | | | | | | | | | | | | | |  | | | | | | | | | | | | | | | | | | |  | | | | | | | | | | | | | |  | | | | | | | | | | | | | | | | | |  | | | | | | | | | | | | |  | | | | | | | | | | | | | | | | | |  | | | | | | | | | | | | | | | | | |  | | | | | | | | | | | | | | | | |  | | | | | | | | | | | | | | | | | | | | |  | | | | | | | | | | | |
| **Malaria Control in a Rural Area In Burkina Faso_ Nouna Health District** | | | | | | | | | | | | | | | | | | | | | | | | | | | | | | | | | | | | | | | | | | | | | | | | | | | | | | | | | | | | | | | | | | | | | | | | | | | | | | | | | | | | | | | | | | | | | | | | | | | | | | | | | | | | | | | | | | | | | | | | | | | | | | | | | | | | | | | | | | | | | | | | | | | | | | | | | | | | | | | | | | | | | | | | | | | | | | | | | | | | | | | | | | | | | | | | | | | | | | | | | | | | | | | | | | | | | | | | | | | | | | | | | | | | | | | | | | | | | | | | | | | | | | | | | | | | | | | | | | | | | | | | | | | | | | | | | | | | | | | |  | | | | | | | | | | | | | | |  | | | | | | | | | | |  | | | | | | | | | | | | | | | | |  | | | | | | | | | | | | | | | | | | |  | | | | | | | | | | | | | |  | | | | | | | | | | | | | | | | | |  | | | | | | | | | | | | |  | | | | | | | | | | | | | | | | | |  | | | | | | | | | | | | | | | | | |  | | | | | | | | | | | | | | | | |  | | | | | | | | | | | | | | | | | | | | |  | | | | | | | | | | | |
|  | | | |  | | | | | | | |  | | | | | | | | | | | | | | | | |  | | | | | | | |  | | | | | | | | | | | | | | | | | | | |  | | | | | | | | | | |  | | | | | | |  | | | | | | | | | | | | | |  | | | | | | | | | | | |  | | | | | | | | | | | | | | | | | | | | | | | | |  | | | | | | | | | | | | | | | |  | | | | | | | | | | | |  | | | | | | | | | | | | | | | | | | |  | | | | | | | | | | | | | | | |  | | | | | | | |  | | | | | | | | | | | | | | |  | | | | | | | | | | | | | | | | | | | | | | | | | |  | | | | | | | | | | | |  | | | | | | | | | | | | | | | | | | |  | | | | | | | | | | | | |  | | | | | | | |  | | | | | | | | | | | | | | | | | | | | | | |  | | | | | | | | | | | | | | | | | | |  | | | | | | | | | | | |  | | | | | | | | | | | |  | | | | | | | | | | | | |  | | | | | | | | | | | | | | | | | | | | | |  | | | | | | | | | | | | | | |  | | | | | | | | | | | | | | | | |  | | | | | | | | | | | | | | | | |  | | | | | | | | | | | | | | | |  |  |  |  |  |  |  |  |  |  |  |  |  |  |  |  |  |
|  | | | |  | | | | | | | |  | | | | | | | | | | | | | | | | |  | | | | | | | |  | | | | | | | | | | | | | | | | | | | |  | | | | | | | | | | |  | | | | | | |  | | | | | | | | | | | | | |  | | | | | | | | | | | |  | | | | | | | | | | | | | | | | | | | | | | | | |  | | | | | | | | | | | | | | | |  | | | | | | | | | | | |  | | | | | | | | | | | | | | | | | | |  | | | | | | | | | | | | | | | |  | | | | | | | |  | | | | | | | | | | | | | | |  | | | | | | | | | | | | | | | | | | | | | | | | | |  | | | | | | | | | | | |  | | | | | | | | | | | | | | | | | | |  | | | | | | | | | | | | |  | | | | | | | |  | | | | | | | | | | | | | | | | | | | | | | |  | | | | | | | | | | | | | | | | | | |  | | | | | | | | | | | |  | | | | | | | | | | | |  | | | | | | | | | | | | |  | | | | | | | | | | | | | | | | | | | | | |  | | | | | | | | | | | | | | |  | | | | | | | | | | | | | | | | |  | | | | | | | | | | | | | | | | |  | | | | | | | | | | | | | | | |  |  |  |  |  |  |  |  |  |  |  |  |  |  |  |  |  |
| **Date of visit** | | | | | | | | | | | | | | | | | | | | | | |  | | | | | | | | | | | | | | | | | | | | |  | | | | | | | |  | | | | | | | | | | | |  | | | | | | | | | | | | | | | | |  | | | | | | | | | | | | |  | | | | | | | | | | | | | |  | | | | | | | | | | | |  | | | | | | | | | | | | | | | | | |  | | | | | | | | | | | | | | | |  | | | | | | | | | | | | | |  | | | | | | | | | | | | |  | | | | | | | | | | | | | | | | | | | | | | | | | |  | | | | | | | | | | | | |  | | | | | | | | | | | |  | | | | | | | | | | | | | | | | | | | | | | | |  | | | | | | | | | | |  | | | | | | | | | | | | | |  | | | | | | | | | | | | | | |  | | | | | | | | | | | | |  | | | | | | | | | | | | | | | | |  | | | | | | | | | | | | | | | | | |  | | | | | | | | | | | | |  | | | | | | | | | | | | | | | | | |  | | | | | | | | | | | | |  | | | | | | | | | | | | | | | | | | |  | | | | | | | | | | | | | | | |  | | | | | | | | | | | | | | | | | | |  | | | | | | | | | | | | | | | | | | |  | | | | | | | | | | |  |
|  | | | | |  | | | | | | | |  | | | | | | | | | | | | | | | | |  | | | | | | |  | | | | | | | | | | | | | | | | | | | |  | | | | | | | | | | |  | | | | | | |  | | | | | | | | | | | | | |  | | | | | | | | | | | |  | | | | | | | | | | | | | | | | | | | | | | | | | |  | | | | | | | | | | | | | | | |  | | | | | | | | | | | | | | |  | | | | | | | | | | | | | | | |  | | | | | | | | | | | | | | | |  | | | | | | | |  | | | | | | | | | | | | | | | |  | | | | | | | | | | | | | | | | | | | | | | | | | |  | | | | | | | | | | | |  | | | | | | | | | | | | | | | | | | |  | | | | | | | | | | | |  | | | | | | | | |  | | | | | | | | | | | | | | | | | | | | |  | | | | | | | | | | | | | | | | | | |  | | | | | | | | | | | |  | | | | | | | | | | | | |  | | | | | | | | | | | | |  | | | | | | | | | | | | | | | | | | | | | | |  | | | | | | | | | | | | | | | |  | | | | | | | | | | | | | | | | |  | | | | | | | | | | | | | | | |  | | | | | | | | | | | | | | |  |  |  |  |  |  |  |  |  |  |  |  |  |  |  |  |
| **Village Name** | | | | | | | | | | | | | | | | | | | | | | | | | | | | | | | | | | | | | | | | | | | | | | | | | | | | | | | | | | | | | | | |  | | | | | | | | | | | | | | | | |  | | | | | | | | | | | | |  | | | | | | | | | | | | | |  | | | | | | | | | | | |  | | | | | | | | | | | | | | | | | |  | | | | | | | | | | | | | | | |  | | | | | | | | | | | | | |  | | | | | | | | | | | | |  | | | | | | | | | | | | | | | | | | | | | | | | | | **Village Code** | | | | | | | | | | | | | | | | | | | | | | | | | | | | | | | | | | | | | | | | | | | | | | | | |  | | | | | | | | | | |  | | | | | | | | | | | | | |  | | | | | | | | | | | | | | |  | | | | | | | | | | | | |  | | | | | | | | | | | | | | | | |  | | | | | | | | | | | | | | | | | |  | | | | | | | | | | | | |  | | | | | | | | | | | | | | | | | |  | | | | | | | | | | | | |  | | | | | | | | | | | | | | | | | | |  | | | | | | | | | | | | | | | |  | | | | | | | | | | | | | | | | | | |  | | | | | | | | | | | | | | | | | | |  | | | | | | | | | | |  |
|  | | | | |  | | | | | | | |  | | | | | | | | | | | | | | | | |  | | | | | | |  | | | | | | | | | | | | | | | | | | | |  | | | | | | | | | | |  | | | | | | |  | | | | | | | | | | | | | |  | | | | | | | | | | | |  | | | | | | | | | | | | | | | | | | | | | | | | | |  | | | | | | | | | | | | | | | |  | | | | | | | | | | | | | | |  | | | | | | | | | | | | | | | |  | | | | | | | | | | | | | | | |  | | | | | | | |  | | | | | | | | | | | | | | | |  | | | | | | | | | | | | | | | | | | | | | | | | | |  | | | | | | | | | | | |  | | | | | | | | | | | | | | | | | | |  | | | | | | | | | | | |  | | | | | | | | |  | | | | | | | | | | | | | | | | | | | | |  | | | | | | | | | | | | | | | | | | |  | | | | | | | | | | | |  | | | | | | | | | | | | |  | | | | | | | | | | | | |  | | | | | | | | | | | | | | | | | | | | | | |  | | | | | | | | | | | | | | | |  | | | | | | | | | | | | | | | | |  | | | | | | | | | | | | | | | |  | | | | | | | | | | | | | | |  |  |  |  |  |  |  |  |  |  |  |  |  |  |  |  |
| **Village Sector** | | | | | | | | | | | | | | | | | | | | | | | | | | | | | | | | | | | | | | | | | | | |  | | | | | | | |  | | | | | | | | | | | |  | | | | | | | | | | | | | | | | |  | | | | | | | | | | | | |  | | | | | | | | | | | | | |  | | | | | | | | | | | |  | | | | | | | | | | | | | | | | | |  | | | | | | | | | | | | | | | |  | | | | | | | | | | | | | |  | | | | | | | | | | | | |  | | | | | | | | | | | | | | | | | | | | | | | | | |  | | | | | | | | | | | | |  | | | | | | | | | | | |  | | | | | | | | | | | | | | | | | | | | | | | |  | | | | | | | | | | |  | | | | | | | | | | | | | |  | | | | | | | | | | | | | | |  | | | | | | | | | | | | |  | | | | | | | | | | | | | | | | |  | | | | | | | | | | | | | | | | | |  | | | | | | | | | | | | |  | | | | | | | | | | | | | | | | | |  | | | | | | | | | | | | |  | | | | | | | | | | | | | | | | | | |  | | | | | | | | | | | | | | | |  | | | | | | | | | | | | | | | | | | |  | | | | | | | | | | | | | | | | | | |  | | | | | | | | | | |  |
|  | | | | |  | | | | | | | |  | | | | | | | | | | | | | | | | |  | | | | | | |  | | | | | | | | | | | | | | | | | | | |  | | | | | | | | | | |  | | | | | | |  | | | | | | | | | | | | | |  | | | | | | | | | | | |  | | | | | | | | | | | | | | | | | | | | | | | | | |  | | | | | | | | | | | | | | | |  | | | | | | | | | | | | | | |  | | | | | | | | | | | | | | | |  | | | | | | | | | | | | | | | |  | | | | | | | |  | | | | | | | | | | | | | | | |  | | | | | | | | | | | | | | | | | | | | | | | | | |  | | | | | | | | | | | |  | | | | | | | | | | | | | | | | | | |  | | | | | | | | | | | |  | | | | | | | | |  | | | | | | | | | | | | | | | | | | | | |  | | | | | | | | | | | | | | | | | | |  | | | | | | | | | | | |  | | | | | | | | | | | | |  | | | | | | | | | | | | |  | | | | | | | | | | | | | | | | | | | | | | |  | | | | | | | | | | | | | | | |  | | | | | | | | | | | | | | | | |  | | | | | | | | | | | | | | | |  | | | | | | | | | | | | | | |  |  |  |  |  |  |  |  |  |  |  |  |  |  |  |  |
|  | | | | |  | | | | | | | |  | | | | | | | | | | | | | | | | |  | | | | | | |  | | | | | | | | | | | | | | | | | | | |  | | | | | | | | | | |  | | | | | | |  | | | | | | | | | | | | | |  | | | | | | | | | | | |  | | | | | | | | | | | | | | | | | | | | | | | | | |  | | | | | | | | | | | | | | | |  | | | | | | | | | | | | | | |  | | | | | | | | | | | | | | | |  | | | | | | | | | | | | | | | |  | | | | | | | |  | | | | | | | | | | | | | | | |  | | | | | | | | | | | | | | | | | | | | | | | | | |  | | | | | | | | | | | |  | | | | | | | | | | | | | | | | | | |  | | | | | | | | | | | |  | | | | | | | | |  | | | | | | | | | | | | | | | | | | | | |  | | | | | | | | | | | | | | | | | | |  | | | | | | | | | | | |  | | | | | | | | | | | | |  | | | | | | | | | | | | |  | | | | | | | | | | | | | | | | | | | | | | |  | | | | | | | | | | | | | | | |  | | | | | | | | | | | | | | | | |  | | | | | | | | | | | | | | | |  | | | | | | | | | | | | | | |  |  |  |  |  |  |  |  |  |  |  |  |  |  |  |  |
| **Household ID** | | | | | | | | | | | | | | | | | | | | | | | | | | | | | | | | | | |  | | | | | | | | | |  | | | | | | | |  | | | | | | | | | | | |  | | | | | | | | | | | | | | | | |  | | | | | | | | | | | | |  | | | | | | | | | | | | | |  | | | | | | | | | | | |  | | | | | | | | | | | | | | | | |  | | | | | | | | | | | | | | | | | **Name Household Head (CHM)** | | | | | | | | | | | | | | | | | | | | | | | | | | | | | | | | | | | | | | | | | | | | | | | | | | |  | | | | | | | | | | | | |  | | | | | | | | | | | |  | | | | | | | | | | | | | | | | | | |  | | | | | | | | | | | | | | |  | | | | | | | | | | | | | | |  | | | | | | | | | | | | | | |  | | | | | | | | | | |  | | | | | | | | | | | | | | | | | |  | | | | | | | | | | | | | | | | | |  | | | | | | | | | | | | | |  | | | | | | | | | | | | | | | | | |  | | | | | | | | | | | | |  | | | | | | | | | | | | | | | | | | |  | | | | | | | | | | | | | | | | | |  | | | | | | | | | | | | | | | | |  | | | | | | | | | | | | | | | | | | | | |  | | | | | | | | | |  |
|  | | | |  | | | | | | | |  | | | | | | | | | | | | | | | | |  | | | | | | |  | | | | | | | | | | | | | | | | | | | | | |  | | | | | | | | | | |  | | | | | | |  | | | | | | | | | | | | | |  | | | | | | | | | | | |  | | | | | | | | | | | | | | | | | | | | | | | | | |  | | | | | | | | | | | | | | |  | | | | | | | | | | | | | |  | | | | | | | | | | | | | | | |  | | | | | | | | | | | | | | | |  | | | | | | | |  | | | | | | | | | | | | | | |  | | | | | | | | | | | | | | | | | | | | | | | | | | |  | | | | | | | | | | | |  | | | | | | | | | | | | | | | | | | | |  | | | | | | | | | | |  | | | | | | | | |  | | | | | | | | | | | | | | | | | | | | | |  | | | | | | | | | | | | | | | | | | |  | | | | | | | | | | | |  | | | | | | | | | | | |  | | | | | | | | | | | | |  | | | | | | | | | | | | | | | | | | | | | | |  | | | | | | | | | | | | | | | |  | | | | | | | | | | | | | | | |  | | | | | | | | | | | | | | | | |  | | | | | | | | | | | | | | |  |  |  |  |  |  |  |  |  |  |  |  |  |  |  |  |  |
| **Gender HH Head** | | | | | | | | | | | | | | | | | | | | | | | | 1.M | | | | | | | | | | | | | | | | | | | | |  | | | | | | | | 2.F | | | | | | | | | | | |  | | | | | | | | | | | | | | | | | |  | | | | | | | | | | | | |  | | | | | | | | | | | | | |  | | | | | | | | | | | |  | | | | | | | | | | | | | | | | |  | | | | | | | | | | | | | | | | |  | | | | | | | | | | | | | ID HH Head | | | | | | | | | | | | | | | | | | | | | | | | | | | | | | | | | | | | | |  | | | | | | | | | | | | |  | | | | | | | | | | | |  | | | | | | | | | | | | | | | | | | | | | | | |  | | | | | | | | | | |  | | | | | | | | | | | | | |  | | | | | | | | | | | | | | |  | | | | | | | | | | | | |  | | | | | | | | | | | | | | | | |  | | | | | | | | | | | | | | | | | |  | | | | | | | | | | | | |  | | | | | | | | | | | | | | | | | |  | | | | | | | | | | | | |  | | | | | | | | | | | | | | | | | | |  | | | | | | | | | | | | | | | |  | | | | | | | | | | | | | | | | | | |  | | | | | | | | | | | | | | | | | | |  | | | | | | | | | | |  |
|  | | | | | | |  | | | | | | |  | | | | | | | | | | | | | | | | |  | | | | | | | |  | | | | | | | | | | | | | | | | | | |  | | | | | | | | | | |  | | | | | | | |  | | | | | | | | | | | | | |  | | | | | | | | | | | |  | | | | | | | | | | | | | | | | | | | | | | | | | |  | | | | | | | | | | | | | | | |  | | | | | | | | | | | | | |  | | | | | | | | | | | | | | |  | | | | | | | | | | | | | | | |  | | | | | | | |  | | | | | | | | | | | | | | | |  | | | | | | | | | | | | | | | | | | | | | | | | | |  | | | | | | | | | | | |  | | | | | | | | | | | | | | | | | | |  | | | | | | | | | | | |  | | | | | | | | |  | | | | | | | | | | | | | | | | | | | | |  | | | | | | | | | | | | | | | | | | |  | | | | | | | | | | | |  | | | | | | | | | | | | |  | | | | | | | | | | | | |  | | | | | | | | | | | | | | | | | | | | | | |  | | | | | | | | | | | | | | | |  | | | | | | | | | | | | | | | | |  | | | | | | | | | | | | | | | |  | | | | | | | | | | | | | | |  |  |  |  |  |  |  |  |  |  |  |  |  |  |  |  |
| **Respondant´s name** | | | | | | | | | | | | | | | | | | | | | | | | | | | | | | | | | | | | | | | | | | | | | | | | | | | | | | | | | | | | | | | | | | | | | | | | | | | | | | | | | | |  | | | | | | | | | | | | |  | | | | | | | | | | | | | |  | | | | | | | | | | | |  | | | | | | | | | | | | | | | | |  | | | | | | | | | | | | | | | | |  | | | | | | | | | | | | | Respondant´s ID | | | | | | | | | | | | | | | | | | | | | | | | | | | | | | | | | | | | | |  | | | | | | | | | | | | |  | | | | | | | | | | | |  | | | | | | | | | | | | | | | | | | | | | | | |  | | | | | | | | | | |  | | | | | | | | | | | | | |  | | | | | | | | | | | | | | |  | | | | | | | | | | | | |  | | | | | | | | | | | | | | | | |  | | | | | | | | | | | | | | | | | |  | | | | | | | | | | | | |  | | | | | | | | | | | | | | | | | |  | | | | | | | | | | | | |  | | | | | | | | | | | | | | | | | | |  | | | | | | | | | | | | | | | |  | | | | | | | | | | | | | | | | | | |  | | | | | | | | | | | | | | | | | | |  | | | | | | | | | | |  |
|  | | | | | | |  | | | | | | |  | | | | | | | | | | | | | | | | |  | | | | | | | |  | | | | | | | | | | | | | | | | | | |  | | | | | | | | | | |  | | | | | | | |  | | | | | | | | | | | | | |  | | | | | | | | | | | |  | | | | | | | | | | | | | | | | | | | | | | | | | |  | | | | | | | | | | | | | | | |  | | | | | | | | | | | | | |  | | | | | | | | | | | | | | |  | | | | | | | | | | | | | | | |  | | | | | | | |  | | | | | | | | | | | | | | | |  | | | | | | | | | | | | | | | | | | | | | | | | | |  | | | | | | | | | | | |  | | | | | | | | | | | | | | | | | | |  | | | | | | | | | | | |  | | | | | | | | |  | | | | | | | | | | | | | | | | | | | | |  | | | | | | | | | | | | | | | | | | |  | | | | | | | | | | | |  | | | | | | | | | | | | |  | | | | | | | | | | | | |  | | | | | | | | | | | | | | | | | | | | | | |  | | | | | | | | | | | | | | | |  | | | | | | | | | | | | | | | | |  | | | | | | | | | | | | | | | |  | | | | | | | | | | | | | | |  |  |  |  |  |  |  |  |  |  |  |  |  |  |  |  |
|  | | | | | | |  | | | | | | |  | | | | | | | | | | | | | | | | |  | | | | | | | |  | | | | | | | | | | | | | | | | | | |  | | | | | | | | | | |  | | | | | | | |  | | | | | | | | | | | | | |  | | | | | | | | | | | |  | | | | | | | | | | | | | | | | | | | | | | | | | |  | | | | | | | | | | | | | | | |  | | | | | | | | | | | | | |  | | | | | | | | | | | | | | |  | | | | | | | | | | | | | | | |  | | | | | | | |  | | | | | | | | | | | | | | | |  | | | | | | | | | | | | | | | | | | | | | | | | | |  | | | | | | | | | | | |  | | | | | | | | | | | | | | | | | | |  | | | | | | | | | | | |  | | | | | | | | |  | | | | | | | | | | | | | | | | | | | | |  | | | | | | | | | | | | | | | | | | |  | | | | | | | | | | | |  | | | | | | | | | | | | |  | | | | | | | | | | | | |  | | | | | | | | | | | | | | | | | | | | | | |  | | | | | | | | | | | | | | | |  | | | | | | | | | | | | | | | | |  | | | | | | | | | | | | | | | |  | | | | | | | | | | | | | | |  |  |  |  |  |  |  |  |  |  |  |  |  |  |  |  |
| **Investigator** | | | | | | | | | | | | | | | | | | | |  | | | | | | | | | | | | | | | | | |  | | | | | | | | | |  | | | | | | | | | | | | **Supervisor** | | | | | | | | | | | | | | | | | | | | | | | | | | | | | | | | | | | | | | | | | | | | | | | | | | |  | | | | | | | |  | | | | | | | | | | | | | | | |  | | | | | | | | | | | | | | | **Data entry clerk** | | | | | | | | | | | | | | | | | | | | | | | | | | | | | | | | | | | | | | | | | | | | | | | | | | | | | | | | | | |  | | | | | | | | | | | | | | |  | | | | | | | | | | | | | | | | | | | |  | | | | | | | | | | | | | | | | **Data entry supervisor** | | | | | | | | | | | | | | | | | | | | | | | | | | | | | | | | | | | | | | | | | | | | | | | | | | | | | | | | | | | | | | | | | | | | | | | | | | | | | | | |  | | | | | | | | | | | | | |  | | | | | | | | | | | |  | | | | | | | | | | | | | | | | | | |  | | | | | | | | | | | | | | | | | |  | | | | | | | | | | | | | | | | |  | | | | | | | | | | | | | | | |  | | | | | | | | | | | | | | | | | | | | | | | | | | | | | | | | | | | |  |
| code | | | | | | | | |  | | | | | | | | | | |  | | | | | | | | | | | | | | | | | |  | | | | | | | | | |  | | | | | | | | | | | | code | | | | | | | | | | | | | | | | | | | | | | | | | | | | | | |  | | | | | | | | | | | | | |  | | | | | | | | | | |  | | | | | | | | | | | | | | | | | |  | | | | | | | | | | | | | | | code | | | | | | | | | | | | | | | | | | | | | | | | | | | | |  | | | | | | | | |  | | | | | | | | | | | | | | | | | | | | |  | | | | | | | | | | | | |  | | | | | | | | | | | | | | | | | | | | | |  | | | | | | | | | | | | | | code | | | | | | | | | | | | | | | | | | | | | | | | | | |  | | | | | | | | | | | | |  | | | | | | | | | | | | | |  | | | | | | | | | | | | | | | |  | | | | | | | | | | | |  | | | | | | | | | | | | | |  | | | | | | | | | | |  | | | | | | | | | | | | | | | | | | | |  | | | | | | | | | | | | | | | | |  | | | | | | | | | | | | | | | | | |  | | | | | | | | | | | | | | | |  | | | | | | | | | | | | | | |  | | | | | | | | | | | | | | | |  | | | | |  |  |
| date | | | | | | | | | |  | | | | | | | | | | |  | | | | | | | | | | | | | | | | | | | |  | | | | | | | | |  | | | | | | | | | | | | date | | | | | | | | | | | | | | | | | | | | | | | |  | | | | | | | | | | | | |  | | | | | | | | | | | | | | | | |  | | | | | | | | | |  | | | | | | | | | | | | | | | |  | | | | | | | | | | | | | | | | | | | | | | date | | | | | | | | | | | | | | | | | | | | | |  | | | | | | | | | | | | |  | | | | | | | | | | | | | | | | | | | | | | | | | |  | | | | | | | | | |  | | | | | | | | | | | | | | | | | | | | | | | | | |  | | | | | | | | | | | | date | | | | | | | | | | | | | | | | | | | | | | | | | | | |  | | | | | | | | | | | | | | | |  | | | | | | | | | | | | |  | | | | | | | | | | | | | | | | |  | | | | | | | | | | |  | | | | | | | | | | | | | | | | | |  | | | | | | | | | | | | |  | | | | | | | | | | | | | | | | | | |  | | | | | | | | | | | | | | |  | | | | | | | | | | | | | | | | |  | | | | | | | | | | | | | | | | | | |  | | | | | | | | | | | | |  |
| Signature | | | | | | | | | |  | | | | | | | | | | | | | | | | | | | | | | | |  | | | | | | |  | | | | | | | | |  | | | | | | | | | | | | Signature | | | | | | | | | | | | | | | | | | | | | | | | | | | | | | | | | | | | | | | | | |  | | | | | | | | | | | | | |  | | | | | | | | | | | |  | | | | | | | | | | | | | |  | | | | | | | | | | | | | | | | | | | | | Signature | | | | | | | | | | | | | | | | | | | | | | | | | | |  | | | | | | | | | | |  | | | | | | | | | | | | | | | | | | | | | | |  | | | | | | | | | | | |  | | | | | | | | | | | | | | | | | | | | | | | |  | | | | | | | | | | | | Signature | | | | | | | | | | | | | | | | | | | | | | | | | | | |  | | | | | | | | | | | | | | | |  | | | | | | | | | | | | | | |  | | | | | | | | | | | | | | |  | | | | | | | | | | | | |  | | | | | | | | | | | | | | | | | |  | | | | | | | | | | | | | | |  | | | | | | | | | | | | | | | | | | | |  | | | | | | | | | | | | | | |  | | | | | | | | | | | | | | |  | | | | | | | | | | | | | | | | | | |  | | | | | | | | |  |
|  | | | |  | | | | | | | | | | | |  | | | | | | | | | | | | | | | | |  | | | | | | |  | | | | | | | | | | | | | | | | | | |  | | | | | | | | | | | |  | | | | | | | | |  | | | | | | | | | | | | | |  | | | | | | | | | | | | | |  | | | | | | | | | | | | | | | | | | | | | | | | |  | | | | | | | | | | | | | | |  | | | | | | | | | | | | | | |  | | | | | | | | | | | | | |  | | | | | | | | |  | | | | | | | | | | | | | | | | |  | | | | | | | | | | | | | | |  | | | | | | | | | | | | | | | | | | | | | | | | |  | | | | | | | | | | | | |  | | | | | | | | | | | | | | | | |  | | | | | | | | | | | | | |  | | | | | | | | | | | | |  | | | | | | | | | | | | | | |  | | | | | | | | | | | | | | | | | | | |  | | | | | | | | | | |  | | | | | | | | | | | | | |  | | | | | | | | | | | |  | | | | | | | | | | | | | | | | | | | | | | | | |  | | | | | | | | | | | | | | | | |  | | | | | | | | | | | | | | | | |  | | | | | | | | | | | | | | |  | | | | | | | | | | | | |  |  |  |  |  |  |  |  |  |  |  |  |  |  |
| **Survey characteristics** | | | | | | | | | | | | | | | | | | | | | | | | | | | | | | | | | | | | | | | | | | | | | | | | | | | | | | | | | | | | | | | | | | | | | | | | | | | | | | | | | | | | | | | | | | | | | | | | | | | | | | | | | | | | | | | | |  | | | | | | | | | | | |  | | | | | | | | | | | | | | | |  | | | | | | | | | | | | | | | | | | | |  | | | | | | | | | | |  | | | | | | | | | | | |  | | | | | | | | | | | |  | | | | | | | | | | | | | | | | | | | | | | | | | | |  | | | | | | | | | | |  | | | | | | | | | | | | | | | | | | | | | | | | | |  | | | | | | | | | | |  | | | | | | | | | | | | | | |  | | | | | | | | | | | | |  | | | | | | | | | | | | | | | | | |  | | | | | | | | | | | |  | | | | | | | | | | | | | | | | |  | | | | | | | | | | | | | |  | | | | | | | | | | | | | | | | | | |  | | | | | | | | | | | | |  | | | | | | | | | | | | | | | | | | | | |  | | | | | | | | | | | | | | | |  | | | | | | | | | | | | | | | |  | | | | | | | | | | | | | | | | | | | | |  | | | | | | |
|  | | | |  | | | | | | | | | | | |  | | | | | | | | | | | | | | | | |  | | | | | | |  | | | | | | | | | | | | | | | | | | |  | | | | | | | | | | | |  | | | | | | | | |  | | | | | | | | | | | | | |  | | | | | | | | | | | | | |  | | | | | | | | | | | | | | | | | | | | | | | | |  | | | | | | | | | | | | | | |  | | | | | | | | | | | | | | |  | | | | | | | | | | | | | |  | | | | | | | | |  | | | | | | | | | | | | | | | | |  | | | | | | | | | | | | | | |  | | | | | | | | | | | | | | | | | | | | | | | | |  | | | | | | | | | | | | |  | | | | | | | | | | | | | | | | |  | | | | | | | | | | | | | |  | | | | | | | | | | | | |  | | | | | | | | | | | | | | |  | | | | | | | | | | | | | | | | | | | |  | | | | | | | | | | |  | | | | | | | | | | | | | |  | | | | | | | | | | | |  | | | | | | | | | | | | | | | | | | | | | | | | |  | | | | | | | | | | | | | | | | |  | | | | | | | | | | | | | | | | |  | | | | | | | | | | | | | | |  | | | | | | | | | | | | |  |  |  |  |  |  |  |  |  |  |  |  |  |  |
| **Name, given name** | | | | | | | | | | | | | | | | | | | | | | | | | | | | | | | | | | | | | | | | | | | | | | |  | | | | | | | | **Date of birth** | | | | | | | | | | | | | | | | | | | | | | | | | | | | | | | | | | | | | | | | | | | | | | | | | | | | | | | | | | | | | | | | | | | | | |  | | | | | | | | | | | | | | | |  | | | | | | | | | | | | | | | | | | | |  | | | | | | | | | | |  | | | | | | | | | | | |  | | | | | | | | | | | |  | | | | | | | | | | | | | | | | | | | | | | | | | | |  | | | | | | | | | | |  | | | | | | | | | | | | | | | | | | | | | | | | | |  | | | | | | | | | | | **Age** | | | | | | | | | | | | | | |  | | | | | | | | | | | | |  | | | | | | | | | | | | | | | | | |  | | | | | | | | | | | |  | | | | | | | | | | | | | | | | |  | | | | | | | | | | | | | |  | | | | | | | | | | | | | | | | | | |  | | | | | | | | | | | | |  | | | | | | | | | | | | | | | | | | | | |  | | | | | | | | | | | | | | | |  | | | | | | | | | | | | | | | |  | | | | | | | | | | | | | | | | | | | | |  | | | | | | |
|  | | | |  | | | | | | | | | | | |  | | | | | | | | | | | | | | | | |  | | | | | | |  | | | | | | | | | | | | | | | | | | |  | | | | | | | | | | | |  | | | | | | | | |  | | | | | | | | | | | | | |  | | | | | | | | | | | | | |  | | | | | | | | | | | | | | | | | | | | | | | | |  | | | | | | | | | | | | | | |  | | | | | | | | | | | | | | |  | | | | | | | | | | | | | |  | | | | | | | | |  | | | | | | | | | | | | | | | | |  | | | | | | | | | | | | | | |  | | | | | | | | | | | | | | | | | | | | | | | | |  | | | | | | | | | | | | |  | | | | | | | | | | | | | | | | |  | | | | | | | | | | | | | |  | | | | | | | | | | | | |  | | | | | | | | | | | | | | |  | | | | | | | | | | | | | | | | | | | |  | | | | | | | | | | |  | | | | | | | | | | | | | |  | | | | | | | | | | | |  | | | | | | | | | | | | | | | | | | | | | | | | |  | | | | | | | | | | | | | | | | |  | | | | | | | | | | | | | | | | |  | | | | | | | | | | | | | | |  | | | | | | | | | | | | |  |  |  |  |  |  |  |  |  |  |  |  |  |  |
| **Educational level** | | | | | | | | | | | | | | | | | | | | | | | | | | | | | | | | | | | | | | | | | | | | | | | | | | | | | | |  | | | | | | | | | | | |  | | | | | | | | | | | | | | | | | |  | | | | | | | | | | | | |  | | | | | | | | | | | | | | |  | | | | | | | | | | | |  | | | | | | | | | | | | | | | | **Gender** | | | | | | | | | | | | | | | | | | | | 1.M | | | | | | | | | | |  | | | | | | | | | | | | 2.F | | | | | | | | | | | |  | | | | | | | | | | | | | | | | | | | | | | | | | | |  | | | | | | | | | | |  | | | | | | | | | | | | | | | | | | | | | | | | | |  | | | | | | | | | | |  | | | | | | | | | | | | | | |  | | | | | | | | | | | | |  | | | | | | | | | | | | | | | | | |  | | | | | | | | | | | |  | | | | | | | | | | | | | | | | |  | | | | | | | | | | | | | |  | | | | | | | | | | | | | | | | | | |  | | | | | | | | | | | | |  | | | | | | | | | | | | | | | | | | | | |  | | | | | | | | | | | | | | | |  | | | | | | | | | | | | | | | |  | | | | | | | | | | | | | | | | | | | | |  | | | | | | |
| 1- None | | | | | | | | | | | | | | | | | | | | | | | | | | | | | | | | |  | | | | | | |  | | | | | | | | | | | | | | | | | | |  | | | | | | | | | | | |  | | | | | | | | |  | | | | | | | | | | | | | |  | | | | | | | | | | | | | |  | | | | | | | | | | | | | | | | | | | | | | | | |  | | | | | | | | | | | | | | |  | | | | | | | | | | | | | | |  | | | | | | | | | | | | | |  | | | | | | | | |  | | | | | | | | | | | | | | | | |  | | | | | | | | | | | | | | |  | | | | | | | | | | | | | | | | | | | | | | | | |  | | | | | | | | | | | | |  | | | | | | | | | | | | | | | | |  | | | | | | | | | | | | | |  | | | | | | | | | | | | |  | | | | | | | | | | | | | | |  | | | | | | | | | | | | | | | | | | | |  | | | | | | | | | | |  | | | | | | | | | | | | | |  | | | | | | | | | | | |  | | | | | | | | | | | | | | | | | | | | | | | | |  | | | | | | | | | | | | | | | | |  | | | | | | | | | | | | | | | | |  | | | | | | | | | | | | | | |  | | | | | | | | | | | | |  |  |  |  |  |  |  |  |  |  |  |  |  |  |
| 2-Primary school | | | | | | | | | | | | | | | | | | | | | | | | | | | | | | | | |  | | | | | | |  | | | | | | | | | | | | | | | | | | |  | | | | | | | | | | | |  | | | | | | | | |  | | | | | | | | | | | | | |  | | | | | | | | | | | | | |  | | | | | | | | | | | | | | | | | | | | | | | | |  | | | | | | | | | | | | | | |  | | | | | | | | | | | | | | |  | | | | | | | | | | | | | |  | | | | | | | | |  | | | | | | | | | | | | | | | | |  | | | | | | | | | | | | | | |  | | | | | | | | | | | | | | | | | | | | | | | | |  | | | | | | | | | | | | |  | | | | | | | | | | | | | | | | |  | | | | | | | | | | | | | |  | | | | | | | | | | | | |  | | | | | | | | | | | | | | |  | | | | | | | | | | | | | | | | | | | |  | | | | | | | | | | |  | | | | | | | | | | | | | |  | | | | | | | | | | | |  | | | | | | | | | | | | | | | | | | | | | | | | |  | | | | | | | | | | | | | | | | |  | | | | | | | | | | | | | | | | |  | | | | | | | | | | | | | | |  | | | | | | | | | | | | |  |  |  |  |  |  |  |  |  |  |  |  |  |  |
| 3-Secondary school | | | | | | | | | | | | | | | | | | | | | | | | | | | | | | | | | | | | | | | |  | | | | | | | | | | | | | | | | | | |  | | | | | | | | | | | |  | | | | | | | | |  | | | | | | | | | | | | | |  | | | | | | | | | | | | | |  | | | | | | | | | | | | | | | | | | | | | | | | |  | | | | | | | | | | | | | | |  | | | | | | | | | | | | | | |  | | | | | | | | | | | | | |  | | | | | | | | |  | | | | | | | | | | | | | | | | |  | | | | | | | | | | | | | | |  | | | | | | | | | | | | | | | | | | | | | | | | |  | | | | | | | | | | | | |  | | | | | | | | | | | | | | | | |  | | | | | | | | | | | | | |  | | | | | | | | | | | | |  | | | | | | | | | | | | | | |  | | | | | | | | | | | | | | | | | | | |  | | | | | | | | | | |  | | | | | | | | | | | | | |  | | | | | | | | | | | |  | | | | | | | | | | | | | | | | | | | | | | | | |  | | | | | | | | | | | | | | | | |  | | | | | | | | | | | | | | | | |  | | | | | | | | | | | | | | |  | | | | | | | | | | | | |  |  |  |  |  |  |  |  |  |  |  |  |  |  |
| 4-Superior | | | | | | | | | | | | | | | | | | | | | | | | | | | | | | | | |  | | | | | | |  | | | | | | | | | | | | | | | | | | |  | | | | | | | | | | | |  | | | | | | | | |  | | | | | | | | | | | | | |  | | | | | | | | | | | | | |  | | | | | | | | | | | | | | | | | | | | | | | | |  | | | | | | | | | | | | | | |  | | | | | | | | | | | | | | |  | | | | | | | | | | | | | |  | | | | | | | | |  | | | | | | | | | | | | | | | | |  | | | | | | | | | | | | | | |  | | | | | | | | | | | | | | | | | | | | | | | | |  | | | | | | | | | | | | |  | | | | | | | | | | | | | | | | |  | | | | | | | | | | | | | |  | | | | | | | | | | | | |  | | | | | | | | | | | | | | |  | | | | | | | | | | | | | | | | | | | |  | | | | | | | | | | |  | | | | | | | | | | | | | |  | | | | | | | | | | | |  | | | | | | | | | | | | | | | | | | | | | | | | |  | | | | | | | | | | | | | | | | |  | | | | | | | | | | | | | | | | |  | | | | | | | | | | | | | | |  | | | | | | | | | | | | |  |  |  |  |  |  |  |  |  |  |  |  |  |  |
|  | | | |  | | | | | | | | | | | |  | | | | | | | | | | | | | | | | |  | | | | | | |  | | | | | | | | | | | | | | | | | | |  | | | | | | | | | | | |  | | | | | | | | |  | | | | | | | | | | | | | |  | | | | | | | | | | | | | |  | | | | | | | | | | | | | | | | | | | | | | | | |  | | | | | | | | | | | | | | |  | | | | | | | | | | | | | | |  | | | | | | | | | | | | | |  | | | | | | | | |  | | | | | | | | | | | | | | | | |  | | | | | | | | | | | | | | |  | | | | | | | | | | | | | | | | | | | | | | | | |  | | | | | | | | | | | | |  | | | | | | | | | | | | | | | | |  | | | | | | | | | | | | | |  | | | | | | | | | | | | |  | | | | | | | | | | | | | | |  | | | | | | | | | | | | | | | | | | | |  | | | | | | | | | | |  | | | | | | | | | | | | | |  | | | | | | | | | | | |  | | | | | | | | | | | | | | | | | | | | | | | | |  | | | | | | | | | | | | | | | | |  | | | | | | | | | | | | | | | | |  | | | | | | | | | | | | | | |  | | | | | | | | | | | | |  |  |  |  |  |  |  |  |  |  |  |  |  |  |
| **Number of household members** | | | | | | | | | | | | | | | | | | | | | | | | | | | | | | | | | | | | | | | | | | | | | | | | | | | | | | | | | | | | | | | | | | | | | | | | | | | | | | | | | | | | | | | | | | | | | | | | | |  | | | | | | | | | | | | | | |  | | | | | | | | | | | |  | | | | | | | | | | | | | | | |  | | | | | | | | | | | | | | | | | | | |  | | | | | | | | | | |  | | | | | | | | | | | |  | | | | | | | | | | | | **Number of children under 5 years of age in household** | | | | | | | | | | | | | | | | | | | | | | | | | | | | | | | | | | | | | | | | | | | | | | | | | | | | | | | | | | | | | | | | | | | | | | | | | | | | | | | | | | | | | | | | | | | | | | | | | | | | | | | | | | | | | | | | | | | | | | | | | | | | | | | | | | | | | | | | | | | | | | | | | | | | | |  | | | | | | | | | | | | | |  | | | | | | | | | | | | | | | | | | |  | | | | | | | | | | | | |  | | | | | | | | | | | | | | | | | | | | |  | | | | | | | | | | | | | | | |  | | | | | | | | | | | | | | | |  | | | | | | | | | | | | | | | | | | | | |  | | | | | | |
|  | | | |  | | | | | | | | | | | |  | | | | | | | | | | | | | | | | |  | | | | | | |  | | | | | | | | | | | | | | | | | | |  | | | | | | | | | | | |  | | | | | | | | |  | | | | | | | | | | | | | |  | | | | | | | | | | | | | |  | | | | | | | | | | | | | | | | | | | | | | | | |  | | | | | | | | | | | | | | |  | | | | | | | | | | | | | | |  | | | | | | | | | | | | | |  | | | | | | | | |  | | | | | | | | | | | | | | | | |  | | | | | | | | | | | | | | |  | | | | | | | | | | | | | | | | | | | | | | | | |  | | | | | | | | | | | | |  | | | | | | | | | | | | | | | | |  | | | | | | | | | | | | | |  | | | | | | |  | | | | | | | | | | | | | | | | | | | | |  | | | | | | | | | | | | | | | | | | | |  | | | | | | | | | | |  | | | | | | | | | | | | | |  | | | | | | | | | | | |  | | | | | | | | | | | | | | | | | | | | | | | | |  | | | | | | | | | | | | | | | | |  | | | | | | | | | | | | | | | | |  | | | | | | | | | | | | | | |  | | | | | | | | | | | | |  |  |  |  |  |  |  |  |  |  |  |  |  |  |
| The household has the following items / goods | | | | | | | | | | | | | | | | | | | | | | | | | | | | | | | | | | | | | | | | | | | | | | | | | | | | | | | | | | | | | | | | | | | | | | | | | | | | | | | | | | | | | | | | | | | | | | | | | | | | | | | | | | | | | | | | | | | | | | | | | | | | | | | | | | | | | | | | | | | | | | | | | | | | | | | | | | | | | | | | |  | | | | | | | | | | |  | | | | | | | | | | | |  | | | | | | | | | | | |  | | | | | | | | | | | | | | | | | | | | | | | | | | |  | | | | | | | | | | |  | | | | | | | | | | | | | | | | | | | | | | | | | |  | | | | | | | | | | |  | | | | | | | | | | | | | | |  | | | | | | | | | | | | |  | | | | | | | | | | | |  | | | | | | | | | | | | | | | | | |  | | | | | | | | | | | | | | | | |  | | | | | | | | | | | | | |  | | | | | | | | | | | | | | | | | | |  | | | | | | | | | | | | |  | | | | | | | | | | | | | | | | | | | | |  | | | | | | | | | | | | | | | |  | | | | | | | | | | | | | | | |  | | | | | | | | | | | | | | | | | | | | |  | | | | | | |
| 1-Bicycle | | | | | | | | | | | | | | | | | |  | | | | | | | |  | | | | | | | | | | | | | | | | | | | | |  | | | | | | | | 1.yes | | | | | | | | | | | | | | | | | |  | | | | | | | | | | | | 2.no | | | | | | | | | | | | | | | | | | | | | | | | | | | | | | |  | | | | | | | | | Number | | | | | | | | | | | | | | | | | | | | | | | | | | | | | | | | | | | |  | | | | | | | | | | |  | | | | | | | | | | | |  | | | | | | | | | | | |  | | | | | | | | | | | | | | | | | | | | | | | | | | | 11.Water connection | | | | | | | | | | | | | | | | | | | | | | | | | | | | | | | | | | | | | | | | | | | | | | | | | | | | | | | | | | | | | | | | | | | | | | | | | | | |  | | | | | | | | | | | | 1. yes | | | | | | | | | | | | | | | | | |  | | | | | | | | | | | | | | | | | 2.no | | | | | | | | | | | | | | | | | | | | | | | | | | | | | | | | | Number | | | | | | | | | | | | | | | | | | | | | | | | | | | | | | | | | |  | | | | | | | | | | | | | | | |  | | | | | | | | | | | | | | | |  | | | | | | | | | | | | | | | | | | | | |  | | | | | | |
| 2- Small Motorbike | | | | | | | | | | | | | | | | | | | | | | | | | | | | | | | | | | | | | | | | | | | | | | |  | | | | | | | | 1. yes | | | | | | | | | | | | | | | | | |  | | | | | | | | | | | | 2.no | | | | | | | | | | | | | | | | | | | | | | | | | | | | | | |  | | | | | | | | | Number | | | | | | | | | | | | | | | | | | | | | | | | | | | | | | | | | | | |  | | | | | | | | | | |  | | | | | | | | | | | |  | | | | | | | | | | | |  | | | | | | | | | | | | | | | | | | | | | | | | | | | 12.Poultry | | | | | | | | | | | | | | | | | | | | | | | | | | | | | | | | | | | | |  | | | | | | | | | | |  | | | | | | | | | | | | | | |  | | | | | | | | | | | | |  | | | | | | | | | | | | 1. yes | | | | | | | | | | | | | | | | | |  | | | | | | | | | | | | | | | | | 2.no | | | | | | | | | | | | | | | | | | | | | | | | | | | | | | | | | Number | | | | | | | | | | | | | | | | | | | | | | | | | | | | | | | | | |  | | | | | | | | | | | | | | | |  | | | | | | | | | | | | | | | |  | | | | | | | | | | | | | | | | | | | | |  | | | | | | |
| 3-Motorbike | | | | | | | | | | | | | | | | | | |  | | | | | | | |  | | | | | | | | | | | | | | | | | | | |  | | | | | | | | 1. yes | | | | | | | | | | | | | | | | |  | | | | | | | | | | | | | 2.no | | | | | | | | | | | | | | | | | | | | | | | | | | | | |  | | | | | | | | | Number | | | | | | | | | | | | | | | | | | | | | | | | | | | | | | | | | | |  | | | | | | | | | | | |  | | | | | | | | | | | |  | | | | | | | | | | | |  | | | | | | | | | | | | | | | | | | | | | | | | | | | | | 13.Sheep | | | | | | | | | | | | | | | | | | | | | | | | | | | | | | | | | | |  | | | | | | | | | | |  | | | | | | | | | | | | | | | |  | | | | | | | | | | | | |  | | | | | | | | | | | | 1. yes | | | | | | | | | | | | | | | | | | |  | | | | | | | | | | | | | | | | | 2.no | | | | | | | | | | | | | | | | | | | | | | | | | | | | | | | Number | | | | | | | | | | | | | | | | | | | | | | | | | | | | | | | | | |  | | | | | | | | | | | | | | | |  | | | | | | | | | | | | | | | |  | | | | | | | | | | | | | | | | | | | | |  | | | | | | | |  |
| 4-Telephone (Landline) | | | | | | | | | | | | | | | | | | | | | | | | | | | | | | | | | | | | | | | | | | | | | | |  | | | | | | | | 1. yes | | | | | | | | | | | | | | | | |  | | | | | | | | | | | | | 2.no | | | | | | | | | | | | | | | | | | | | | | | | | | | | |  | | | | | | | | | Number | | | | | | | | | | | | | | | | | | | | | | | | | | | | | | | | | | |  | | | | | | | | | | | |  | | | | | | | | | | | |  | | | | | | | | | | | |  | | | | | | | | | | | | | | | | | | | | | | | | | | | | | 14.Goats | | | | | | | | | | | | | | | | | | | | | | | | | | | | | | | | | | |  | | | | | | | | | | |  | | | | | | | | | | | | | | | |  | | | | | | | | | | | | |  | | | | | | | | | | | | 1. yes | | | | | | | | | | | | | | | | | | |  | | | | | | | | | | | | | | | | | 2.no | | | | | | | | | | | | | | | | | | | | | | | | | | | | | | | Number | | | | | | | | | | | | | | | | | | | | | | | | | | | | | | | | | |  | | | | | | | | | | | | | | | |  | | | | | | | | | | | | | | | |  | | | | | | | | | | | | | | | | | | | | |  | | | | | | | |  |
| 5-Telephone (Mobile) | | | | | | | | | | | | | | | | | | | | | | | | | | | | | | | | | | | | | | | | | | | | | | |  | | | | | | | | 1. yes | | | | | | | | | | | | | | | | |  | | | | | | | | | | | | | 2.no | | | | | | | | | | | | | | | | | | | | | | | | | | | | |  | | | | | | | | | Number | | | | | | | | | | | | | | | | | | | | | | | | | | | | | | | | | | |  | | | | | | | | | | | |  | | | | | | | | | | | |  | | | | | | | | | | | |  | | | | | | | | | | | | | | | | | | | | | | | | | | | | | 15.Cattle | | | | | | | | | | | | | | | | | | | | | | | | | | | | | | | | | | |  | | | | | | | | | | |  | | | | | | | | | | | | | | | |  | | | | | | | | | | | | |  | | | | | | | | | | | | 1. yes | | | | | | | | | | | | | | | | | | |  | | | | | | | | | | | | | | | | | 2.no | | | | | | | | | | | | | | | | | | | | | | | | | | | | | | | Number | | | | | | | | | | | | | | | | | | | | | | | | | | | | | | | | | |  | | | | | | | | | | | | | | | |  | | | | | | | | | | | | | | | |  | | | | | | | | | | | | | | | | | | | | |  | | | | | | | |  |
| 6-Mosquito nets | | | | | | | | | | | | | | | | | | | | | | | | | | | | | | | | | | | | | | | | | | | | | | |  | | | | | | | | 1. yes | | | | | | | | | | | | | | | | |  | | | | | | | | | | | | | 2.no | | | | | | | | | | | | | | | | | | | | | | | | | | | | |  | | | | | | | | | Number | | | | | | | | | | | | | | | | | | | | | | | | | | | | | | | | | | |  | | | | | | | | | | | |  | | | | | | | | | | | |  | | | | | | | | | | | |  | | | | | | | | | | | | | | | | | | | | | | | | | | | | | 16.Horses | | | | | | | | | | | | | | | | | | | | | | | | | | | | | | | | | | |  | | | | | | | | | | |  | | | | | | | | | | | | | | | |  | | | | | | | | | | | | |  | | | | | | | | | | | | 1. yes | | | | | | | | | | | | | | | | | | |  | | | | | | | | | | | | | | | | | 2.no | | | | | | | | | | | | | | | | | | | | | | | | | | | | | | | Number | | | | | | | | | | | | | | | | | | | | | | | | | | | | | | | | | |  | | | | | | | | | | | | | | | |  | | | | | | | | | | | | | | | |  | | | | | | | | | | | | | | | | | | | | |  | | | | | | | |  |
| 7-TV | | | | | | | | | | | | | | | | | | | | | | | | | | | | | | | | | | | | | | | | | | | | | | |  | | | | | | | | 1. yes | | | | | | | | | | | | | | | | |  | | | | | | | | | | | | | 2.no | | | | | | | | | | | | | | | | | | | | | | | | | | | | |  | | | | | | | | | Number | | | | | | | | | | | | | | | | | | | | | | | | | | | | | | | | | | |  | | | | | | | | | | | |  | | | | | | | | | | | |  | | | | | | | | | | | |  | | | | | | | | | | | | | | | | | | | | | | | | | | | | | 17.Donkeys | | | | | | | | | | | | | | | | | | | | | | | | | | | | | | | | | | |  | | | | | | | | | | |  | | | | | | | | | | | | | | | |  | | | | | | | | | | | | |  | | | | | | | | | | | | 1. yes | | | | | | | | | | | | | | | | | | |  | | | | | | | | | | | | | | | | | 2.no | | | | | | | | | | | | | | | | | | | | | | | | | | | | | | | Number | | | | | | | | | | | | | | | | | | | | | | | | | | | | | | | | | |  | | | | | | | | | | | | | | | |  | | | | | | | | | | | | | | | |  | | | | | | | | | | | | | | | | | | | | |  | | | | | | | |  |
| 8-Land parcels | | | | | | | | | | | | | | | | | | | | | | | | | | |  | | | | | | | | | | | | | | | | | | | |  | | | | | | | | 1. yes | | | | | | | | | | | | | | | | |  | | | | | | | | | | | | | 2.no | | | | | | | | | | | | | | | | | | | | | | | | | | | | |  | | | | | | | | | Number M2 | | | | | | | | | | | | | | | | | | | | | | | | | | | | | | | | | | |  | | | | | | | | | | | |  | | | | | | | | | | | |  | | | | | | | | | | | |  | | | | | | | | | | | | | | | | | | | | | | | | | | | | | 18.Used rooms | | | | | | | | | | | | | | | | | | | | | | | | | | | | | | | | | | | | | | | | | | | | | | | | | | | | | | | | | | | | | | | | | | | | | | | | | | |  | | | | | | | | | | | | 1. yes | | | | | | | | | | | | | | | | | | |  | | | | | | | | | | | | | | | | | 2.no | | | | | | | | | | | | | | | | | | | | | | | | | | | | | | | Number | | | | | | | | | | | | | | | | | | | | | | | | | | | | | | | | | |  | | | | | | | | | | | | | | | |  | | | | | | | | | | | | | | | |  | | | | | | | | | | | | | | | | | | | | |  | | | | | | | |  |
| 9-Arable land | | | | | | | | | | | | | | | | | | | | | | | | | | | | | | | | | | | | | | | | | | | | | | |  | | | | | | | | 1. yes | | | | | | | | | | | | | | | | |  | | | | | | | | | | | | | 2.no | | | | | | | | | | | | | | | | | | | | | | | | | | | | |  | | | | | | | | | Number hectares | | | | | | | | | | | | | | | | | | | | | | | | | | | | | | | | | | |  | | | | | | | | | | | |  | | | | | | | | | | | |  | | | | | | | | | | | |  | | | | | | | | | | | | | | | | | | | | | | | | | | | | | 19.Electricity | | | | | | | | | | | | | | | | | | | | | | | | | | | | | | | | | | | | | | | | | | | | | | | | | | | | | | | | | | | | | | | | | | | | | | | | | | |  | | | | | | | | | | | | yes | | | | | | | | | | | | | | | | | | |  | | | | | | | | | | | | | | | | | 2.no | | | | | | | | | | | | | | | | | | | | | | | | | | | | | | | Number | | | | | | | | | | | | | | | | | | | | | | | | | | | | | | | | | |  | | | | | | | | | | | | | | | |  | | | | | | | | | | | | | | | |  | | | | | | | | | | | | | | | | | | | | |  | | | | | | | |  |
| 10-Radio | | | | | | | | | | | | | | | | | | | | | | | | | | |  | | | | | | | | | | | | | | | | | | | |  | | | | | | | | 1. yes | | | | | | | | | | | | | | | | |  | | | | | | | | | | | | | 2.no | | | | | | | | | | | | | | | | | | | | | | | | | | | | |  | | | | | | | | | Number | | | | | | | | | | | | | | | | | | | | | | | | | | | | | | | | | | |  | | | | | | | | | | | |  | | | | | | | | | | | |  | | | | | | | | | | | |  | | | | | | | | | | | | | | | | | | | | | | | | | | | | | 20.Solar panel | | | | | | | | | | | | | | | | | | | | | | | | | | | | | | | | | | | | | | | | | | | | | | | | | | | | | | | | | | | | | |  | | | | | | | | | | | | |  | | | | | | | | | | | | yes | | | | | | | | | | | | | | | | | | |  | | | | | | | | | | | | | | | | | 2.no | | | | | | | | | | | | | | | | | | | | | | | | | | | | | | | Number | | | | | | | | | | | | | | | | | | | | | | | | | | | | | | | | | |  | | | | | | | | | | | | | | | |  | | | | | | | | | | | | | | | |  | | | | | | | | | | | | | | | | | | | | |  | | | | | | | |  |
|  | | | | | |  | | | | | | | | | |  | | | | | | | | | | | | | | | | |  | | | | | | |  | | | | | | | | | | | | | | | | | | |  | | | | | | | | | | | |  | | | | | | | | |  | | | | | | | | | | | | | | |  | | | | | | | | | | |  | | | | | | | | | | | | | | | | | | | | | | | | |  | | | | | | | | | | | | | | |  | | | | | | | | | | | | | | |  | | | | | | | | | | | | | |  | | | | | | | | |  | | | | | | | | | | | | | | | | |  | | | | | | | | | | | | | | |  | | | | | | | | | | | | | | | | | | | | | | | | |  | | | | | | | | | | | | |  | | | | | | | | | | | | | | | | | |  | | | | | | | | | | | | | |  | | | | | | |  | | | | | | | | | | | | | | | | | | | | | |  | | | | | | | | | | | | | | | | | | | |  | | | | | | | | | | |  | | | | | | | | | | | | | |  | | | | | | | | | | | |  | | | | | | | | | | | | | | | | | | | | | | | |  | | | | | | | | | | | | | | | | |  | | | | | | | | | | | | | | | | |  | | | | | | | | | | | | | | |  | | | | | | | | | | | | |  |  |  |  |  |  |  |  |  |  |  |  |  |  |  |
| **General knowledge on malaria and malaria prevention** | | | | | | | | | | | | | | | | | | | | | | | | | | | | | | | | | | | | | | | | | | | | | | | | | | | | | | | | | | | | | | | | | | | | | | | | | | | | | | | | | | | | | | | | | | | | | | | | | | | | | | | | | | | | | | | | | | | | | | | | | | | | | | | | | | | | | | | | | | | | | | | | | | | | | | | | | | | | | | | | | | | | | | | | | | | | | | | | | | | | | | | | | | | | | | | | | | | | | | | | | | | | | | | | | | | | | | | | | | | | | | | | | | | | | | | | | | | | | | | | | | | | | | | | | | | | | | | | | | | | | | | | | | | | | | | | | | |  | | | | | | | | | | | |  | | | | | | | | | | | | |  | | | | | | | | | | | | | | | | | | |  | | | | | | | | | | | | | | | | | |  | | | | | | | | | | | | | | |  | | | | | | | | | | | | | | |  | | | | | | | | | | | | | | | |  | | | | | | | | | | | | | | | | | | |  | | | | | | | | | | | | | | | | | | |  | | | | | | | | | | | | | | | |  | | | | | | | | | | | | | | | | |  | | | | | | | | | | | | | | | | | |  |
|  | | | | | | |  | | | | | | | | | |  | | | | | | | |  | | | | | | | | | | | | | | | |  | | | | | | |  | | | | | | | | | | | |  | | | | | | | | | | | |  | | | | | | | | | | | | | |  | | | | | | | | | | |  | | | | | | | | | | | | | | |  | | | | | | | | | | | | | | | | | | | |  | | | | | | | | | | | | | | |  | | | | | | | | | | | | | | |  | | | | | | | | | | | | | |  | | | | | | | | | | | | | |  | | | | | | | | |  | | | | | | | | | | | | | | |  | | | | | | | | | | | | | | | | | | | | |  | | | | | | | | | | |  | | | | | | | | | | | | | | | | | | |  | | | | | | | | | | | |  | | | | | | | | |  | | | | | | | | | | | | | | | | | | | |  | | | | | | | | | | | | | | | |  | | | | | | | | | | | | | |  | | | | | | | | | | | | |  | | | | | | | | | | | |  | | | | | | | | | | | | | | | | | | | |  | | | | | | | | | | | | | | | | | |  | | | | | | | | | | | | | | | | |  | | | | | | | | | | | | | | | |  | | | | | | | | | | | | | | | | | | | | | | | | | | | | | | | | | | |  | |  |  |  |  |
| **1- How is malaria transmitted ?** | | | | | | | | | | | | | | | | | | | | | | | | | | | | | | | | | | | | | | | | | | | | | | | | | | | | | | | | | | | | | | | | | | | | | | | | | | | | | | | | | | | | | | | | | | | | | | | | | | | | | | | | | | | | | | | |  | | | | | | | | | | | | | | | | | | | |  | | | | | | | | | | | | | | |  | | | | | | | | | | | | | | |  | | | | | | | | | | | | | |  | | | | | | | | | | | | | |  | | | | | | | | |  | | | | | | | | | | | | | | |  | | | | | | | | | | | | | | | | | | | | | **2- How do mosquitoes emerge ?** | | | | | | | | | | | | | | | | | | | | | | | | | | | | | | | | | | | | | | | | | | | | | | | | | | | | | | | | | | | | | | | | | | | | | | | | | | | | | | | | | | | | | | | | | | | | | | | | | | | | | | | | | | | | | | | | | | | | | | | | | | | | | | | | | | | | | | | | | | | | | | | | | | | | | | | | | | | | | | | | | | | | | | | | | | | | | | | | | | | | |  | | | | | | | | | | | | | | | |  | | | | | | | | | | | | | | | | | | | | | | | | | | | | | | | | | | |  | |  |  |  |  |
|  | | | | | | | 1- Mosquito bites | | | | | | | | | | | | | | | | | | | | | | | | | | | | | | | | | | | | | | | | | | | | | | | | | | | | | | | | | | | | | | | | |  | | | | | | | | | | | | | |  | | | | | | | | | | |  | | | | | | | | | | | | | | |  | | | | | | | | | | | | | | | | | | | |  | | | | | | | | | | | | | | |  | | | | | | | | | | | | | | |  | | | | | | | | | | | | | |  | | | | | | | | | | | | | |  | | | | | | | | |  | | | | | | | | | | | | | | |  | | | | | | | | | | | | | | | | | | | | |  | | | | | | | | | | | 1- Directly from egg to mosquito | | | | | | | | | | | | | | | | | | | | | | | | | | | | | | | | | | | | | | | | | | | | | | | | | | | | | | | | | | | | | | | | | | | | | | | | | | | | | | | | | | | | | | | | | | | | | | | | | | | | | | | | | | | | | | | | | | | | | | | | | | | | | | | | | | | | | | | | | | | | | | | | | | | | | | | | | | | | | | | | | | | | | | | | | | | | | | | | | | | | | | | | | | | | | | | | | | | | | | | | | | | | | | | | | | | | | | | | | | | | |  | |  |  |  |  |
|  | | | | | | | 2- Food | | | | | | | | | | | | | | | | | | | | | | | | | | | | | | | | | |  | | | | | | |  | | | | | | | | | | | |  | | | | | | | | | | | |  | | | | | | | | | | | | | |  | | | | | | | | | | |  | | | | | | | | | | | | | | |  | | | | | | | | | | | | | | | | | | | |  | | | | | | | | | | | | | | |  | | | | | | | | | | | | | | |  | | | | | | | | | | | | | |  | | | | | | | | | | | | | |  | | | | | | | | |  | | | | | | | | | | | | | | |  | | | | | | | | | | | | | | | | | | | | |  | | | | | | | | | | | 2- They go through different stages (egg, larval stages, adult mosquito) | | | | | | | | | | | | | | | | | | | | | | | | | | | | | | | | | | | | | | | | | | | | | | | | | | | | | | | | | | | | | | | | | | | | | | | | | | | | | | | | | | | | | | | | | | | | | | | | | | | | | | | | | | | | | | | | | | | | | | | | | | | | | | | | | | | | | | | | | | | | | | | | | | | | | | | | | | | | | | | | | | | | | | | | | | | | | | | | | | | | | | | | | | | | | | | | | | | | | | | | | | | | | | | | | | | | | | | | | | | | |  | |  |  |  |  |
|  | | | | | | | 3- Other ( specify …………………………………………) | | | | | | | | | | | | | | | | | | | | | | | | | | | | | | | | | | | | | | | | | | | | | | | | | | | | | | | | | | | | | | | | | | | | | | | | | | | | | | | | | | | | | | | | | | | | | | | | | | | | | | | | | | | | | | | | | | | | | | | | | | | | | | | | | | | | | | | | | | | |  | | | | | | | | | | | | | | |  | | | | | | | | | | | | | |  | | | | | | | | | | | | | |  | | | | | | | | |  | | | | | | | | | | | | | | |  | | | | | | | | | | | | | | | | | | | | |  | | | | | | | | | | | 3- Other (specify ……………………………………………………..) | | | | | | | | | | | | | | | | | | | | | | | | | | | | | | | | | | | | | | | | | | | | | | | | | | | | | | | | | | | | | | | | | | | | | | | | | | | | | | | | | | | | | | | | | | | | | | | | | | | | | | | | | | | | | | | | | | | | | | | | | | | | | | | | | | | | | | | | | | | | | | | | | | | | | | | | | | | | | | | | | | | | | | | | | | | | | | | | | | | | | | | | | | | | | | | | | | | | | | | | | | | | | | | | | | | | | | | | | | | | |  | |  |  |  |  |
|  | | | | | | | 4- Don´t know | | | | | | | | | | | | | | | | | | | | | | | | | | | | | | | | | | | | | | | | |  | | | | | | | | | | | |  | | | | | | | | | | | |  | | | | | | | | | | | | | |  | | | | | | | | | | |  | | | | | | | | | | | | | | |  | | | | | | | | | | | | | | | | | | | |  | | | | | | | | | | | | | | |  | | | | | | | | | | | | | | |  | | | | | | | | | | | | | |  | | | | | | | | | | | | | |  | | | | | | | | |  | | | | | | | | | | | | | | |  | | | | | | | | | | | | | | | | | | | | |  | | | | | | | | | | | 4- Don´t know | | | | | | | | | | | | | | | | | | | | | | | | | | | | | | | | | | | | | | | | | | | | | | | | | | | | | | | | | | | |  | | | | | | | | | | | | | | | |  | | | | | | | | | | | | | |  | | | | | | | | | | | | |  | | | | | | | | | | | |  | | | | | | | | | | | | | | | | | | | |  | | | | | | | | | | | | | | | | | |  | | | | | | | | | | | | | | | | |  | | | | | | | | | | | | | | | |  | | | | | | | | | | | | | | | | | | | | | | | | | | | | | | | | | | |  | |  |  |  |  |
|  | | | | | | |  | | | | | | | | | |  | | | | | | | |  | | | | | | | | | | | | | | | |  | | | | | | |  | | | | | | | | | | | |  | | | | | | | | | | | |  | | | | | | | | | | | | | |  | | | | | | | | | | |  | | | | | | | | | | | | | | |  | | | | | | | | | | | | | | | | | | | |  | | | | | | | | | | | | | | |  | | | | | | | | | | | | | | |  | | | | | | | | | | | | | |  | | | | | | | | | | | | | |  | | | | | | | | |  | | | | | | | | | | | | | | |  | | | | | | | | | | | | | | | | | | | | |  | | | | | | | | | | |  | | | | | | | | | | | | | | | | | | |  | | | | | | | | | | | |  | | | | | | | | |  | | | | | | | | | | | | | | | | | | | |  | | | | | | | | | | | | | | | |  | | | | | | | | | | | | | |  | | | | | | | | | | | | |  | | | | | | | | | | | |  | | | | | | | | | | | | | | | | | | | |  | | | | | | | | | | | | | | | | | |  | | | | | | | | | | | | | | | | |  | | | | | | | | | | | | | | | |  | | | | | | | | | | | | | | | | | | | | | | | | | | | | | | | | | | |  | |  |  |  |  |
| **3- What do you think are factors that favor mosquito development ?** | | | | | | | | | | | | | | | | | | | | | | | | | | | | | | | | | | | | | | | | | | | | | | | | | | | | | | | | | | | | | | | | | | | | | | | | | | | | | | | | | | | | | | | | | | | | | | | | | | | | | | | | | | | | | | | | | | | | | | | | | | | | | | | | | | | | | | | | | | | | | | | | | | | | | | | | | | | | | | | | | | | | | | | | | | | | | | | | | | | | | | | | | | | | | | | | | | | | | | | | | | | | | | | | | | | | | | | | | | | | | | | | | | | | | | | | | **4- What do you think where one can find mosquito larvae** | | | | | | | | | | | | | | | | | | | | | | | | | | | | | | | | | | | | | | | | | | | | | | | | | | | | | | | | | | | | | | | | | | | | | | | | | | | | | | | | | | | | | | | | | | | | | | | | | | | | | | | | | | | | | | | | | | | | | | | | | | | | | | | | | | | | | | | | | | | | | | | | | | | | | | | | | | | | | | | | | | | | | | | | | | | | | | | | | | | | | | | | | | | | | | | | | | | | | | | | | | | | | | | | | | | | | | | | | | | | | | | | | | | | | | | | | |  | |  |  |  |  |
|  | | | | | | | 1- Rainfall | | | | | | | | | | | | | | | | | | | | | | | | | | | | | | | | | | | | | | | | | | | | | | | | | | | | | | | | | | | | | | | | |  | | | | | | | | | | | | | |  | | | | | | | | | | |  | | | | | | | | | | | | | | |  | | | | | | | | | | | | | | | | | | | |  | | | | | | | | | | | | | | |  | | | | | | | | | | | | | | |  | | | | | | | | | | | | | |  | | | | | | | | | | | | | |  | | | | | | | | |  | | | | | | | | | | | | | | |  | | | | | | | | | | | | | | | | | | | | |  | | | | | | | | | | | 1- Stagnant waters | | | | | | | | | | | | | | | | | | | | | | | | | | | | | | | | | | | | | | | | | | | | | | | | | | | | | | | | | | | | | | | | | | | | | | | | | | | | | | | | | | | | | | | | | | | | | | | | | | | | | | |  | | | | | | | | | | | |  | | | | | | | | | | | | | | | | | | | |  | | | | | | | | | | | | | | | | | |  | | | | | | | | | | | | | | | | |  | | | | | | | | | | | | | | | |  | | | | | | | | | | | | | | | | | | | | | | | | | | | | | | | | | | |  | |  |  |  |  |
|  | | | | | | | 2- Stagnant waters | | | | | | | | | | | | | | | | | | | | | | | | | | | | | | | | | | | | | | | | | | | | | | | | | | | | | | | | | | | | | | | | |  | | | | | | | | | | | | | |  | | | | | | | | | | |  | | | | | | | | | | | | | | |  | | | | | | | | | | | | | | | | | | | |  | | | | | | | | | | | | | | |  | | | | | | | | | | | | | | |  | | | | | | | | | | | | | |  | | | | | | | | | | | | | |  | | | | | | | | |  | | | | | | | | | | | | | | |  | | | | | | | | | | | | | | | | | | | | |  | | | | | | | | | | | 2- In the forest | | | | | | | | | | | | | | | | | | | | | | | | | | | | | | | | | | | | | | | | | | | | | | | | | | | | | | | | | | | |  | | | | | | | | | | | | | | | |  | | | | | | | | | | | | | |  | | | | | | | | | | | | |  | | | | | | | | | | | |  | | | | | | | | | | | | | | | | | | | |  | | | | | | | | | | | | | | | | | |  | | | | | | | | | | | | | | | | |  | | | | | | | | | | | | | | | |  | | | | | | | | | | | | | | | | | | | | | | | | | | | | | | | | | | |  | |  |  |  |  |
|  | | | | | | | 3- Cleannes of village | | | | | | | | | | | | | | | | | | | | | | | | | | | | | | | | | | | | | | | | | | | | | | | | | | | | | | | | | | | | | | | | |  | | | | | | | | | | | | | |  | | | | | | | | | | |  | | | | | | | | | | | | | | |  | | | | | | | | | | | | | | | | | | | |  | | | | | | | | | | | | | | |  | | | | | | | | | | | | | | |  | | | | | | | | | | | | | |  | | | | | | | | | | | | | |  | | | | | | | | |  | | | | | | | | | | | | | | |  | | | | | | | | | | | | | | | | | | | | |  | | | | | | | | | | | 3- In the pit latrines / toilets | | | | | | | | | | | | | | | | | | | | | | | | | | | | | | | | | | | | | | | | | | | | | | | | | | | | | | | | | | | | | | | | | | | | | | | | | | | | | | | | | | | | | | | | | | | | | | | | | | | | | | |  | | | | | | | | | | | |  | | | | | | | | | | | | | | | | | | | |  | | | | | | | | | | | | | | | | | |  | | | | | | | | | | | | | | | | |  | | | | | | | | | | | | | | | |  | | | | | | | | | | | | | | | | | | | | | | | | | | | | | | | | | | |  | |  |  |  |  |
|  | | | | | | | 4- Humidity | | | | | | | | | | | | | | | | | | | | | | | | | | | | | | | | | | | | | | | | |  | | | | | | | | | | | |  | | | | | | | | | | | |  | | | | | | | | | | | | | |  | | | | | | | | | | |  | | | | | | | | | | | | | | |  | | | | | | | | | | | | | | | | | | | |  | | | | | | | | | | | | | | |  | | | | | | | | | | | | | | |  | | | | | | | | | | | | | |  | | | | | | | | | | | | | |  | | | | | | | | |  | | | | | | | | | | | | | | |  | | | | | | | | | | | | | | | | | | | | |  | | | | | | | | | | | 4- In the vegetation around houses | | | | | | | | | | | | | | | | | | | | | | | | | | | | | | | | | | | | | | | | | | | | | | | | | | | | | | | | | | | | | | | | | | | | | | | | | | | | | | | | | | | | | | | | | | | | | | | | | | | | | | | | | | | | | | | | | | | | | | | | | | | | | | | | | | | | | | | | | | | | | | | | | | | | | | | | | | | | | | | | | | | | | | | | | |  | | | | | | | | | | | | | | | |  | | | | | | | | | | | | | | | | | | | | | | | | | | | | | | | | | | |  | |  |  |  |  |
|  | | | | | | |  | | | | | | | | | |  | | | | | | | |  | | | | | | | | | | | | | | | |  | | | | | | |  | | | | | | | | | | | |  | | | | | | | | | | | |  | | | | | | | | | | | | | |  | | | | | | | | | | |  | | | | | | | | | | | | | | |  | | | | | | | | | | | | | | | | | | | |  | | | | | | | | | | | | | | |  | | | | | | | | | | | | | | |  | | | | | | | | | | | | | |  | | | | | | | | | | | | | |  | | | | | | | | |  | | | | | | | | | | | | | | |  | | | | | | | | | | | | | | | | | | | | |  | | | | | | | | | | | 5- Don´t know | | | | | | | | | | | | | | | | | | | | | | | | | | | | | | | | | | | | | | | | | | | | | | | | | | | | | | | | | | | |  | | | | | | | | | | | | | | | |  | | | | | | | | | | | | | |  | | | | | | | | | | | | |  | | | | | | | | | | | |  | | | | | | | | | | | | | | | | | | | |  | | | | | | | | | | | | | | | | | |  | | | | | | | | | | | | | | | | |  | | | | | | | | | | | | | | | |  | | | | | | | | | | | | | | | | | | | | | | | | | | | | | | | | | | |  | |  |  |  |  |
|  | | | | | | |  | | | | | | | | | |  | | | | | | | |  | | | | | | | | | | | | | | | |  | | | | | | |  | | | | | | | | | | | |  | | | | | | | | | | | |  | | | | | | | | | | | | | |  | | | | | | | | | | |  | | | | | | | | | | | | | | |  | | | | | | | | | | | | | | | | | | | |  | | | | | | | | | | | | | | |  | | | | | | | | | | | | | | |  | | | | | | | | | | | | | |  | | | | | | | | | | | | | |  | | | | | | | | |  | | | | | | | | | | | | | | |  | | | | | | | | | | | | | | | | | | | | |  | | | | | | | | | | |  | | | | | | | | | | | | | | | | | | |  | | | | | | | | | | | |  | | | | | | | | |  | | | | | | | | | | | | | | | | | | | |  | | | | | | | | | | | | | | | |  | | | | | | | | | | | | | |  | | | | | | | | | | | | |  | | | | | | | | | | | |  | | | | | | | | | | | | | | | | | | | |  | | | | | | | | | | | | | | | | | |  | | | | | | | | | | | | | | | | |  | | | | | | | | | | | | | | | |  | | | | | | | | | | | | | | | | | | | | | | | | | | | | | | | | | | |  | |  |  |  |  |
| **5- What can one do to avoid contracting malaria ?** | | | | | | | | | | | | | | | | | | | | | | | | | | | | | | | | | | | | | | | | | | | | | | | | | | | | | | | | | | | | | | | | | | | | | | | | | | | | | | | | | | | | | | | | | | | | | | | | | | | | | | | | | | | | | | | | | | | | | | | | | | | | | | | | | | | |  | | | | | | | | | | | | | | |  | | | | | | | | | | | | | | |  | | | | | | | | | | | | | |  | | | | | | | | | | | | | |  | | | | | | | | | **6- What do you do to protect yourself against malaria ?** | | | | | | | | | | | | | | | | | | | | | | | | | | | | | | | | | | | | | | | | | | | | | | | | | | | | | | | | | | | | | | | | | | | | | | | | | | | | | | | | | | | | | | | | | | | | | | | | | | | | | | | | | | | | | | | | | | | | | | | | | | | | | | | | | | | | | | | | | | | | | | | | | | | | | | | | | | | | | | | | | | | | | | | | | | | | | | | | | | | | | | | | | | | | | | | | | | | | | | | | | | | | | | | | | | | | | | | | | | | | | | | | | | | | | | | | |  | | | | | | | | | | | | | | | | | | | | | | | | | | | | | | | | | | |  | |  |  |  |  |
|  | | | | | | | 1- Sleep under a bed net | | | | | | | | | | | | | | | | | | | | | | | | | | | | | | | | | | | | | | | | | | | | | | | | | | | | | | | | | | | | | | | | | | | | | | | | | | | | | | | | | | | | | | | | | |  | | | | | | | | | | | | | | |  | | | | | | | | | | | | | | | | | | | |  | | | | | | | | | | | | | | |  | | | | | | | | | | | | | | |  | | | | | | | | | | | | | |  | | | | | | | | | | | | | |  | | | | | | | | |  | | | | | | | | | | | | | | |  | | | | | | | | | | | | | | | | | | | | |  | | | | | | | | | | | 1- Sleep under a bed net | | | | | | | | | | | | | | | | | | | | | | | | | | | | | | | | | | | | | | | | | | | | | | | | | | | | | | | | | | | | | | | | | | | | | | | | | | | | | | | | | | | | | | | | | | | | | | | | | | | | | | | | | | | | | | | | | | |  | | | | | | | | | | | | | | | | | | | |  | | | | | | | | | | | | | | | | | |  | | | | | | | | | | | | | | | | |  | | | | | | | | | | | | | | | |  | | | | | | | | | | | | | | | | | | | | | | | | | | | | | | | | | | |  | |  |  |  |  |
|  | | | | | | | 2- Take medication | | | | | | | | | | | | | | | | | | | | | | | | | | | | | | | | | | | | | | | | | | | | | | | | | | | | | | | | | | | | | | | | |  | | | | | | | | | | | | | |  | | | | | | | | | | |  | | | | | | | | | | | | | | |  | | | | | | | | | | | | | | | | | | | |  | | | | | | | | | | | | | | |  | | | | | | | | | | | | | | |  | | | | | | | | | | | | | |  | | | | | | | | | | | | | |  | | | | | | | | |  | | | | | | | | | | | | | | |  | | | | | | | | | | | | | | | | | | | | |  | | | | | | | | | | | 2- Take medication | | | | | | | | | | | | | | | | | | | | | | | | | | | | | | | | | | | | | | | | | | | | | | | | | | | | | | | | | | | | | | | | | | | | | | | | | | | | | | | | | | | | | | | | | | | | | | | | | | | | | | |  | | | | | | | | | | | |  | | | | | | | | | | | | | | | | | | | |  | | | | | | | | | | | | | | | | | |  | | | | | | | | | | | | | | | | |  | | | | | | | | | | | | | | | |  | | | | | | | | | | | | | | | | | | | | | | | | | | | | | | | | | | |  | |  |  |  |  |
|  | | | | | | | 3- Sanitize living conditions | | | | | | | | | | | | | | | | | | | | | | | | | | | | | | | | | | | | | | | | | | | | | | | | | | | | | | | | | | | | | | | | | | | | | | | | | | | | | | |  | | | | | | | | | | |  | | | | | | | | | | | | | | |  | | | | | | | | | | | | | | | | | | | |  | | | | | | | | | | | | | | |  | | | | | | | | | | | | | | |  | | | | | | | | | | | | | |  | | | | | | | | | | | | | |  | | | | | | | | |  | | | | | | | | | | | | | | |  | | | | | | | | | | | | | | | | | | | | |  | | | | | | | | | | | 3- Sanitize living conditions | | | | | | | | | | | | | | | | | | | | | | | | | | | | | | | | | | | | | | | | | | | | | | | | | | | | | | | | | | | | | | | | | | | | | | | | | | | | | | | | | | | | | | | | | | | | | | | | | | | | | | |  | | | | | | | | | | | |  | | | | | | | | | | | | | | | | | | | |  | | | | | | | | | | | | | | | | | |  | | | | | | | | | | | | | | | | |  | | | | | | | | | | | | | | | |  | | | | | | | | | | | | | | | | | | | | | | | | | | | | | | | | | | |  | |  |  |  |  |
|  | | | | | | | 4- Spray chemicals | | | | | | | | | | | | | | | | | | | | | | | | | | | | | | | | | |  | | | | | | |  | | | | | | | | | | | |  | | | | | | | | | | | |  | | | | | | | | | | | | | |  | | | | | | | | | | |  | | | | | | | | | | | | | | |  | | | | | | | | | | | | | | | | | | | |  | | | | | | | | | | | | | | |  | | | | | | | | | | | | | | |  | | | | | | | | | | | | | |  | | | | | | | | | | | | | |  | | | | | | | | |  | | | | | | | | | | | | | | |  | | | | | | | | | | | | | | | | | | | | |  | | | | | | | | | | | 4- Spray chemicals (inside the house) | | | | | | | | | | | | | | | | | | | | | | | | | | | | | | | | | | | | | | | | | | | | | | | | | | | | | | | | | | | | | | | | | | | | | | | | | | | | | | | | | | | | | | | | | | | | | | | | | | | | | | | | | | | | | | | | | | | | | | | | | | | | | | | | | | | | | | |  | | | | | | | | | | | | | | | | | |  | | | | | | | | | | | | | | | | |  | | | | | | | | | | | | | | | |  | | | | | | | | | | | | | | | | | | | | | | | | | | | | | | | | | | |  | |  |  |  |  |
|  | | | | | | | 5- Apply repulsives on skin and clothes | | | | | | | | | | | | | | | | | | | | | | | | | | | | | | | | | | | | | | | | | | | | | | | | | | | | | | | | | | | | | | | | | | | | | | | | | | | | | | | | | | | | | | | | | |  | | | | | | | | | | | | | | |  | | | | | | | | | | | | | | | | | | | |  | | | | | | | | | | | | | | |  | | | | | | | | | | | | | | |  | | | | | | | | | | | | | |  | | | | | | | | | | | | | |  | | | | | | | | |  | | | | | | | | | | | | | | |  | | | | | | | | | | | | | | | | | | | | |  | | | | | | | | | | | 5- Apply repulsives on skin and clothes | | | | | | | | | | | | | | | | | | | | | | | | | | | | | | | | | | | | | | | | | | | | | | | | | | | | | | | | | | | | | | | | | | | | | | | | | | | | | | | | | | | | | | | | | | | | | | | | | | | | | | | | | | | | | | | | | | |  | | | | | | | | | | | | | | | | | | | |  | | | | | | | | | | | | | | | | | |  | | | | | | | | | | | | | | | | |  | | | | | | | | | | | | | | | |  | | | | | | | | | | | | | | | | | | | | | | | | | | | | | | | | | | |  | |  |  |  |  |
|  | | | | | | | 6- Use mosquito coils | | | | | | | | | | | | | | | | | | | | | | | | | | | | | | | | | | | | | | | | | | | | | | | | | | | | | | | | | | | | | | | | |  | | | | | | | | | | | | | |  | | | | | | | | | | |  | | | | | | | | | | | | | | |  | | | | | | | | | | | | | | | | | | | |  | | | | | | | | | | | | | | |  | | | | | | | | | | | | | | |  | | | | | | | | | | | | | |  | | | | | | | | | | | | | |  | | | | | | | | |  | | | | | | | | | | | | | | |  | | | | | | | | | | | | | | | | | | | | |  | | | | | | | | | | | 6- Use mosquito coils | | | | | | | | | | | | | | | | | | | | | | | | | | | | | | | | | | | | | | | | | | | | | | | | | | | | | | | | | | | | | | | | | | | | | | | | | | | | | | | | | | | | | | | | | |  | | | | | | | | | | | | |  | | | | | | | | | | | |  | | | | | | | | | | | | | | | | | | | |  | | | | | | | | | | | | | | | | | |  | | | | | | | | | | | | | | | | |  | | | | | | | | | | | | | | | |  | | | | | | | | | | | | | | | | | | | | | | | | | | | | | | | | | | |  | |  |  |  |  |
|  | | | | | | | 7- Nothing | | | | | | | | | | | | | | | | | |  | | | | | | | | | | | | | | | |  | | | | | | |  | | | | | | | | | | | |  | | | | | | | | | | | |  | | | | | | | | | | | | | |  | | | | | | | | | | |  | | | | | | | | | | | | | | |  | | | | | | | | | | | | | | | | | | | |  | | | | | | | | | | | | | | |  | | | | | | | | | | | | | | |  | | | | | | | | | | | | | |  | | | | | | | | | | | | | |  | | | | | | | | |  | | | | | | | | | | | | | | |  | | | | | | | | | | | | | | | | | | | | |  | | | | | | | | | | | 7- Nothing | | | | | | | | | | | | | | | | | | | | | | | | | | | | | | |  | | | | | | | | |  | | | | | | | | | | | | | | | | | | | |  | | | | | | | | | | | | | | | |  | | | | | | | | | | | | | |  | | | | | | | | | | | | |  | | | | | | | | | | | |  | | | | | | | | | | | | | | | | | | | |  | | | | | | | | | | | | | | | | | |  | | | | | | | | | | | | | | | | |  | | | | | | | | | | | | | | | |  | | | | | | | | | | | | | | | | | | | | | | | | | | | | | | | | | | |  | |  |  |  |  |
|  | | | | | | | 8- Other (Specify …………………………………………..) | | | | | | | | | | | | | | | | | | | | | | | | | | | | | | | | | | | | | | | | | | | | | | | | | | | | | | | | | | | | | | | | | | | | | | | | | | | | | | | | | | | | | | | | | | | | | | | | | | | | | | | | | | | | | | | | | | | | | | | | | | | | | | | | | | | | | | | | | | | |  | | | | | | | | | | | | | | |  | | | | | | | | | | | | | |  | | | | | | | | | | | | | |  | | | | | | | | |  | | | | | | | | | | | | | | |  | | | | | | | | | | | | | | | | | | | | |  | | | | | | | | | | | 8- Other (Specify ……………………) | | | | | | | | | | | | | | | | | | | | | | | | | | | | | | | | | | | | | | | | | | | | | | | | | | | | | | | | | | | | | | | | | | | | | | | | | | | | | | | | | | | | | | | | | | | | | | | | | | | | | | | | | | | | | | | | | | | | | | | | | | | | | | | | | | | | | | | | | | | | | | | | | | | | | | | | | | | | | | | | | | | | | | | | | |  | | | | | | | | | | | | | | | |  | | | | | | | | | | | | | | | | | | | | | | | | | | | | | | | | | | |  | |  |  |  |  |
|  | | | | | | | 9- Don´t know | | | | | | | | | | | | | | | | | | | | | | | | | | | | | | | | | | | | | | | | |  | | | | | | | | | | | |  | | | | | | | | | | | |  | | | | | | | | | | | | | |  | | | | | | | | | | |  | | | | | | | | | | | | | | |  | | | | | | | | | | | | | | | | | | | |  | | | | | | | | | | | | | | |  | | | | | | | | | | | | | | |  | | | | | | | | | | | | | |  | | | | | | | | | | | | | |  | | | | | | | | |  | | | | | | | | | | | | | | |  | | | | | | | | | | | | | | | | | | | | |  | | | | | | | | | | | 9- Insecticide spraying outside the house | | | | | | | | | | | | | | | | | | | | | | | | | | | | | | | | | | | | | | | | | | | | | | | | | | | | | | | | | | | | | | | | | | | | | | | | | | | | | | | | | | | | | | | | | | | | | | | | | | | | | | | | | | | | | | | | | | | | | | | | | | | | | | | | | | | | | | | | | | | | | | | | | | | | | | | | | | | | | | | | | | | | | | | | | |  | | | | | | | | | | | | | | | |  | | | | | | | | | | | | | | | | | | | | | | | | | | | | | | | | | | |  | |  |  |  |  |
|  | | | | | | |  | | | | | | | | | |  | | | | | | | |  | | | | | | | | | | | | | | | |  | | | | | | |  | | | | | | | | | | | |  | | | | | | | | | | | |  | | | | | | | | | | | | | |  | | | | | | | | | | |  | | | | | | | | | | | | | | |  | | | | | | | | | | | | | | | | | | | |  | | | | | | | | | | | | | | |  | | | | | | | | | | | | | | |  | | | | | | | | | | | | | |  | | | | | | | | | | | | | |  | | | | | | | | |  | | | | | | | | | | | | | | |  | | | | | | | | | | | | | | | | | | | | |  | | | | | | | | | | | 10- Don´t know | | | | | | | | | | | | | | | | | | | | | | | | | | | | | | | | | | | | | | | | | | | | | | | | | | | | | | | | | | | |  | | | | | | | | | | | | | | | |  | | | | | | | | | | | | | |  | | | | | | | | | | | | |  | | | | | | | | | | | |  | | | | | | | | | | | | | | | | | | | |  | | | | | | | | | | | | | | | | | |  | | | | | | | | | | | | | | | | |  | | | | | | | | | | | | | | | |  | | | | | | | | | | | | | | | | | | | | | | | | | | | | | | | | | | |  | |  |  |  |  |
|  | | | | | | |  | | | | | | | | | |  | | | | | | | |  | | | | | | | | | | | | | | | |  | | | | | | |  | | | | | | | | | | | |  | | | | | | | | | | | |  | | | | | | | | | | | | | |  | | | | | | | | | | |  | | | | | | | | | | | | | | |  | | | | | | | | | | | | | | | | | | | |  | | | | | | | | | | | | | | |  | | | | | | | | | | | | | | |  | | | | | | | | | | | | | |  | | | | | | | | | | | | | |  | | | | | | | | |  | | | | | | | | | | | | | | |  | | | | | | | | | | | | | | | | | | | | |  | | | | | | | | | | |  | | | | | | | | | | | | | | | | | | |  | | | | | | | | | | | |  | | | | | | | | |  | | | | | | | | | | | | | | | | | | | |  | | | | | | | | | | | | | | | |  | | | | | | | | | | | | | |  | | | | | | | | | | | | |  | | | | | | | | | | | |  | | | | | | | | | | | | | | | | | | | |  | | | | | | | | | | | | | | | | | |  | | | | | | | | | | | | | | | | |  | | | | | | | | | | | | | | | |  | | | | | | | | | | | | | | | | | | | | | | | | | | | | | | | | | | |  | |  |  |  |  |
| **7- Do you use mosquito net in your household ?** | | | | | | | | | | | | | | | | | | | | | | | | | | | | | | | | | | | | | | | | | | | | | | | | | | | | | | | | | | | | | | | | | | | | | | | | | | | | | | | | | | | | | | | | | | | | | | | | | | | | | | | | | | | | | | | | | | | | | | | | | | | | | | | | | | | | | | | | | | | | | | | | | | | | | | | | | | | | | | | | | |  | | | | | | | | | | | | | |  | | | | | | | | | | | | | |  | | | | | | | | |  | | | | | | | | | | | | | | |  | | | | | | | | | | | | | | | | | | | | |  | | | | | | | | | | |  | | | | | | | | | | | | | | | | | | |  | | | | | | | | | | | |  | | | | | | | | |  | | | | | | | | | | | | | | | | | | | |  | | | | | | | | | | | | | | | |  | | | | | | | | | | | | | |  | | | | | | | | | | | | |  | | | | | | | | | | | |  | | | | | | | | | | | | | | | | | | | |  | | | | | | | | | | | | | | | | | |  | | | | | | | | | | | | | | | | |  | | | | | | | | | | | | | | | |  | | | | | | | | | | | | | | | | | | | | | | | | | | | | | | | | | | |  | |  |  |  |  |
|  | | | | | | | 1- Yes | | | | | | | | | | | | | | | | | |  | | | | | | | | | | | | | | | |  | | | | | | |  | | | | | | | | | | | |  | | | | | | | | | | | |  | | | | | | | | | | | | | |  | | | | | | | | | | |  | | | | | | | | | | | | | | |  | | | | | | | | | | | | | | | | | | | |  | | | | | | | | | | | | | | |  | | | | | | | | | | | | | | |  | | | | | | | | | | | | | |  | | | | | | | | | | | | | |  | | | | | | | | |  | | | | | | | | | | | | | | |  | | | | | | | | | | | | | | | | | | | | |  | | | | | | | | | | |  | | | | | | | | | | | | | | | | | | |  | | | | | | | | | | | |  | | | | | | | | |  | | | | | | | | | | | | | | | | | | | |  | | | | | | | | | | | | | | | |  | | | | | | | | | | | | | |  | | | | | | | | | | | | |  | | | | | | | | | | | |  | | | | | | | | | | | | | | | | | | | |  | | | | | | | | | | | | | | | | | |  | | | | | | | | | | | | | | | | |  | | | | | | | | | | | | | | | |  | | | | | | | | | | | | | | | | | | | | | | | | | | | | | | | | | | |  | |  |  |  |  |
|  | | | | | | | 2- No | | | | | | | | | | | | | | | | | |  | | | | | | | | | | | | | | | |  | | | | | | |  | | | | | | | | | | | |  | | | | | | | | | | | |  | | | | | | | | | | | | | |  | | | | | | | | | | |  | | | | | | | | | | | | | | |  | | | | | | | | | | | | | | | | | | | |  | | | | | | | | | | | | | | |  | | | | | | | | | | | | | | |  | | | | | | | | | | | | | |  | | | | | | | | | | | | | |  | | | | | | | | |  | | | | | | | | | | | | | | |  | | | | | | | | | | | | | | | | | | | | |  | | | | | | | | | | |  | | | | | | | | | | | | | | | | | | |  | | | | | | | | | | | |  | | | | | | | | |  | | | | | | | | | | | | | | | | | | | |  | | | | | | | | | | | | | | | |  | | | | | | | | | | | | | |  | | | | | | | | | | | | |  | | | | | | | | | | | |  | | | | | | | | | | | | | | | | | | | |  | | | | | | | | | | | | | | | | | |  | | | | | | | | | | | | | | | | |  | | | | | | | | | | | | | | | |  | | | | | | | | | | | | | | | | | | | | | | | | | | | | | | | | | | |  | |  |  |  |  |
|  | | | | | | | 3- Don´t know | | | | | | | | | | | | | | | | | | | | | | | | | | | | | | | | | | | | | | | | |  | | | | | | | | | | | |  | | | | | | | | | | | |  | | | | | | | | | | | | | |  | | | | | | | | | | |  | | | | | | | | | | | | | | |  | | | | | | | | | | | | | | | | | | | |  | | | | | | | | | | | | | | |  | | | | | | | | | | | | | | |  | | | | | | | | | | | | | |  | | | | | | | | | | | | | |  | | | | | | | | |  | | | | | | | | | | | | | | |  | | | | | | | | | | | | | | | | | | | | |  | | | | | | | | | | |  | | | | | | | | | | | | | | | | | | |  | | | | | | | | | | | |  | | | | | | | | |  | | | | | | | | | | | | | | | | | | | |  | | | | | | | | | | | | | | | |  | | | | | | | | | | | | | |  | | | | | | | | | | | | |  | | | | | | | | | | | |  | | | | | | | | | | | | | | | | | | | |  | | | | | | | | | | | | | | | | | |  | | | | | | | | | | | | | | | | |  | | | | | | | | | | | | | | | |  | | | | | | | | | | | | | | | | | | | | | | | | | | | | | | | | | | |  | |  |  |  |  |
|  | | | | | | |  | | | | | | | | | |  | | | | | | | |  | | | | | | | | | | | | | | | |  | | | | | | |  | | | | | | | | | | | |  | | | | | | | | | | | |  | | | | | | | | | | | | | |  | | | | | | | | | | |  | | | | | | | | | | | | | | |  | | | | | | | | | | | | | | | | | | | |  | | | | | | | | | | | | | | |  | | | | | | | | | | | | | | |  | | | | | | | | | | | | | |  | | | | | | | | | | | | | |  | | | | | | | | |  | | | | | | | | | | | | | | |  | | | | | | | | | | | | | | | | | | | | |  | | | | | | | | | | |  | | | | | | | | | | | | | | | | | | |  | | | | | | | | | | | |  | | | | | | | | |  | | | | | | | | | | | | | | | | | | | |  | | | | | | | | | | | | | | | |  | | | | | | | | | | | | | |  | | | | | | | | | | | | |  | | | | | | | | | | | |  | | | | | | | | | | | | | | | | | | | |  | | | | | | | | | | | | | | | | | |  | | | | | | | | | | | | | | | | |  | | | | | | | | | | | | | | | |  | | | | | | | | | | | | | | | | | | | | | | | | | | | | | | | | | | |  | |  |  |  |  |
| **Vector control** | | | | | | | | | | | | | | | | | | | | | | | | | | | | | | | | | | | | | | | | | | | | | | | | | | | | | | | | | | | | | | | | | | | | | | | | | | | | | | | | | | | | | | | | | | | | | | | | | | | | | | | | | | | | | | | | | | | | | | | | | | | | | | | | | | | |  | | | | | | | | | | | | | | |  | | | | | | | | | | | | | | |  | | | | | | | | | | | | | |  | | | | | | | | | | | | | |  | | | | | | | | |  | | | | | | | | | | | | | | |  | | | | | | | | | | | | | | | | | | | | |  | | | | | | | | | | |  | | | | | | | | | | | | | | | | | | |  | | | | | | | | | | | |  | | | | | | | | |  | | | | | | | | | | | | | | | | | | | |  | | | | | | | | | | | | | | | |  | | | | | | | | | | | | | |  | | | | | | | | | | | | |  | | | | | | | | | | | |  | | | | | | | | | | | | | | | | | | | |  | | | | | | | | | | | | | | | | | |  | | | | | | | | | | | | | | | | |  | | | | | | | | | | | | | | | |  | | | | | | | | | | | | | | | | | | | | | | | | | | | | | | | | | | |  | |  |  |  |  |
| **8- Can reducing mosquitoes help in reducing malaria?** | | | | | | | | | | | | | | | | | | | | | | | | | | | | | | | | | | | | | | | | | | | | | | | | | | | | | | | | | | | | | | | | | | | | | | | | | | | | | | | | | | | | | | | | | | | | | | | | | | | | | | | | | | | | | | | | | | | | | | | | | | | | | | | | | | | | | | | | | | | | | | | | | | | | | | | | | | | | | | | | | | | | | | | | | | | | | | | | | | | | | | | | | | | | | | | | | | | | | | | | | | | | | | | | | | | | | | | | | | | | | | | | | | | | | | | | | **9- What do you think which measures can reduce the number of mosquitoes ?** | | | | | | | | | | | | | | | | | | | | | | | | | | | | | | | | | | | | | | | | | | | | | | | | | | | | | | | | | | | | | | | | | | | | | | | | | | | | | | | | | | | | | | | | | | | | | | | | | | | | | | | | | | | | | | | | | | | | | | | | | | | | | | | | | | | | | | | | | | | | | | | | | | |  | | | | | | | | | | | | | | | |  | | | | | | | | | | | | | | | | | |  | | | | | | | | | | | | | | | |  | | | | | | | | | | | | | | | | | | | | | | | | | | | | | | | | | | | | |  | |  |  |  |  |
|  | | | | | | | 1- Yes | | | | | | | | | | | | | | | | | |  | | | | | | | | | | | | | | | |  | | | | | | |  | | | | | | | | | | | |  | | | | | | | | | | | |  | | | | | | | | | | | | | |  | | | | | | | | | | |  | | | | | | | | | | | | | | |  | | | | | | | | | | | | | | | | | | | |  | | | | | | | | | | | | | | |  | | | | | | | | | | | | | | |  | | | | | | | | | | | | | |  | | | | | | | | | | | | | |  | | | | | | | | |  | | | | | | | | | | | | | | |  | | | | | | | | | | | | | | | | | | | | |  | | | | | | | | | | |  | | | | | | | | | | | | | | | | | | | | | | | | | | | | | | | | | | | | | | | | | | | | | | | | | | | | | | | | | | | | | | | | | | | | | | | | | | | | | | | | | | | | | | | | | | | | | | | | | | | | | | | | | | | | | | | | | | | | | | | | | | | | | | | | | | | | | | | | | | | | | | | | | | | | | | | | | | | | | | | | | | | | | | | | | |  | | | | | | | | | | | | | | | |  | | | | | | | | | | | | | | | | | | | | | | | | | | | | | | | | | | |  | |  |  |  |  |
|  | | | | | | | 2- No | | | | | | | | | | | | | | | | | |  | | | | | | | | | | | | | | | |  | | | | | | |  | | | | | | | | | | | |  | | | | | | | | | | | |  | | | | | | | | | | | | | |  | | | | | | | | | | |  | | | | | | | | | | | | | | |  | | | | | | | | | | | | | | | | | | | |  | | | | | | | | | | | | | | |  | | | | | | | | | | | | | | |  | | | | | | | | | | | | | |  | | | | | | | | | | | | | |  | | | | | | | | |  | | | | | | | | | | | | | | |  | | | | | | | | | | | | | | | | | | | | |  | | | | | | | | | | |  | | | | | | | | | | | | | | | | | | |  | | | | | | | | | | | |  | | | | | | | | |  | | | | | | | | | | | | | | | | | | | |  | | | | | | | | | | | | | | | |  | | | | | | | | | | | | | |  | | | | | | | | | | | | |  | | | | | | | | | | | |  | | | | | | | | | | | | | | | | | | | |  | | | | | | | | | | | | | | | | | |  | | | | | | | | | | | | | | | | |  | | | | | | | | | | | | | | | |  | | | | | | | | | | | | | | | | | | | | | | | | | | | | | | | | | | |  | |  |  |  |  |
|  | | | | | | | 3- Don´t know | | | | | | | | | | | | | | | | | | | | | | | | | | | | | | | | | | | | | | | | |  | | | | | | | | | | | |  | | | | | | | | | | | |  | | | | | | | | | | | | | |  | | | | | | | | | | |  | | | | | | | | | | | | | | |  | | | | | | | | | | | | | | | | | | | |  | | | | | | | | | | | | | | |  | | | | | | | | | | | | | | |  | | | | | | | | | | | | | |  | | | | | | | | | | | | | |  | | | | | | | | |  | | | | | | | | | | | | | | |  | | | | | | | | | | | | | | | | | | | | |  | | | | | | | | | | | 1- Drain / dry up stagnant waters | | | | | | | | | | | | | | | | | | | | | | | | | | | | | | | | | | | | | | | | | | | | | | | | | | | | | | | | | | | | | | | | | | | | | | | | | | | | | | | | | | | | | | | | | | | | | | | | | | | | | | | | | | | | | | | | | | | | | | | | | | | | | | | | | | | | | | | | | | | | | | | | | | | | | | | | |  | | | | | | | | | | | | | | | | |  | | | | | | | | | | | | | | | |  | | | | | | | | | | | | | | | | | | | | | | | | | | | | | | | | | | |  | |  |  |  |  |
| **10- What do you think which activities can reduce the presence of mosquito larvae ?** | | | | | | | | | | | | | | | | | | | | | | | | | | | | | | | | | | | | | | | | | | | | | | | | | | | | | | | | | | | | | | | | | | | | | | | | | | | | | | | | | | | | | | | | | | | | | | | | | | | | | | | | | | | | | | | | | | | | | | | | | | | | | | | | | | | | | | | | | | | | | | | | | | | | | | | | | | | | | | | | | | | | | | | | | | | | | | | | | | | | | | | | | | | | | | | | | | | | | | | | | | | | | | | | | | | | | | | | | | | | | | | | | | | | | | | | |  | | | | | | | | | | | 2- Cut grass and bushes around houses | | | | | | | | | | | | | | | | | | | | | | | | | | | | | | | | | | | | | | | | | | | | | | | | | | | | | | | | | | | | | | | | | | | | | | | | | | | | | | | | | | | | | | | | | | | | | | | | | | | | | | | | | | | | | | | | | | | | | | | | | | | | | | | | | | | | | | | | | | | | | | | | | | | | | | | | | | | | | | | | | | | | | | | | | | | | | | | | | | | | | | | | | | | | | | | | | | | | | | | | | | | | | | | | | | | | | | | | | | | | | | |  | |  |  |  |  |
|  | | | | | | | 1- Drain / dry up stagnant waters | | | | | | | | | | | | | | | | | | | | | | | | | | | | | | | | | | | | | | | | | | | | | | | | | | | | | | | | | | | | | | | | | | | | | | | | | | | | | | | | | | | | | | | | | | | | | | | | | | | | | | | | | | | | | | | | | | | | | | | | | | | | | | | | | | | | | | | | | | | | | | | | | | | | | | | | | | | | | | | | | | | | | | | | | | | | | | | | | | | | | | |  | | | | | | | | |  | | | | | | | | | | | | | | |  | | | | | | | | | | | | | | | | | | | | |  | | | | | | | | | | | 3- Use of insecticides | | | | | | | | | | | | | | | | | | | | | | | | | | | | | | | | | | | | | | | | | | | | | | | | | | | | | | | | | | | | | | | | | | | | | | | | | | | | | | | | | | | | | | | | | | | | | | | | | | | | | | |  | | | | | | | | | | | |  | | | | | | | | | | | | | | | | | | | |  | | | | | | | | | | | | | | | | | |  | | | | | | | | | | | | | | | | |  | | | | | | | | | | | | | | | |  | | | | | | | | | | | | | | | | | | | | | | | | | | | | | | | | | | |  | |  |  |  |  |
|  | | | | | | | 2 - Cut grass and bushes around houses | | | | | | | | | | | | | | | | | | | | | | | | | | | | | | | | | | | | | | | | | | | | | | | | | | | | | | | | | | | | | | | | | | | | | | | | | | | | | | | | | | | | | | | | | | | | | | | | | | | | | | | | | | | | | | | | | | | | | | | | | | | | | | | | | | | | | | | | | | | | | | | | | | | | | | | | | | |  | | | | | | | | | | | | | |  | | | | | | | | | | | | | |  | | | | | | | | |  | | | | | | | | | | | | | | |  | | | | | | | | | | | | | | | | | | | | |  | | | | | | | | | | | 4- Use of larvicides | | | | | | | | | | | | | | | | | | | | | | | | | | | | | | | | | | | | | | | | | | | | | | | | | | | | | | | | | | | | | | | | | | | | | | | | | | | | | | | | | | | | | | | | | | | | | | | | | | | | | | |  | | | | | | | | | | | |  | | | | | | | | | | | | | | | | | | | |  | | | | | | | | | | | | | | | | | |  | | | | | | | | | | | | | | | | |  | | | | | | | | | | | | | | | |  | | | | | | | | | | | | | | | | | | | | | | | | | | | | | | | | | | |  | |  |  |  |  |
|  | | | | | | | 3- Use of larvicides | | | | | | | | | | | | | | | | | | | | | | | | | | | | | | | | | | | | | | | | | | | | | | | | | | | | | | | | | | | | | | | | | | | | | | | | | | | | | | |  | | | | | | | | | | |  | | | | | | | | | | | | | | |  | | | | | | | | | | | | | | | | | | | |  | | | | | | | | | | | | | | |  | | | | | | | | | | | | | | |  | | | | | | | | | | | | | |  | | | | | | | | | | | | | |  | | | | | | | | |  | | | | | | | | | | | | | | |  | | | | | | | | | | | | | | | | | | | | |  | | | | | | | | | | | 5- Remove waste from patios | | | | | | | | | | | | | | | | | | | | | | | | | | | | | | | | | | | | | | | | | | | | | | | | | | | | | | | | | | | | | | | | | | | | | | | | | | | | | | | | | | | | | | | | | | | | | | | | | | | | | | | | | | | | | | | | | | | | | | | | | | | | | | | | | | | | | | | | | | | | | | | | | | | | | | | | | | | | | | | | | | | | | | | | | | | | | | | | | | | | | | | | | |  | | | | | | | | | | | | | | | | | | | | | | | | | | | | | | | | | | |  | |  |  |  |  |
|  | | | | | | | 4- Remove waste from patios | | | | | | | | | | | | | | | | | | | | | | | | | | | | | | | | | | | | | | | | | | | | | | | | | | | | | | | | | | | | | | | | | | | | | | | | | | | | | | | | | | | | | | | | | | | | | | | | | | | | | | | | | | | | | | | | | | | | | | | | | | | | | | | | | | | | | | | | | | | | | | | | | | | | | | | | | | |  | | | | | | | | | | | | | |  | | | | | | | | | | | | | |  | | | | | | | | |  | | | | | | | | | | | | | | |  | | | | | | | | | | | | | | | | | | | | |  | | | | | | | | | | | 6- Use of mosquito nets | | | | | | | | | | | | | | | | | | | | | | | | | | | | | | | | | | | | | | | | | | | | | | | | | | | | | | | | | | | | | | | | | | | | | | | | | | | | | | | | | | | | | | | | | | | | | | | | | | | | | | | | | | | | | | | | | | |  | | | | | | | | | | | | | | | | | | | |  | | | | | | | | | | | | | | | | | |  | | | | | | | | | | | | | | | | |  | | | | | | | | | | | | | | | |  | | | | | | | | | | | | | | | | | | | | | | | | | | | | | | | | | | |  | |  |  |  |  |
|  | | | | | | | 5- Don´t know | | | | | | | | | | | | | | | | | | | | | | | | | | | | | | | | | | | | | | | | |  | | | | | | | | | | | |  | | | | | | | | | | | |  | | | | | | | | | | | | | |  | | | | | | | | | | |  | | | | | | | | | | | | | | |  | | | | | | | | | | | | | | | | | | | |  | | | | | | | | | | | | | | |  | | | | | | | | | | | | | | |  | | | | | | | | | | | | | |  | | | | | | | | | | | | | |  | | | | | | | | |  | | | | | | | | | | | | | | |  | | | | | | | | | | | | | | | | | | | | |  | | | | | | | | | | |  | | | | | | | | | | | | | | | | | | |  | | | | | | | | | | | |  | | | | | | | | |  | | | | | | | | | | | | | | | | | | | |  | | | | | | | | | | | | | | | |  | | | | | | | | | | | | | |  | | | | | | | | | | | | |  | | | | | | | | | | | |  | | | | | | | | | | | | | | | | | | | |  | | | | | | | | | | | | | | | | | |  | | | | | | | | | | | | | | | | |  | | | | | | | | | | | | | | | |  | | | | | | | | | | | | | | | | | | | | | | | | | | | | | | | | | | |  | |  |  |  |  |
|  | | | | | | |  | | | | | | | | | |  | | | | | | | |  | | | | | | | | | | | | | | | |  | | | | | | |  | | | | | | | | | | | |  | | | | | | | | | | | |  | | | | | | | | | | | | | |  | | | | | | | | | | |  | | | | | | | | | | | | | | |  | | | | | | | | | | | | | | | | | | | |  | | | | | | | | | | | | | | |  | | | | | | | | | | | | | | |  | | | | | | | | | | | | | |  | | | | | | | | | | | | | |  | | | | | | | | |  | | | | | | | | | | | | | | |  | | | | | | | | | | | | | | | | | | | | |  | | | | | | | | | | |  | | | | | | | | | | | | | | | | | | |  | | | | | | | | | | | |  | | | | | | | | |  | | | | | | | | | | | | | | | | | | | |  | | | | | | | | | | | | | | | |  | | | | | | | | | | | | | |  | | | | | | | | | | | | |  | | | | | | | | | | | |  | | | | | | | | | | | | | | | | | | | |  | | | | | | | | | | | | | | | | | |  | | | | | | | | | | | | | | | | |  | | | | | | | | | | | | | | | |  | | | | | | | | | | | | | | | | | | | | | | | | | | | | | | | | | | |  | |  |  |  |  |
| **Knowledge, perception and acceptance of the project** | | | | | | | | | | | | | | | | | | | | | | | | | | | | | | | | | | | | | | | | | | | | | | | | | | | | | | | | | | | | | | | | | | | | | | | | | | | | | | | | | | | | | | | | | | | | | | | | | | | | | | | | | | | | | | | | | | | | | | | | | | | | | | | | | | | | | | | | | | | | | | | | | | | | | | | | | | | | | | | | | | | | | | | | | | | | | | | | | | | | | | | | | | | | | |  | | | | | | | | |  | | | | | | | | | | | | | | |  | | | | | | | | | | | | | | | | | | | | |  | | | | | | | | | | |  | | | | | | | | | | | | | | | | | | |  | | | | | | | | | | | |  | | | | | | | | |  | | | | | | | | | | | | | | | | | | | |  | | | | | | | | | | | | | | | |  | | | | | | | | | | | | | |  | | | | | | | | | | | | |  | | | | | | | | | | | |  | | | | | | | | | | | | | | | | | | | |  | | | | | | | | | | | | | | | | | |  | | | | | | | | | | | | | | | | |  | | | | | | | | | | | | | | | |  | | | | | | | | | | | | | | | | | | | | | | | | | | | | | | | | | | |  | |  |  |  |  |
|  | | | | | | |  | | | | | | | | | |  | | | | | | | |  | | | | | | | | | | | | | | | |  | | | | | | |  | | | | | | | | | | | |  | | | | | | | | | | | |  | | | | | | | | | | | | | |  | | | | | | | | | | |  | | | | | | | | | | | | | | |  | | | | | | | | | | | | | | | | | | | |  | | | | | | | | | | | | | | |  | | | | | | | | | | | | | | |  | | | | | | | | | | | | | |  | | | | | | | | | | | | | |  | | | | | | | | |  | | | | | | | | | | | | | | |  | | | | | | | | | | | | | | | | | | | | |  | | | | | | | | | | |  | | | | | | | | | | | | | | | | | | |  | | | | | | | | | | | |  | | | | | | | | |  | | | | | | | | | | | | | | | | | | | |  | | | | | | | | | | | | | | | |  | | | | | | | | | | | | | |  | | | | | | | | | | | | |  | | | | | | | | | | | |  | | | | | | | | | | | | | | | | | | | |  | | | | | | | | | | | | | | | | | |  | | | | | | | | | | | | | | | | |  | | | | | | | | | | | | | | | |  | | | | | | | | | | | | | | | | | | | | | | | | | | | | | | | | | | |  | |  |  |  |  |
| **11- Did you know about larviciding actions taking place in your village to eliminate mosquitoes ?** | | | | | | | | | | | | | | | | | | | | | | | | | | | | | | | | | | | | | | | | | | | | | | | | | | | | | | | | | | | | | | | | | | | | | | | | | | | | | | | | | | | | | | | | | | | | | | | | | | | | | | | | | | | | | | | | | | | | | | | | | | | | | | | | | | | | | | | | | | | | | | | | | | | | | | | | | | | | | | | | | |  | | | | | | | | | | | | | |  | | | | | | | | | | | | | |  | | | | | | | | | **12- Did you already observe community based sprayers in you village / sector ?** | | | | | | | | | | | | | | | | | | | | | | | | | | | | | | | | | | | | | | | | | | | | | | | | | | | | | | | | | | | | | | | | | | | | | | | | | | | | | | | | | | | | | | | | | | | | | | | | | | | | | | | | | | | | | | | | | | | | | | | | | | | | | | | | | | | | | | | | | | | | | | | | | | | | | | | | | | | | | | | | | | | | | | | | | | | | | | | | | | | | | | | | | | | | | | | | | | | | | | | | | | | | | | | | | | | | | | | | | | | | | | | | | | | | | | | | |  | | | | | | | | | | | | | | | | | | | | | | | | | | | | | | | | | | |  | |  |  |  |  |
|  | | | | | | |  | | | | | | | | | | | | | | | | | | | | | | | | | | | | | | | | | | | | | | | | | | | | | | | | | | | | | | | | | | | | | | | | | | | | | | | | | | | | | | | | | | | | | | | | | | | | | | | | | | | | | | | | | | | | | | | | | | | | | | | | | | | | | | | | | | | | | | | | | | | | | | | | | | | | | | | | | | | | | | | | | | | | | | | | | | | | | | | | | | | | | | |  | | | | | | | | |  | | | | | | | | | | | | | | |  | | | | | | | | | | | | | | | | | | | | |  | | | | | | | | | | |  | | | | | | | | | | | | | | | | | | | | | | | | | | | | | | | | | | | | | | | | | | | | | | | | | | | | | | | | | | | | | | | | | | | | | | | | | | | | | | | | | | | | | | | | | | | | | | | | | | | | | | | | | | | | | | | | | | | | | | | | | | | | | | | | | | | | | | | | | | | | | | | | | | | | | | | | | | | | | | | | | | | | | | | | | | | | | | | | | | | | | | | | | |  | | | | | | | | | | | | | | | | | | | | | | | | | | | | | | | | | | |  | |  |  |  |  |
|  | | | | | | | 1- Yes | | | | | | | | | | | | | | | | | |  | | | | | | | | | | | | | | | |  | | | | | | |  | | | | | | | | | | | |  | | | | | | | | | | | |  | | | | | | | | | | | | | |  | | | | | | | | | | |  | | | | | | | | | | | | | | |  | | | | | | | | | | | | | | | | | | | |  | | | | | | | | | | | | | | |  | | | | | | | | | | | | | | |  | | | | | | | | | | | | | |  | | | | | | | | | | | | | |  | | | | | | | | |  | | | | | | | | | | | | | | |  | | | | | | | | | | | | | | | | | | | | |  | | | | | | | | | | | 1- Yes | | | | | | | | | | | | | | | | | | | | | | | | | | | | | | |  | | | | | | | | |  | | | | | | | | | | | | | | | | | | | |  | | | | | | | | | | | | | | | |  | | | | | | | | | | | | | |  | | | | | | | | | | | | |  | | | | | | | | | | | |  | | | | | | | | | | | | | | | | | | | |  | | | | | | | | | | | | | | | | | |  | | | | | | | | | | | | | | | | |  | | | | | | | | | | | | | | | |  | | | | | | | | | | | | | | | | | | | | | | | | | | | | | | | | | | |  | |  |  |  |  |
|  | | | | | | | 2- No | | | | | | | | | | | | | | | | | |  | | | | | | | | | | | | | | | |  | | | | | | |  | | | | | | | | | | | |  | | | | | | | | | | | |  | | | | | | | | | | | | | |  | | | | | | | | | | |  | | | | | | | | | | | | | | |  | | | | | | | | | | | | | | | | | | | |  | | | | | | | | | | | | | | |  | | | | | | | | | | | | | | |  | | | | | | | | | | | | | |  | | | | | | | | | | | | | |  | | | | | | | | |  | | | | | | | | | | | | | | |  | | | | | | | | | | | | | | | | | | | | |  | | | | | | | | | | | 2- No | | | | | | | | | | | | | | | | | | | | | | | | | | | | | | |  | | | | | | | | |  | | | | | | | | | | | | | | | | | | | |  | | | | | | | | | | | | | | | |  | | | | | | | | | | | | | |  | | | | | | | | | | | | |  | | | | | | | | | | | |  | | | | | | | | | | | | | | | | | | | |  | | | | | | | | | | | | | | | | | |  | | | | | | | | | | | | | | | | |  | | | | | | | | | | | | | | | |  | | | | | | | | | | | | | | | | | | | | | | | | | | | | | | | | | | |  | |  |  |  |  |
|  | | | | | | | 3- Don´t know | | | | | | | | | | | | | | | | | | | | | | | | | | | | | | | | | | | | | | | | |  | | | | | | | | | | | |  | | | | | | | | | | | |  | | | | | | | | | | | | | |  | | | | | | | | | | |  | | | | | | | | | | | | | | |  | | | | | | | | | | | | | | | | | | | |  | | | | | | | | | | | | | | |  | | | | | | | | | | | | | | |  | | | | | | | | | | | | | |  | | | | | | | | | | | | | |  | | | | | | | | |  | | | | | | | | | | | | | | |  | | | | | | | | | | | | | | | | | | | | |  | | | | | | | | | | | 3- Don´t know | | | | | | | | | | | | | | | | | | | | | | | | | | | | | | | | | | | | | | | | | | | | | | | | | | | | | | | | | | | |  | | | | | | | | | | | | | | | |  | | | | | | | | | | | | | |  | | | | | | | | | | | | |  | | | | | | | | | | | |  | | | | | | | | | | | | | | | | | | | |  | | | | | | | | | | | | | | | | | |  | | | | | | | | | | | | | | | | |  | | | | | | | | | | | | | | | |  | | | | | | | | | | | | | | | | | | | | | | | | | | | | | | | | | | |  | |  |  |  |  |
| **13- How did you hear the first time about this activity ?** | | | | | | | | | | | | | | | | | | | | | | | | | | | | | | | | | | | | | | | | | | | | | | | | | | | | | | | | | | | | | | | | | | | | | | | | | | | | | | | | | | | | | | | | | | | | | | | | | | | | | | | | | | | | | | | | | | | | | | | | | | | | | | | | | | | | | | | | | | | | | | | | | | | | | | | | | | | | | | | | | | | | | | | | | | | | | | | | | | | | | | | | | | | | | | | | | | | | | | | | | | | | | | | | | | | | | | | | | | | | | | | | | | | | | | | | | **14- What do you think about this intervention ?** | | | | | | | | | | | | | | | | | | | | | | | | | | | | | | | | | | | | | | | | | | | | | | | | | | | | | | | | | | | | | | | | | | | | | | | | | | | | | | | | | | | | | | | | | | | | | | | | | | | | | | | | | | | | | | | | | | | | | | | | | | | | | | | | | | | | | | | | | | | | | | | | | | | | | | | | | | | | | | | | | | |  | | | | | | | | | | | | | | | | | |  | | | | | | | | | | | | | | | |  | | | | | | | | | | | | | | | | | | | | | | | | | | | | | | | | | | | | |  | |  |  |  |  |
|  | | | | | | | 1- Saw it myself | | | | | | | | | | | | | | | | | | | | | | | | | | | | | | | | | | | | | | | | | | | | | | | | | | | | | | | | | | | | | | | | | | | | | | | | | | | | | | |  | | | | | | | | | | |  | | | | | | | | | | | | | | |  | | | | | | | | | | | | | | | | | | | |  | | | | | | | | | | | | | | |  | | | | | | | | | | | | | | |  | | | | | | | | | | | | | |  | | | | | | | | | | | | | |  | | | | | | | | |  | | | | | | | | | | | | | | |  | | | | | | | | | | | | | | | | | | | | |  | | | | | | | | | | | 1- Very good | | | | | | | | | | | | | | | | | | | | | | | | | | | | | | | | | | | | | | | | | | | | | | | | | | | | | | | | | | | |  | | | | | | | | | | | | | | | |  | | | | | | | | | | | | | |  | | | | | | | | | | | | |  | | | | | | | | | | | |  | | | | | | | | | | | | | | | | | | | |  | | | | | | | | | | | | | | | | | |  | | | | | | | | | | | | | | | | |  | | | | | | | | | | | | | | | |  | | | | | | | | | | | | | | | | | | | | | | | | | | | | | | | | | | |  | |  |  |  |  |
|  | | | | | | | 2- By someone from my village / sector | | | | | | | | | | | | | | | | | | | | | | | | | | | | | | | | | | | | | | | | | | | | | | | | | | | | | | | | | | | | | | | | | | | | | | | | | | | | | | | | | | | | | | | | | |  | | | | | | | | | | | | | | |  | | | | | | | | | | | | | | | | | | | |  | | | | | | | | | | | | | | |  | | | | | | | | | | | | | | |  | | | | | | | | | | | | | |  | | | | | | | | | | | | | |  | | | | | | | | |  | | | | | | | | | | | | | | |  | | | | | | | | | | | | | | | | | | | | |  | | | | | | | | | | | 2- Good | | | | | | | | | | | | | | | | | | | | | | | | | | | | | | | | | | | | | | | |  | | | | | | | | | | | | | | | | | | | |  | | | | | | | | | | | | | | | |  | | | | | | | | | | | | | |  | | | | | | | | | | | | |  | | | | | | | | | | | |  | | | | | | | | | | | | | | | | | | | |  | | | | | | | | | | | | | | | | | |  | | | | | | | | | | | | | | | | |  | | | | | | | | | | | | | | | |  | | | | | | | | | | | | | | | | | | | | | | | | | | | | | | | | | | |  | |  |  |  |  |
|  | | | | | | | 3- By a household memeber | | | | | | | | | | | | | | | | | | | | | | | | | | | | | | | | | | | | | | | | | | | | | | | | | | | | | | | | | | | | | | | | | | | | | | | | | | | | | | | | | | | | | | | | | |  | | | | | | | | | | | | | | |  | | | | | | | | | | | | | | | | | | | |  | | | | | | | | | | | | | | |  | | | | | | | | | | | | | | |  | | | | | | | | | | | | | |  | | | | | | | | | | | | | |  | | | | | | | | |  | | | | | | | | | | | | | | |  | | | | | | | | | | | | | | | | | | | | |  | | | | | | | | | | | 3-OK | | | | | | | | | | | | | | | | | | | | | | | | | | | | | | | | | | | | | | | |  | | | | | | | | | | | | | | | | | | | |  | | | | | | | | | | | | | | | |  | | | | | | | | | | | | | |  | | | | | | | | | | | | |  | | | | | | | | | | | |  | | | | | | | | | | | | | | | | | | | |  | | | | | | | | | | | | | | | | | |  | | | | | | | | | | | | | | | | |  | | | | | | | | | | | | | | | |  | | | | | | | | | | | | | | | | | | | | | | | | | | | | | | | | | | |  | |  |  |  |  |
|  | | | | | | | 4- By a health worker | | | | | | | | | | | | | | | | | | | | | | | | | | | | | | | | | | | | | | | | | | | | | | | | | | | | | | | | | | | | | | | | |  | | | | | | | | | | | | | |  | | | | | | | | | | |  | | | | | | | | | | | | | | |  | | | | | | | | | | | | | | | | | | | |  | | | | | | | | | | | | | | |  | | | | | | | | | | | | | | |  | | | | | | | | | | | | | |  | | | | | | | | | | | | | |  | | | | | | | | |  | | | | | | | | | | | | | | |  | | | | | | | | | | | | | | | | | | | | |  | | | | | | | | | | | 4- Bad | | | | | | | | | | | | | | | | | | | | | | | | | | | | | | | | | | | | | | | | | | | | | | | | | | | | | | | | | | | |  | | | | | | | | | | | | | | | |  | | | | | | | | | | | | | |  | | | | | | | | | | | | |  | | | | | | | | | | | |  | | | | | | | | | | | | | | | | | | | |  | | | | | | | | | | | | | | | | | |  | | | | | | | | | | | | | | | | |  | | | | | | | | | | | | | | | |  | | | | | | | | | | | | | | | | | | | | | | | | | | | | | | | | | | |  | |  |  |  |  |
|  | | | | | | | 5- From the radio | | | | | | | | | | | | | | | | | | | | | | | | | | | | | | | | | | | | | | | | |  | | | | | | | | | | | |  | | | | | | | | | | | |  | | | | | | | | | | | | | |  | | | | | | | | | | |  | | | | | | | | | | | | | | |  | | | | | | | | | | | | | | | | | | | |  | | | | | | | | | | | | | | |  | | | | | | | | | | | | | | |  | | | | | | | | | | | | | |  | | | | | | | | | | | | | |  | | | | | | | | |  | | | | | | | | | | | | | | |  | | | | | | | | | | | | | | | | | | | | |  | | | | | | | | | | |  | | | | | | | | | | | | | | | | | | |  | | | | | | | | | | | |  | | | | | | | | |  | | | | | | | | | | | | | | | | | | | |  | | | | | | | | | | | | | | | |  | | | | | | | | | | | | | |  | | | | | | | | | | | | |  | | | | | | | | | | | |  | | | | | | | | | | | | | | | | | | | |  | | | | | | | | | | | | | | | | | |  | | | | | | | | | | | | | | | | |  | | | | | | | | | | | | | | | |  | | | | | | | | | | | | | | | | | | | | | | | | | | | | | | | | | | |  | |  |  |  |  |
|  | | | | | | | 6- Other (specify ………………………………………….) | | | | | | | | | | | | | | | | | | | | | | | | | | | | | | | | | | | | | | | | | | | | | | | | | | | | | | | | | | | | | | | | | | | | | | | | | | | | | | | | | | | | | | | | | | | | | | | | | | | | | | | | | | | | | | | | | | | | | | | | | | | | | | | | | | | | | | | | | | | |  | | | | | | | | | | | | | | |  | | | | | | | | | | | | | |  | | | | | | | | | | | | | |  | | | | | | | | |  | | | | | | | | | | | | | | |  | | | | | | | | | | | | | | | | | | | | |  | | | | | | | | | | |  | | | | | | | | | | | | | | | | | | |  | | | | | | | | | | | |  | | | | | | | | |  | | | | | | | | | | | | | | | | | | | |  | | | | | | | | | | | | | | | |  | | | | | | | | | | | | | |  | | | | | | | | | | | | |  | | | | | | | | | | | |  | | | | | | | | | | | | | | | | | | | |  | | | | | | | | | | | | | | | | | |  | | | | | | | | | | | | | | | | |  | | | | | | | | | | | | | | | |  | | | | | | | | | | | | | | | | | | | | | | | | | | | | | | | | | | |  | |  |  |  |  |
|  | | | | | | |  | | | | | | | | | |  | | | | | | | |  | | | | | | | | | | | | | | | |  | | | | | | |  | | | | | | | | | | | |  | | | | | | | | | | | |  | | | | | | | | | | | | | |  | | | | | | | | | | |  | | | | | | | | | | | | | | |  | | | | | | | | | | | | | | | | | | | |  | | | | | | | | | | | | | | |  | | | | | | | | | | | | | | |  | | | | | | | | | | | | | |  | | | | | | | | | | | | | |  | | | | | | | | |  | | | | | | | | | | | | | | |  | | | | | | | | | | | | | | | | | | | | |  | | | | | | | | | | |  | | | | | | | | | | | | | | | | | | |  | | | | | | | | | | | |  | | | | | | | | |  | | | | | | | | | | | | | | | | | | | |  | | | | | | | | | | | | | | | |  | | | | | | | | | | | | | |  | | | | | | | | | | | | |  | | | | | | | | | | | |  | | | | | | | | | | | | | | | | | | | |  | | | | | | | | | | | | | | | | | |  | | | | | | | | | | | | | | | | |  | | | | | | | | | | | | | | | |  | | | | | | | | | | | | | | | | | | | | | | | | | | | | | | | | | | |  | |  |  |  |  |
| **15- What do you think is the community´s opinion on this intervention?** | | | | | | | | | | | | | | | | | | | | | | | | | | | | | | | | | | | | | | | | | | | | | | | | | | | | | | | | | | | | | | | | | | | | | | | | | | | | | | | | | | | | | | | | | | | | | | | | | | | | | | | | | | | | | | | | | | | | | | | | | | | | | | | | | | | | | | | | | | | | | | | | | | | | | | | | | | | | | | | | | | | | | | | | | | | | | | | | | | | | | | | | | | | | | | | | | | | | | | | | | | | | | | | | | | | | | | | | | | | | | | | | | | | | | | | | | **16- Did you observe any changes after the intervention started ?** | | | | | | | | | | | | | | | | | | | | | | | | | | | | | | | | | | | | | | | | | | | | | | | | | | | | | | | | | | | | | | | | | | | | | | | | | | | | | | | | | | | | | | | | | | | | | | | | | | | | | | | | | | | | | | | | | | | | | | | | | | | | | | | | | | | | | | | | | | | | | | | | | | | | | | | | | | | | | | | | | | | | | | | | | | | | | | | | | | | | |  | | | | | | | | | | | | | | | |  | | | | | | | | | | | | | | | | | | | | | | | | | | | | | | | | | | | | |  | |  |  |  |  |
|  | | | | | | | 1- Very good | | | | | | | | | | | | | | | | | | | | | | | | | | | | | | | | | | | | | | | | |  | | | | | | | | | | | |  | | | | | | | | | | | |  | | | | | | | | | | | | | |  | | | | | | | | | | |  | | | | | | | | | | | | | | |  | | | | | | | | | | | | | | | | | | | |  | | | | | | | | | | | | | | |  | | | | | | | | | | | | | | |  | | | | | | | | | | | | | |  | | | | | | | | | | | | | |  | | | | | | | | |  | | | | | | | | | | | | | | |  | | | | | | | | | | | | | | | | | | | | |  | | | | | | | | | | |  | | | | | | | | | | | | | | | | | | | | | | | | | | | | | | | | | | | | | | | | | | | | | | | | | | | | | | | | | | | | | | | | | | | | | | | | | | | | | | | | | | | | | | | | | | | | | | | | | | | | | | | | | | | | | | | | | | |  | | | | | | | | | | | | | | | | | | | |  | | | | | | | | | | | | | | | | | |  | | | | | | | | | | | | | | | | |  | | | | | | | | | | | | | | | |  | | | | | | | | | | | | | | | | | | | | | | | | | | | | | | | | | | |  | |  |  |  |  |
|  | | | | | | | 2- Good | | | | | | | | | | | | | | | | | | | | | | | | | | | | | | | | | |  | | | | | | |  | | | | | | | | | | | |  | | | | | | | | | | | |  | | | | | | | | | | | | | |  | | | | | | | | | | |  | | | | | | | | | | | | | | |  | | | | | | | | | | | | | | | | | | | |  | | | | | | | | | | | | | | |  | | | | | | | | | | | | | | |  | | | | | | | | | | | | | |  | | | | | | | | | | | | | |  | | | | | | | | |  | | | | | | | | | | | | | | |  | | | | | | | | | | | | | | | | | | | | |  | | | | | | | | | | | 1- Yes | | | | | | | | | | | | | | | | | | | | | | | | | | | | | | |  | | | | | | | | |  | | | | | | | | | | | | | | | | | | | | **16****-b** | | | | | | | | | | | | | | | | **Which?** | | | | | | | | | | | | | | | | | | | | | | | | | | | | | | | | | | | | | | |  | | | | | | | | | | | | | | | | | | | |  | | | | | | | | | | | | | | | | | |  | | | | | | | | | | | | | | | | |  | | | | | | | | | | | | | | | |  | | | | | | | | | | | | | | | | | | | | | | | | | | | | | | | | | | |  | |  |  |  |  |
|  | | | | | | | 3-OK | | | | | | | | | | | | | | | | | | | | | | | | | | | | | | | | | |  | | | | | | |  | | | | | | | | | | | |  | | | | | | | | | | | |  | | | | | | | | | | | | | |  | | | | | | | | | | |  | | | | | | | | | | | | | | |  | | | | | | | | | | | | | | | | | | | |  | | | | | | | | | | | | | | |  | | | | | | | | | | | | | | |  | | | | | | | | | | | | | |  | | | | | | | | | | | | | |  | | | | | | | | |  | | | | | | | | | | | | | | |  | | | | | | | | | | | | | | | | | | | | |  | | | | | | | | | | | 2- No | | | | | | | | | | | | | | | | | | | | | | | | | | | | | | |  | | | | | | | | |  | | | | | | | | | | | | | | | | | | | |  | | | | | | | | | | | | | | | |  | | | | | | | | | | | | | |  | | | | | | | | | | | | |  | | | | | | | | | | | |  | | | | | | | | | | | | | | | | | | | |  | | | | | | | | | | | | | | | | | |  | | | | | | | | | | | | | | | | |  | | | | | | | | | | | | | | | |  | | | | | | | | | | | | | | | | | | | | | | | | | | | | | | | | | | |  | |  |  |  |  |
|  | | | | | | | 4- Bad | | | | | | | | | | | | | | | | | | | | | | | | | | | | | | | | | |  | | | | | | |  | | | | | | | | | | | |  | | | | | | | | | | | |  | | | | | | | | | | | | | |  | | | | | | | | | | |  | | | | | | | | | | | | | | |  | | | | | | | | | | | | | | | | | | | |  | | | | | | | | | | | | | | |  | | | | | | | | | | | | | | |  | | | | | | | | | | | | | |  | | | | | | | | | | | | | |  | | | | | | | | |  | | | | | | | | | | | | | | |  | | | | | | | | | | | | | | | | | | | | |  | | | | | | | | | | | 3- Don´t know | | | | | | | | | | | | | | | | | | | | | | | | | | | | | | | | | | | | | | | | | | | | | | | | | | | | | | | | | | | |  | | | | | | | | | | | | | | | |  | | | | | | | | | | | | | |  | | | | | | | | | | | | |  | | | | | | | | | | | |  | | | | | | | | | | | | | | | | | | | |  | | | | | | | | | | | | | | | | | |  | | | | | | | | | | | | | | | | |  | | | | | | | | | | | | | | | |  | | | | | | | | | | | | | | | | | | | | | | | | | | | | | | | | | | |  | |  |  |  |  |
|  | | | | | | |  | | | | | | | | | |  | | | | | | | |  | | | | | | | | | | | | | | | |  | | | | | | |  | | | | | | | | | | | |  | | | | | | | | | | | |  | | | | | | | | | | | | | |  | | | | | | | | | | |  | | | | | | | | | | | | | | |  | | | | | | | | | | | | | | | | | | | |  | | | | | | | | | | | | | | |  | | | | | | | | | | | | | | |  | | | | | | | | | | | | | |  | | | | | | | | | | | | | |  | | | | | | | | |  | | | | | | | | | | | | | | |  | | | | | | | | | | | | | | | | | | | | |  | | | | | | | | | | |  | | | | | | | | | | | | | | | | | | |  | | | | | | | | | | | |  | | | | | | | | |  | | | | | | | | | | | | | | | | | | | |  | | | | | | | | | | | | | | | |  | | | | | | | | | | | | | |  | | | | | | | | | | | | |  | | | | | | | | | | | |  | | | | | | | | | | | | | | | | | | | |  | | | | | | | | | | | | | | | | | |  | | | | | | | | | | | | | | | | |  | | | | | | | | | | | | | | | |  | | | | | | | | | | | | | | | | | | | | | | | | | | | | | | | | | | |  | |  |  |  |  |
| **17- Do you think the sprayers are performing their tasks correctly ?** | | | | | | | | | | | | | | | | | | | | | | | | | | | | | | | | | | | | | | | | | | | | | | | | | | | | | | | | | | | | | | | | | | | | | | | | | | | | | | | | | | | | | | | | | | | | | | | | | | | | | | | | | | | | | | | | | | | | | | | | | | | | | | | | | | | | | | | | | | | | | | | | | | | | | | | | | | | | | | | | | | | | | | | | | | | | | | | | | | | | | | | | | | | | | | | |  | | | | | | | | | | | | | | | | |  | | | | | | | | | | | | | | | | | | | **18- Do you have knowledge on larvicides** | | | | | | | | | | | | | | | | | | | | | | | | | | | | | | | | | | | | | | | | | | | | | | | | | | | | | | | | | | | | | | | | | | | | | | | | | | | | | | | | | | | | | | | | | | | | | | | | | | | | | | | | | | | | | | | | | | | | | | | | | | | | | | | | | | | | | | | | | | | | | | | | | | | | | | | | | | | | | | | | | | | | | |  | | | | | | | | | | | | | | |  | | | | | | | | | | | | | | |  | | | | | | | | | | | | |  | | | | | | | |  |  |  |  | | |  | | | | | | | | | | | | | | |  | | |  |  |  |  |
|  | | 1- Yes | | | | | | | | | | | | | | | | | | | |  | | | | | | | | | | | | | | | | | | | | |  | | | | | | | |  | | | | | | | | | | | |  | | | | | | | | | | | | | | |  | | | | | | | | | | | | | |  | | | | | | | | | | | | | | |  | | | | | | | | | |  | | | | | | | | | | | | | | | | | | |  | | | | | | | | | | | | | | |  | | | | | | | | | | | | | | |  | | | | | | | | | | | | |  | | | | | | | | | | | | | | | | | | | | | | | | |  | | | | | | | | |  | | | | | | | | | | | | | |  | | | | | | | | | | | | | | | | | | | | |  | | | | | | | | | | | | | | | | 1- Yes | | | | | | | | | | | | | | | | | | | | | | | | | |  | | | | | | | | | | | | | |  | | | | | | | | | | | | | | | | |  | | | | | | | | | | | | | | | | |  | | | | | | | | | | | | |  | | | | | | | | | | | | | |  | | | | | | | | | | | | | | | |  | | | | | | | | | | | | | | | | | |  | | | | | | | | | | | | | | | | | | |  | | | | | | | | | | | | | | | |  | | | | | | | | | | | | | | | | | | |  | | | | | | | | | | | | | | | |  | | | |
|  | | 2- No | | | | | | | | | | | | | | | | | | | |  | | | | | | | | | | | | | | | | | | | | |  | | | | | | | |  | | | | | | | | | | | |  | | | | | | | | | | | | | | |  | | | | | | | | | | | | | |  | | | | | | | | | | | | | | |  | | | | | | | | | |  | | | | | | | | | | | | | | | | | | |  | | | | | | | | | | | | | | |  | | | | | | | | | | | | | | |  | | | | | | | | | | | | |  | | | | | | | | | | | | | | | | | | | | | | | | |  | | | | | | | | |  | | | | | | | | | | | | | |  | | | | | | | | | | | | | | | | | | | | |  | | | | | | | | | | | | | | | | 2- No | | | | | | | | | | | | | | | | | | | | | | | | | |  | | | | | | | | | | | | | |  | | | | | | | | | | | | | | | | |  | | | | | | | | | | | | | | | | |  | | | | | | | | | | | | |  | | | | | | | | | | | | | |  | | | | | | | | | | | | | | | |  | | | | | | | | | | | | | | | | | |  | | | | | | | | | | | | | | | | | | |  | | | | | | | | | | | | | | | |  | | | | | | | | | | | | | | | | | | |  | | | | | | | | | | | | | | | |  | | | |
|  | | 3- Don´t know | | | | | | | | | | | | | | | | | | | | | | | | | | | | | | | | | | | | | | | | | | | | | | | | |  | | | | | | | | | | | |  | | | | | | | | | | | | | | |  | | | | | | | | | | | | | |  | | | | | | | | | | | | | | |  | | | | | | | | | |  | | | | | | | | | | | | | | | | | | |  | | | | | | | | | | | | | | |  | | | | | | | | | | | | | | |  | | | | | | | | | | | | |  | | | | | | | | | | | | | | | | | | | | | | | | |  | | | | | | | | |  | | | | | | | | | | | | | |  | | | | | | | | | | | | | | | | | | | | |  | | | | | | | | | | | | | | | | 3- Don´t know | | | | | | | | | | | | | | | | | | | | | | | | | | | | | | | | | | | | | | | | | | | | | | | | | | | | | | | | |  | | | | | | | | | | | | | | | | |  | | | | | | | | | | | | |  | | | | | | | | | | | | | |  | | | | | | | | | | | | | | | |  | | | | | | | | | | | | | | | | | |  | | | | | | | | | | | | | | | | | | |  | | | | | | | | | | | | | | | |  | | | | | | | | | | | | | | | | | | |  | | | | | | | | | | | | | | | |  | | | |
|  | |  | | | | | | | | | | | | | | |  | | | | |  | | | | | | | | | | | | | | | | | | | | |  | | | | | | | |  | | | | | | | | | | | |  | | | | | | | | | | | | | | |  | | | | | | | | | | | | | |  | | | | | | | | | | | | | | |  | | | | | | | | | |  | | | | | | | | | | | | | | | | | | |  | | | | | | | | | | | | | | |  | | | | | | | | | | | | | | |  | | | | | | | | | | | | |  | | | | | | | | | | | | | | | | | | | | | | | | |  | | | | | | | | |  | | | | | | | | | | | | | |  | | | | | | | | | | | | | | | | | | | | |  | | | | | | | | | | | | | | | |  | | | | | | | | | | | | |  | | | | | | | | | | | | |  | | | | | | | | | | | | | |  | | | | | | | | | | | | | | | | |  | | | | | | | | | | | | | | | | |  | | | | | | | | | | | | |  | | | | | | | | | | | | | |  | | | | | | | | | | | | | | | |  | | | | | | | | | | | | | | | | | |  | | | | | | | | | | | | | | | | | | |  | | | | | | | | | | | | | | | |  | | | | | | | | | | | | | | | | | | |  | | | | | | | | | | | | | | | |  | | | |
| **19- Do you trust / believe in larvicides ?** | | | | | | | | | | | | | | | | | | | | | | | | | | | | | | | | | | | | | | | | | | | | | | | | | | | | | | | | | | | | | | | | | | | | | | | | | | | | | | | | | | | | | | | | | | | | | | | | | | | | | | | | | | | | | | | | | | | | |  | | | | | | | | | | | | | | | | | | |  | | | | | | | | | | | | | | |  | | | | | | | | | | | | | | |  | | | | | | | | | | | | |  | | | | | | | | | | | | | | | | | | | | | | | | |  | | | | | | | | | **20- Would you / did you grant access to the sprayers to treat water bodies close to your house ?** | | | | | | | | | | | | | | | | | | | | | | | | | | | | | | | | | | | | | | | | | | | | | | | | | | | | | | | | | | | | | | | | | | | | | | | | | | | | | | | | | | | | | | | | | | | | | | | | | | | | | | | | | | | | | | | | | | | | | | | | | | | | | | | | | | | | | | | | | | | | | | | | | | | | | | | | | | | | | | | | | | | | | | | | | | | | | | | | | | | | | | | | | | | | | | | | | | | | | | | | | | | | |  | | | | | | | | | | | | | | | |  | | | | | | | | | | | | | | | | | | |  | | | | | | | | | | | | | | | |  | | | |
|  | | 1- Yes | | | | | | | | | | | | | | | | | | | |  | | | | | | | | | | | | | | | | | | | | |  | | | | | | | |  | | | | | | | | | | | |  | | | | | | | | | | | | | | |  | | | | | | | | | | | | | |  | | | | | | | | | | | | | | |  | | | | | | | | | |  | | | | | | | | | | | | | | | | | | |  | | | | | | | | | | | | | | |  | | | | | | | | | | | | | | |  | | | | | | | | | | | | |  | | | | | | | | | | | | | | | | | | | | | | | | |  | | | | | | | | |  | | | | | | | | | | | | | | | | | | | | | | | | | | | | | | | | | | | | | | | | | | | | | | | | | | | | | | | | | | | | | | | | | | | | | | | | | | | | | | | | | | | | | | | | | | | | | | | | | | | | | | | | | | | | | | | | | | | | | | | | | | | | | | | | | | | | | | | | | | | | | | | | | | | | | | | | | | | | | | | | | | | | | | | | | | | | | | | | | | | | | | | | | | | | | | | | | | | | | | | | | | | | | | | | | | | | | | | | | | | | | | | | | | | | | | | | | | | | | | | | | | | | | | | | | | | | | | | |  | | | |
|  | | 2- No | | | | | | | | | | | | | | | | | | | |  | | | | | | | | | | | | | | | | | | | | |  | | | | | | | |  | | | | | | | | | | | |  | | | | | | | | | | | | | | |  | | | | | | | | | | | | | |  | | | | | | | | | | | | | | |  | | | | | | | | | |  | | | | | | | | | | | | | | | | | | |  | | | | | | | | | | | | | | |  | | | | | | | | | | | | | | |  | | | | | | | | | | | | |  | | | | | | | | | | | | | | | | | | | | | | | | |  | | | | | | | | |  | | | | | | | | | | | | | |  | | | | | | | | | | | | | | | | | | | | |  | | | | | | | | | | | | | | | | 1- Yes | | | | | | | | | | | | | | | | | | | | | | | | | |  | | | | | | | | | | | | | |  | | | | | | | | | | | | | | | | |  | | | | | | | | | | | | | | | | |  | | | | | | | | | | | | |  | | | | | | | | | | | | | |  | | | | | | | | | | | | | | | |  | | | | | | | | | | | | | | | | | |  | | | | | | | | | | | | | | | | | | |  | | | | | | | | | | | | | | | |  | | | | | | | | | | | | | | | | | | |  | | | | | | | | | | | | | | | |  | | | |
|  | | 3- Don´t know | | | | | | | | | | | | | | | | | | | | | | | | | | | | | | | | | | | | | | | | | | | | | | | | |  | | | | | | | | | | | |  | | | | | | | | | | | | | | |  | | | | | | | | | | | | | |  | | | | | | | | | | | | | | |  | | | | | | | | | |  | | | | | | | | | | | | | | | | | | |  | | | | | | | | | | | | | | |  | | | | | | | | | | | | | | |  | | | | | | | | | | | | |  | | | | | | | | | | | | | | | | | | | | | | | | |  | | | | | | | | |  | | | | | | | | | | | | | |  | | | | | | | | | | | | | | | | | | | | |  | | | | | | | | | | | | | | | | 2- No | | | | | | | | | | | | | | | | | | | | | | | | | |  | | | | | | | | | | | | | |  | | | | | | | | | | | | | | | | |  | | | | | | | | | | | | | | | | |  | | | | | | | | | | | | |  | | | | | | | | | | | | | |  | | | | | | | | | | | | | | | |  | | | | | | | | | | | | | | | | | |  | | | | | | | | | | | | | | | | | | |  | | | | | | | | | | | | | | | |  | | | | | | | | | | | | | | | | | | |  | | | | | | | | | | | | | | | |  | | | |
|  | |  | | | | | | | | | | | | | | |  | | | | |  | | | | | | | | | | | | | | | | | | | | |  | | | | | | | |  | | | | | | | | | | | |  | | | | | | | | | | | | | | |  | | | | | | | | | | | | | |  | | | | | | | | | | | | | | |  | | | | | | | | | |  | | | | | | | | | | | | | | | | | | |  | | | | | | | | | | | | | | |  | | | | | | | | | | | | | | |  | | | | | | | | | | | | |  | | | | | | | | | | | | | | | | | | | | | | | | |  | | | | | | | | |  | | | | | | | | | | | | | |  | | | | | | | | | | | | | | | | | | | | |  | | | | | | | | | | | | | | | | 3- Don´t know | | | | | | | | | | | | | | | | | | | | | | | | | | | | | | | | | | | | | | | | | | | | | | | | | | | | | | | | |  | | | | | | | | | | | | | | | | |  | | | | | | | | | | | | |  | | | | | | | | | | | | | |  | | | | | | | | | | | | | | | |  | | | | | | | | | | | | | | | | | |  | | | | | | | | | | | | | | | | | | |  | | | | | | | | | | | | | | | |  | | | | | | | | | | | | | | | | | | |  | | | | | | | | | | | | | | | |  | | | |
| **21- Do you think that larvicide application will reduce the risk for infection with malaria ?** | | | | | | | | | | | | | | | | | | | | | | | | | | | | | | | | | | | | | | | | | | | | | | | | | | | | | | | | | | | | | | | | | | | | | | | | | | | | | | | | | | | | | | | | | | | | | | | | | | | | | | | | | | | | | | | | | | | | | | | | | | | | | | | | | | | | | | | | | | | | | | | | | | | | | | | | | | | | | | | | | | | | | | | | | | | | | | | | | | | | | | | | | | | | | | | | | | | | | | | | | | | |  | | | | | | | | |  | | | | | | | | | | | | | |  | | | | | | | | | | | | | | | | | | | | |  | | | | | | | | | | | | | | | |  | | | | | | | | | | | | |  | | | | | | | | | | | | |  | | | | | | | | | | | | | |  | | | | | | | | | | | | | | | | |  | | | | | | | | | | | | | | | | |  | | | | | | | | | | | | |  | | | | | | | | | | | | | |  | | | | | | | | | | | | | | | |  | | | | | | | | | | | | | | | | | |  | | | | | | | | | | | | | | | | | | |  | | | | | | | | | | | | | | | |  | | | | | | | | | | | | | | | | | | |  | | | | | | | | | | | | | | | |  | | | |
|  | |  | | | | | | | | | | | | | | |  | | | | |  | | | | | | | | | | | | | | | | | | | | | | | | | | | | | | | | | | | | | | | | | | | | | | | | | | | | | | | | | | | | | | | | | | | | | | | | | | | | | | | | | | | | | | | | | | | | | | | | | | | | | | | | | | | | | | | | | | | | | | | | | | | | | | | | |  | | | | | | | | | | | | | | |  | | | | | | | | | | | | | **22- Do you trust that the larvicide is inoffensive against humans and animals ?** | | | | | | | | | | | | | | | | | | | | | | | | | | | | | | | | | | | | | | | | | | | | | | | | | | | | | | | | | | | | | | | | | | | | | | | | | | | | | | | | | | | | | | | | | | | | | | | | | | | | | | | | | | | | | | | | | | | | | | | | | | | | | | | | | | | | | | | | | | | | | | | | | | | | | | | | | | | | | | | | | | | | | | | | | | | | | | | | | | | | | | | | | | | | | | | | | | | | | | | | | | | | | | | | | | | | | | | | | | | | | | | | | | | | | | | | | | | | | | | | | | | | | | | | | | | | | | | | | | | | | | | | | | | | | | | |  | | | | | | | | | | | | | | | | | |  | | | |
|  | | 1- Yes | | | | | | | | | | | | | | | | | | | |  | | | | | | | | | | | | | | | | | | | | |  | | | | | | | |  | | | | | | | | | | | |  | | | | | | | | | | | | | | |  | | | | | | | | | | | | | |  | | | | | | | | | | | | | | |  | | | | | | | | | |  | | | | | | | | | | | | | | | | | | |  | | | | | | | | | | | | | | |  | | | | | | | | | | | | | | |  | | | | | | | | | | | | |  | | | | | | | | | | | | | | | | | | | | | | | | |  | | | | | | | | |  | | | | | | | | | | | | | |  | | | | | | | | | | | | | | | | | | | | |  | | | | | | | | | | | | | | | | 1 | | | | | | | | | | | | | Very probable | | | | | | | | | | | | | | | | | | | | | | | | | | | | | | | | | | | | | | | | | | | | | | | | | | | | | | | | | | | | |  | | | | | | | | | | | | |  | | | | | | | | | | | | | |  | | | | | | | | | | | | | | | |  | | | | | | | | | | | | | | | | | |  | | | | | | | | | | | | | | | | | | |  | | | | | | | | | | | | | | | |  | | | | | | | | | | | | | | | | | | |  | | | | | | | | | | | | | | | |  | | | |
|  | | 2- No | | | | | | | | | | | | | | | | | | | |  | | | | | | | | | | | | | | | | | | | | |  | | | | | | | |  | | | | | | | | | | | |  | | | | | | | | | | | | | | |  | | | | | | | | | | | | | |  | | | | | | | | | | | | | | |  | | | | | | | | | |  | | | | | | | | | | | | | | | | | | |  | | | | | | | | | | | | | | |  | | | | | | | | | | | | | | |  | | | | | | | | | | | | |  | | | | | | | | | | | | | | | | | | | | | | | | |  | | | | | | | | |  | | | | | | | | | | | | | |  | | | | | | | | | | | | | | | | | | | | |  | | | | | | | | | | | | | | | | 2 | | | | | | | | | | | | | Probable | | | | | | | | | | | | | | | | | | | | | | | | | | | | | | | | | | | | | | | | | | | |  | | | | | | | | | | | | | | | | |  | | | | | | | | | | | | |  | | | | | | | | | | | | | |  | | | | | | | | | | | | | | | |  | | | | | | | | | | | | | | | | | |  | | | | | | | | | | | | | | | | | | |  | | | | | | | | | | | | | | | |  | | | | | | | | | | | | | | | | | | |  | | | | | | | | | | | | | | | |  | | | |
|  | | 3- Don´t know | | | | | | | | | | | | | | | | | | | | | | | | | | | | | | | | | | | | | | | | | | | | | | | | |  | | | | | | | | | | | |  | | | | | | | | | | | | | | |  | | | | | | | | | | | | | |  | | | | | | | | | | | | | | |  | | | | | | | | | |  | | | | | | | | | | | | | | | | | | |  | | | | | | | | | | | | | | |  | | | | | | | | | | | | | | |  | | | | | | | | | | | | |  | | | | | | | | | | | | | | | | | | | | | | | | |  | | | | | | | | |  | | | | | | | | | | | | | |  | | | | | | | | | | | | | | | | | | | | |  | | | | | | | | | | | | | | | | 3 | | | | | | | | | | | | | Neutral | | | | | | | | | | | | | | | | | | | | | | | | | | | | | | | | | | | | | | | | | | | |  | | | | | | | | | | | | | | | | |  | | | | | | | | | | | | |  | | | | | | | | | | | | | |  | | | | | | | | | | | | | | | |  | | | | | | | | | | | | | | | | | |  | | | | | | | | | | | | | | | | | | |  | | | | | | | | | | | | | | | |  | | | | | | | | | | | | | | | | | | |  | | | | | | | | | | | | | | | |  | | | |
|  | |  | | | | | | | | | | | | | | |  | | | | |  | | | | | | | | | | | | | | | | | | | | |  | | | | | | | |  | | | | | | | | | | | |  | | | | | | | | | | | | | | |  | | | | | | | | | | | | | |  | | | | | | | | | | | | | | |  | | | | | | | | | |  | | | | | | | | | | | | | | | | | | |  | | | | | | | | | | | | | | |  | | | | | | | | | | | | | | |  | | | | | | | | | | | | |  | | | | | | | | | | | | | | | | | | | | | | | | |  | | | | | | | | |  | | | | | | | | | | | | | |  | | | | | | | | | | | | | | | | | | | | |  | | | | | | | | | | | | | | | | 4 | | | | | | | | | | | | | Not probable | | | | | | | | | | | | | | | | | | | | | | | | | | | | | | | | | | | | | | | | | | | | | | | | | | | | | | | | | | | | |  | | | | | | | | | | | | |  | | | | | | | | | | | | | |  | | | | | | | | | | | | | | | |  | | | | | | | | | | | | | | | | | |  | | | | | | | | | | | | | | | | | | |  | | | | | | | | | | | | | | | |  | | | | | | | | | | | | | | | | | | |  | | | | | | | | | | | | | | | |  | | | |
|  | |  | | | | | | | | | | | | | | |  | | | | |  | | | | | | | | | | | | | | | | | | | | |  | | | | | | | |  | | | | | | | | | | | |  | | | | | | | | | | | | | | |  | | | | | | | | | | | | | |  | | | | | | | | | | | | | | |  | | | | | | | | | |  | | | | | | | | | | | | | | | | | | |  | | | | | | | | | | | | | | |  | | | | | | | | | | | | | | |  | | | | | | | | | | | | |  | | | | | | | | | | | | | | | | | | | | | | | | |  | | | | | | | | |  | | | | | | | | | | | | | |  | | | | | | | | | | | | | | | | | | | | |  | | | | | | | | | | | | | | | | 5 | | | | | | | | | | | | | Very improbable | | | | | | | | | | | | | | | | | | | | | | | | | | | | | | | | | | | | | | | | | | | | | | | | | | | | | | | | | | | | |  | | | | | | | | | | | | |  | | | | | | | | | | | | | |  | | | | | | | | | | | | | | | |  | | | | | | | | | | | | | | | | | |  | | | | | | | | | | | | | | | | | | |  | | | | | | | | | | | | | | | |  | | | | | | | | | | | | | | | | | | |  | | | | | | | | | | | | | | | |  | | | |
|  | |  | | | | | | | | | | | | | | |  | | | | |  | | | | | | | | | | | | | | | | | | | | |  | | | | | | | |  | | | | | | | | | | | |  | | | | | | | | | | | | | | |  | | | | | | | | | | | | | |  | | | | | | | | | | | | | | |  | | | | | | | | | |  | | | | | | | | | | | | | | | | | | |  | | | | | | | | | | | | | | |  | | | | | | | | | | | | | | |  | | | | | | | | | | | | |  | | | | | | | | | | | | | | | | | | | | | | | | |  | | | | | | | | |  | | | | | | | | | | | | | |  | | | | | | | | | | | | | | | | | | | | |  | | | | | | | | | | | | | | | |  | | | | | | | | | | | | |  | | | | | | | | | | | | |  | | | | | | | | | | | | | |  | | | | | | | | | | | | | | | | |  | | | | | | | | | | | | | | | | |  | | | | | | | | | | | | |  | | | | | | | | | | | | | |  | | | | | | | | | | | | | | | |  | | | | | | | | | | | | | | | | | |  | | | | | | | | | | | | | | | | | | |  | | | | | | | | | | | | | | | |  | | | | | | | | | | | | | | | | | | |  | | | | | | | | | | | | | | | |  | | | |
| **23- Are there mosquitoes in your village / sector?** | | | | | | | | | | | | | | | | | | | | | | | | | | | | | | | | | | | | | | | | | | | | | | | | | | | | | | | | | | | | | | | | | | | | | | | | | | | | | | | | | | | | | | | | | | | | | | | | | | | | | | | | | | | | | | | | | | | | | | | | | | | | | | | | | | | | | | | | | | | | | | | | | | | | | | | | | | | | | | | | | | | | | | **24- How do you perceive mosquito abundance compared to the preceding years ?** | | | | | | | | | | | | | | | | | | | | | | | | | | | | | | | | | | | | | | | | | | | | | | | | | | | | | | | | | | | | | | | | | | | | | | | | | | | | | | | | | | | | | | | | | | | | | | | | | | | | | | | | | | | | | | | | | | | | | | | | | | | | | | | | | | | | | | | | | | | | | | | | | | | | | | | | | | | | | | | | | | | | | | | | | | | | | | | | | | | | | | | | | | | | | | | | | | | | | | | | | | | | | | | | | | | | | | | | | | | | | | | | | | | | | | | | | | | | | | | | | | | | | | | | | | | | | | | | | | | | | | | | | | | | | | | | | | | | | | | | | | | | | | | | | | | | | | | | | | | | | | |  | | | |
|  | | 1- Yes | | | | | | | | | | | | | | | | | | | |  | | | | | | | | | | | | | | | | | | | | |  | | | | | | | |  | | | | | | | | | | | |  | | | | | | | | | | | | | | |  | | | | | | | | | | | | | |  | | | | | | | | | | | | | | |  | | | | | | | | | |  | | | | | | | | | | | | | | | | | | |  | | | | | | | | | | | | | | |  | | | | | | | | | | | | | | |  | | | | | | | | | | | | |  | | | | | | | | | | | | | | | | | | | | | | | | |  | | | | | | | | |  | | | | | | | | | | | | | |  | | | | | | | | | | | | | | | | | | | | |  | | | | | | | | | | | | | | | | 1 | | | | | | | | | | | | | Much less | | | | | | | | | | | | | | | | | | | | | | | | | | | | | | | | | | | | | | | | | | | | | | | | | | | | | | | | | | | | |  | | | | | | | | | | | | |  | | | | | | | | | | | | | |  | | | | | | | | | | | | | | | |  | | | | | | | | | | | | | | | | | |  | | | | | | | | | | | | | | | | | | |  | | | | | | | | | | | | | | | |  | | | | | | | | | | | | | | | | | | |  | | | | | | | | | | | | | | | |  | | | |
|  | | 2- No | | | | | | | | | | | | | | | | | | | |  | | | | | | | | | | | | | | | | | | | | |  | | | | | | | |  | | | | | | | | | | | |  | | | | | | | | | | | | | | |  | | | | | | | | | | | | | |  | | | | | | | | | | | | | | |  | | | | | | | | | |  | | | | | | | | | | | | | | | | | | |  | | | | | | | | | | | | | | |  | | | | | | | | | | | | | | |  | | | | | | | | | | | | |  | | | | | | | | | | | | | | | | | | | | | | | | |  | | | | | | | | |  | | | | | | | | | | | | | |  | | | | | | | | | | | | | | | | | | | | |  | | | | | | | | | | | | | | | | 2 | | | | | | | | | | | | | Less | | | | | | | | | | | | | | | | | | | | | | | | | | |  | | | | | | | | | | | | | | | | |  | | | | | | | | | | | | | | | | |  | | | | | | | | | | | | |  | | | | | | | | | | | | | |  | | | | | | | | | | | | | | | |  | | | | | | | | | | | | | | | | | |  | | | | | | | | | | | | | | | | | | |  | | | | | | | | | | | | | | | |  | | | | | | | | | | | | | | | | | | |  | | | | | | | | | | | | | | | |  | | | |
|  | | 3- Don´t know | | | | | | | | | | | | | | | | | | | | | | | | | | | | | | | | | | | | | | | | | | | | | | | | |  | | | | | | | | | | | |  | | | | | | | | | | | | | | |  | | | | | | | | | | | | | |  | | | | | | | | | | | | | | |  | | | | | | | | | |  | | | | | | | | | | | | | | | | | | |  | | | | | | | | | | | | | | |  | | | | | | | | | | | | | | |  | | | | | | | | | | | | |  | | | | | | | | | | | | | | | | | | | | | | | | |  | | | | | | | | |  | | | | | | | | | | | | | |  | | | | | | | | | | | | | | | | | | | | |  | | | | | | | | | | | | | | | | 3 | | | | | | | | | | | | | No change | | | | | | | | | | | | | | | | | | | | | | | | | | | | | | | | | | | | | | | | | | | | | | | | | | | | | | | | | | | | | | | | | | | | | | | | | |  | | | | | | | | | | | | | |  | | | | | | | | | | | | | | | |  | | | | | | | | | | | | | | | | | |  | | | | | | | | | | | | | | | | | | |  | | | | | | | | | | | | | | | |  | | | | | | | | | | | | | | | | | | |  | | | | | | | | | | | | | | | |  | | | |
|  | |  | | | | | | | | | | | | | | |  | | | | |  | | | | | | | | | | | | | | | | | | | | |  | | | | | | | |  | | | | | | | | | | | |  | | | | | | | | | | | | | | |  | | | | | | | | | | | | | |  | | | | | | | | | | | | | | |  | | | | | | | | | |  | | | | | | | | | | | | | | | | | | |  | | | | | | | | | | | | | | |  | | | | | | | | | | | | | | |  | | | | | | | | | | | | |  | | | | | | | | | | | | | | | | | | | | | | | | |  | | | | | | | | |  | | | | | | | | | | | | | |  | | | | | | | | | | | | | | | | | | | | |  | | | | | | | | | | | | | | | | 4 | | | | | | | | | | | | | More | | | | | | | | | | | | | | | | | | | | | | | | | | |  | | | | | | | | | | | | | | | | |  | | | | | | | | | | | | | | | | |  | | | | | | | | | | | | |  | | | | | | | | | | | | | |  | | | | | | | | | | | | | | | |  | | | | | | | | | | | | | | | | | |  | | | | | | | | | | | | | | | | | | |  | | | | | | | | | | | | | | | |  | | | | | | | | | | | | | | | | | | |  | | | | | | | | | | | | | | | |  | | | |
|  | |  | | | | | | | | | | | | | | |  | | | | |  | | | | | | | | | | | | | | | | | | | | |  | | | | | | | |  | | | | | | | | | | | |  | | | | | | | | | | | | | | |  | | | | | | | | | | | | | |  | | | | | | | | | | | | | | |  | | | | | | | | | |  | | | | | | | | | | | | | | | | | | |  | | | | | | | | | | | | | | |  | | | | | | | | | | | | | | |  | | | | | | | | | | | | |  | | | | | | | | | | | | | | | | | | | | | | | | |  | | | | | | | | |  | | | | | | | | | | | | | |  | | | | | | | | | | | | | | | | | | | | |  | | | | | | | | | | | | | | | | 5 | | | | | | | | | | | | | Much more | | | | | | | | | | | | | | | | | | | | | | | | | | | | | | | | | | | | | | | | | | | | | | | | | | | | | | | | | | | | |  | | | | | | | | | | | | |  | | | | | | | | | | | | | |  | | | | | | | | | | | | | | | |  | | | | | | | | | | | | | | | | | |  | | | | | | | | | | | | | | | | | | |  | | | | | | | | | | | | | | | |  | | | | | | | | | | | | | | | | | | |  | | | | | | | | | | | | | | | |  | | | |
| **25- How do you perceive the number of malaria cases compared to the last years ?** | | | | | | | | | | | | | | | | | | | | | | | | | | | | | | | | | | | | | | | | | | | | | | | | | | | | | | | | | | | | | | | | | | | | | | | | | | | | | | | | | | | | | | | | | | | | | | | | | | | | | | | | | | | | | | | | | | | | | | | | | | | | | | | | | | | | | | | | | | | | | | | | | | | | | | | | | | | | | | | | | | | | | | | | | | | | | | | | | | | | | | | | | | | | | | | | | | | | | | | | | | | | | | | | | | | | | | | | | | | | | | | | | | | | | | | | | | | | | | | | | | | | | | **26- Would you be willing to financially contribute to such a project and with how much per year?** | | | | | | | | | | | | | | | | | | | | | | | | | | | | | | | | | | | | | | | | | | | | | | | | | | | | | | | | | | | | | | | | | | | | | | | | | | | | | | | | | | | | | | | | | | | | | | | | | | | | | | | | | | | | | | | | | | | | | | | | | | | | | | | | | | | | | | | | | | | | | | | | | | | | | | | | | | | | | | | | | | | | | | | | | | | | | | | | | | | | | | | | | | | | | | | | | | | | | | | | | | | | |  | | | | | | | | | | | | | | | | | |  | | | |
|  | | 1 | | | | | | | | | | | | | | | Much less | | | | | | | | | | | | | | | | | | | | | | | | | | | | | | | | | | | | | | | | | | | | | |  | | | | | | | | | | | | | | |  | | | | | | | | | | | | | |  | | | | | | | | | | | | | | |  | | | | | | | | | |  | | | | | | | | | | | | | | | | | | |  | | | | | | | | | | | | | | |  | | | | | | | | | | | | | | |  | | | | | | | | | | | | |  | | | | | | | | | | | | | | | | | | | | | | | | |  | | | | | | | | |  | | | | | | | | | | | | | |  | | | | | | | | | | | | | | | | | | | | |  | | | | | | | | | | | | | | | |  | | | | | | | | | | | | | | | | | | | | | | | | | | | | | | | | | | | | | | | | | | | | | | | | | | | | | | | | | | | | | | | | | | | | | | | | | | | | | | | | | | | | | | | | | | | | | | | | | | | | | | | | | | | | | | | | | | | | | | | | | | | | | | | | | | | | | | | | | | | | | | | | | | | | | | | | | | | | | | | | | | | | | | | | | | | | | | | | | | | | | | | | | | | | |  | | | | | | | | | | | | | | | |  | | | |
|  | | 2 | | | | | | | | | | | | | | | Less | | | | | | | | | | | | | | | | | | | | | | | | | |  | | | | | | | |  | | | | | | | | | | | |  | | | | | | | | | | | | | | |  | | | | | | | | | | | | | |  | | | | | | | | | | | | | | |  | | | | | | | | | |  | | | | | | | | | | | | | | | | | | |  | | | | | | | | | | | | | | |  | | | | | | | | | | | | | | |  | | | | | | | | | | | | |  | | | | | | | | | | | | | | | | | | | | | | | | |  | | | | | | | | |  | | | | | | | | | | | | | |  | | | | | | | | | | | | | | | | | | | | |  | | | | | | | | | | | | | | | | 1 | | | | | | | | | | | | | 100 FCFA | | | | | | | | | | | | | | | | | | | | | | | | | | | | | | | | | | | | | | | | | | | |  | | | | | | | | | | | | | | | | |  | | | | | | | | | | | | |  | | | | | | | | | | | | | |  | | | | | | | | | | | | | | | |  | | | | | | | | | | | | | | | | | |  | | | | | | | | | | | | | | | | | | |  | | | | | | | | | | | | | | | |  | | | | | | | | | | | | | | | | | | |  | | | | | | | | | | | | | | | |  | | | |
|  | | 3 | | | | | | | | | | | | | | | No change | | | | | | | | | | | | | | | | | | | | | | | | | | | | | | | | | | | | | | | | | | | | | | | | | | | | | | | | | | | | |  | | | | | | | | | | | | | |  | | | | | | | | | | | | | | |  | | | | | | | | | |  | | | | | | | | | | | | | | | | | | |  | | | | | | | | | | | | | | |  | | | | | | | | | | | | | | |  | | | | | | | | | | | | |  | | | | | | | | | | | | | | | | | | | | | | | | |  | | | | | | | | |  | | | | | | | | | | | | | |  | | | | | | | | | | | | | | | | | | | | |  | | | | | | | | | | | | | | | | 2 | | | | | | | | | | | | | 200 FCFA | | | | | | | | | | | | | | | | | | | | | | | | | | | | | | | | | | | | | | | | | | | |  | | | | | | | | | | | | | | | | |  | | | | | | | | | | | | |  | | | | | | | | | | | | | |  | | | | | | | | | | | | | | | |  | | | | | | | | | | | | | | | | | |  | | | | | | | | | | | | | | | | | | |  | | | | | | | | | | | | | | | |  | | | | | | | | | | | | | | | | | | |  | | | | | | | | | | | | | | | |  | | | |
|  | | 4 | | | | | | | | | | | | | | | More | | | | | | | | | | | | | | | | | | | | | | | | | |  | | | | | | | |  | | | | | | | | | | | |  | | | | | | | | | | | | | | |  | | | | | | | | | | | | | |  | | | | | | | | | | | | | | |  | | | | | | | | | |  | | | | | | | | | | | | | | | | | | |  | | | | | | | | | | | | | | |  | | | | | | | | | | | | | | |  | | | | | | | | | | | | |  | | | | | | | | | | | | | | | | | | | | | | | | |  | | | | | | | | |  | | | | | | | | | | | | | |  | | | | | | | | | | | | | | | | | | | | |  | | | | | | | | | | | | | | | | 3 | | | | | | | | | | | | | 500 FCFA | | | | | | | | | | | | | | | | | | | | | | | | | | | | | | | | | | | | | | | | | | | |  | | | | | | | | | | | | | | | | |  | | | | | | | | | | | | |  | | | | | | | | | | | | | |  | | | | | | | | | | | | | | | |  | | | | | | | | | | | | | | | | | |  | | | | | | | | | | | | | | | | | | |  | | | | | | | | | | | | | | | |  | | | | | | | | | | | | | | | | | | |  | | | | | | | | | | | | | | | |  | | | |
|  | | 5 | | | | | | | | | | | | | | | Much more | | | | | | | | | | | | | | | | | | | | | | | | | | | | | | | | | | | | | | | | | | | | | |  | | | | | | | | | | | | | | |  | | | | | | | | | | | | | |  | | | | | | | | | | | | | | |  | | | | | | | | | |  | | | | | | | | | | | | | | | | | | |  | | | | | | | | | | | | | | |  | | | | | | | | | | | | | | |  | | | | | | | | | | | | |  | | | | | | | | | | | | | | | | | | | | | | | | |  | | | | | | | | |  | | | | | | | | | | | | | |  | | | | | | | | | | | | | | | | | | | | |  | | | | | | | | | | | | | | | | 4 | | | | | | | | | | | | | 800 FCFA | | | | | | | | | | | | | | | | | | | | | | | | | | | | | | | | | | | | | | | | | | | |  | | | | | | | | | | | | | | | | |  | | | | | | | | | | | | |  | | | | | | | | | | | | | |  | | | | | | | | | | | | | | | |  | | | | | | | | | | | | | | | | | |  | | | | | | | | | | | | | | | | | | |  | | | | | | | | | | | | | | | |  | | | | | | | | | | | | | | | | | | |  | | | | | | | | | | | | | | | |  | | | |
|  | |  | | | | | | | | | | | | | | |  | | | | |  | | | | | | | | | | | | | | | | | | | | |  | | | | | | | |  | | | | | | | | | | | |  | | | | | | | | | | | | | | |  | | | | | | | | | | | | | |  | | | | | | | | | | | | | | |  | | | | | | | | | |  | | | | | | | | | | | | | | | | | | |  | | | | | | | | | | | | | | |  | | | | | | | | | | | | | | |  | | | | | | | | | | | | |  | | | | | | | | | | | | | | | | | | | | | | | | |  | | | | | | | | |  | | | | | | | | | | | | | |  | | | | | | | | | | | | | | | | | | | | |  | | | | | | | | | | | | | | | | 5 | | | | | | | | | | | | | 1000FCFA | | | | | | | | | | | | | | | | | | | | | | | | | | | | | | | | | | | | | | | | | | | |  | | | | | | | | | | | | | | | | |  | | | | | | | | | | | | |  | | | | | | | | | | | | | |  | | | | | | | | | | | | | | | |  | | | | | | | | | | | | | | | | | |  | | | | | | | | | | | | | | | | | | |  | | | | | | | | | | | | | | | |  | | | | | | | | | | | | | | | | | | |  | | | | | | | | | | | | | | | |  | | | |
|  | |  | | | | | | | | | | | | | | |  | | | | |  | | | | | | | | | | | | | | | | | | | | |  | | | | | | | |  | | | | | | | | | | | |  | | | | | | | | | | | | | | |  | | | | | | | | | | | | | |  | | | | | | | | | | | | | | |  | | | | | | | | | |  | | | | | | | | | | | | | | | | | | |  | | | | | | | | | | | | | | |  | | | | | | | | | | | | | | |  | | | | | | | | | | | | |  | | | | | | | | | | | | | | | | | | | | | | | | |  | | | | | | | | |  | | | | | | | | | | | | | |  | | | | | | | | | | | | | | | | | | | | |  | | | | | | | | | | | | | | | |  | | | | | | | | | | | | |  | | | | | | | | | | | | |  | | | | | | | | | | | | | |  | | | | | | | | | | | | | | | | |  | | | | | | | | | | | | | | | | |  | | | | | | | | | | | | |  | | | | | | | | | | | | | |  | | | | | | | | | | | | | | | |  | | | | | | | | | | | | | | | | | |  | | | | | | | | | | | | | | | | | | |  | | | | | | | | | | | | | | | |  | | | | | | | | | | | | | | | | | | |  | | | | | | | | | | | | | | | |  | | | |
| **27- Would you be willing to personally participate in spraying activities** | | | | | | | | | | | | | | | | | | | | | | | | | | | | | | | | | | | | | | | | | | | | | | | | | | | | | | | | | | | | | | | | | | | | | | | | | | | | | | | | | | | | | | | | | | | | | | | | | | | | | | | | | | | | | | | | | | | | | | | | | | | | | | | | | | | | | | | | | | | | | | | | | | | | | | | | | | | | | | | | | | | | | | | | | | | | | | | | | | |  | | | | | | | | | | | | | | | | | | | | | | | | |  | | | | | | | | |  | | | | | | | | | | | | | |  | | | | | | | | | | | | | | | | | | | | |  | | | | | | | | | | | | | | | |  | | | | | | | | | | | | |  | | | | | | | | | | | | |  | | | | | | | | | | | | | |  | | | | | | | | | | | | | | | | |  | | | | | | | | | | | | | | | | |  | | | | | | | | | | | | |  | | | | | | | | | | | | | |  | | | | | | | | | | | | | | | |  | | | | | | | | | | | | | | | | | |  | | | | | | | | | | | | | | | | | | |  | | | | | | | | | | | | | | | |  | | | | | | | | | | | | | | | | | | |  | | | | | | | | | | | | | | | |  | | | |
|  | | 1- Yes | | | | | | | | | | | | | | | | | | | |  | | | | | | | | | | | | | | | | | | | | |  | | | | | | | |  | | | | | | | | | | | |  | | | | | | | | | | | | | | |  | | | | | | | | | | | | | |  | | | | | | | | | | | | | | |  | | | | | | | | | |  | | | | | | | | | | | | | | | | | | |  | | | | | | | | | | | | | | |  | | | | | | | | | | | | | | |  | | | | | | | | | | | | |  | | | | | | | | | | | | | | | | | | | | | | | | |  | | | | | | | | |  | | | | | | | | | | | | | |  | | | | | | | | | | | | | | | | | | | | |  | | | | | | | | | | | | | | | |  | | | | | | | | | | | | |  | | | | | | | | | | | | |  | | | | | | | | | | | | | |  | | | | | | | | | | | | | | | | |  | | | | | | | | | | | | | | | | |  | | | | | | | | | | | | |  | | | | | | | | | | | | | |  | | | | | | | | | | | | | | | |  | | | | | | | | | | | | | | | | | |  | | | | | | | | | | | | | | | | | | |  | | | | | | | | | | | | | | | |  | | | | | | | | | | | | | | | | | | |  | | | | | | | | | | | | | | | |  | | | |
|  | | 2- No | | | | | | | | | | | | | | | | | | | |  | | | | | | | | | | | | | | | | | | | | |  | | | | | | | |  | | | | | | | | | | | |  | | | | | | | | | | | | | | |  | | | | | | | | | | | | | |  | | | | | | | | | | | | | | |  | | | | | | | | | |  | | | | | | | | | | | | | | | | | | |  | | | | | | | | | | | | | | |  | | | | | | | | | | | | | | |  | | | | | | | | | | | | |  | | | | | | | | | | | | | | | | | | | | | | | | |  | | | | | | | | |  | | | | | | | | | | | | | |  | | | | | | | | | | | | | | | | | | | | |  | | | | | | | | | | | | | | | |  | | | | | | | | | | | | |  | | | | | | | | | | | | |  | | | | | | | | | | | | | |  | | | | | | | | | | | | | | | | |  | | | | | | | | | | | | | | | | |  | | | | | | | | | | | | |  | | | | | | | | | | | | | |  | | | | | | | | | | | | | | | |  | | | | | | | | | | | | | | | | | |  | | | | | | | | | | | | | | | | | | |  | | | | | | | | | | | | | | | |  | | | | | | | | | | | | | | | | | | |  | | | | | | | | | | | | | | | |  | | | |
|  | | 3-Depends on payment | | | | | | | | | | | | | | | | | | | | | | | | | | | | | | | | | | | | | | | | | | | | | | | | | | | | | | | | | | | | | | | | | | | | | | | | | | | |  | | | | | | | | | | | | | |  | | | | | | | | | | | | | | |  | | | | | | | | | |  | | | | | | | | | | | | | | | | | | |  | | | | | | | | | | | | | | |  | | | | | | | | | | | | | | |  | | | | | | | | | | | | |  | | | | | | | | | | | | | | | | | | | | | | | | |  | | | | | | | | |  | | | | | | | | | | | | | |  | | | | | | | | | | | | | | | | | | | | |  | | | | | | | | | | | | | | | |  | | | | | | | | | | | | |  | | | | | | | | | | | | |  | | | | | | | | | | | | | |  | | | | | | | | | | | | | | | | |  | | | | | | | | | | | | | | | | |  | | | | | | | | | | | | |  | | | | | | | | | | | | | |  | | | | | | | | | | | | | | | |  | | | | | | | | | | | | | | | | | |  | | | | | | | | | | | | | | | | | | |  | | | | | | | | | | | | | | | |  | | | | | | | | | | | | | | | | | | |  | | | | | | | | | | | | | | | |  | | | |
|  | |  | | | | | | | | | | | | | | |  | | | | |  | | | | | | | | | | | | | | | | | | | | |  | | | | | | | |  | | | | | | | | | | | |  | | | | | | | | | | | | | | |  | | | | | | | | | | | | | |  | | | | | | | | | | | | | | |  | | | | | | | | | |  | | | | | | | | | | | | | | | | | | |  | | | | | | | | | | | | | | |  | | | | | | | | | | | | | | |  | | | | | | | | | | | | |  | | | | | | | | | | | | | | | | | | | | | | | | |  | | | | | | | | |  | | | | | | | | | | | | | |  | | | | | | | | | | | | | | | | | | | | |  | | | | | | | | | | | | | | | |  | | | | | | | | | | | | |  | | | | | | | | | | | | |  | | | | | | | | | | | | | |  | | | | | | | | | | | | | | | | |  | | | | | | | | | | | | | | | | |  | | | | | | | | | | | | |  | | | | | | | | | | | | | |  | | | | | | | | | | | | | | | |  | | | | | | | | | | | | | | | | | |  | | | | | | | | | | | | | | | | | | |  | | | | | | | | | | | | | | | |  | | | | | | | | | | | | | | | | | | |  | | | | | | | | | | | | | | | |  | | | |
| **Observations, perceptions during the larviciding campaign** | | | | | | | | | | | | | | | | | | | | | | | | | | | | | | | | | | | | | | | | | | | | | | | | | | | | | | | | | | | | | | | | | | | | | | | | | | | | | | | | | | | | | | | | | | | | | | | | | | | | | | | | | | | | | | | | | | | | | | | | | | | | | | | | | | | | | | | | | | | | | | | | | | | | | | | | | | | | | | | | | | | | | | | | | | | | | | | | | | | | | | | | | | | | | | | | | | | | | | | | | | | | | | | | | | | | | | | | | | | | | | | | | | | | | | | | | | | | | | | | | | | | | |  | | | | | | | | | | | | | | | | |  | | | | | | | | | | | | |  | | | | | | | | | | | |  | | | | | | | | | | | | | | |  | | | | | | | | | | | | | | | | |  | | | | | | | | | | | | | | | |  | | | | | | | | | | | | |  | | | | | | | | | | | | | |  | | | | | | | | | | | | | | | | |  | | | | | | | | | | | | | | | | | |  | | | | | | | | | | | | | | | | | |  | | | | | | | | | | | | | | | |  | | | | | | | | | | | | | | | | | | |  | | | | | | | | | | | | | | | | | |  | | | |
|  | |  | | | | | | | | | | | | | | |  | | | | |  | | | | | | | | | | | | | | | | | | | | |  | | | | | | | |  | | | | | | | | | | | |  | | | | | | | | | | | | | | |  | | | | | | | | | | | | | |  | | | | | | | | | | | | | | |  | | | | | | | | | |  | | | | | | | | | | | | | | | | | | |  | | | | | | | | | | | | | | |  | | | | | | | | | | | | | | |  | | | | | | | | | | | | |  | | | | | | | | | | | | | | | | | | | | | | | | |  | | | | | | | | |  | | | | | | | | | | | | | |  | | | | | | | | | | | | | | | | | | | | | **29- Do you think transmission risk of malaria is more understood after the intervention ?** | | | | | | | | | | | | | | | | | | | | | | | | | | | | | | | | | | | | | | | | | | | | | | | | | | | | | | | | | | | | | | | | | | | | | | | | | | | | | | | | | | | | | | | | | | | | | | | | | | | | | | | | | | | | | | | | | | | | | | | | | | | | | | | | | | | | | | | | | | | | | | | | | | | | | | | | | | | | | | | | | | | | | | | | | | | | | | | | | | | | | | | | | | | | | | | | | | | | | | | | | | | | |  | | | | | | | | | | | | | | | |  | | | |
| **28- How is your utilization of mosquito nets compared to before the intervention ?** | | | | | | | | | | | | | | | | | | | | | | | | | | | | | | | | | | | | | | | | | | | | | | | | | | | | | | | | | | | | | | | | | | | | | | | | | | | | | | | | | | | | | | | | | | | | | | | | | | | | | | | | | | | | | | | | | | | | | | | | | | | | | | | | | | | | | | | | | | | | | | | | | | | | | | | | | | | | | | | | | | | | | | | | | | | | | | | | | | | | | | | | | | | | | | | | | | | | | | | | | | | | | | | | | | | | | | | | | | | | | | | | | | | | | | | | | | | | | | | | | | | | | | | | | | | | | | | | | | | | | | |  | | | | | | | | | | | | | | | | | | | | | | | | | | | | | | | | | | | | | | | | | | | | | | | | | | | | | | | | | | | | | | | | | | | | | | | | | | | | | | | | | | | | | | | | | | | | | | | | | | | | | | | | | | | | | | | | | | | | | | | | | | | | | | | | | | | | | | | | | | | | | | | | | | | | | | | | | | | | | | | | | | | | | | | | |  | | | | | | | | | | | | | | | | | | |  | | | | | | | | | | | | | | | | | |  | | | |
|  | | 1.More often | | | | | | | | | | | | | | | | | | | | | | | | | | | | | | | | | | | | | | | | | | | | | | | | |  | | | | | | | | | | | |  | | | | | | | | | | | | | | |  | | | | | | | | | | | | | |  | | | | | | | | | | | | | | |  | | | | | | | | | |  | | | | | | | | | | | | | | | | | | |  | | | | | | | | | | | | | | |  | | | | | | | | | | | | | | |  | | | | | | | | | | | | |  | | | | | | | | | | | | | | | | | | | | | | | | |  | | | | | | | | |  | | | | | | | | | | | | | |  | | | | | | | | | | | | | | | | | | | | |  | | | | | | | | | | | | | | | |  | | | | | | | | | | | | | 1- Yes | | | | | | | | | | | | | | | | | | | | | | | | | | |  | | | | | | | | | | | | | | | | |  | | | | | | | | | | | | | | | | |  | | | | | | | | | | | | |  | | | | | | | | | | | | | |  | | | | | | | | | | | | | | | |  | | | | | | | | | | | | | | | | | |  | | | | | | | | | | | | | | | | | | |  | | | | | | | | | | | | | | | |  | | | | | | | | | | | | | | | | | | |  | | | | | | | | | | | | | | | |  | | | |
|  | | 2.No change | | | | | | | | | | | | | | | | | | | | | | | | | | | | | | | | | | | | | | | | | | | | | | | | | | | | | | | | | | | | | | | | | | | | | | | | | | | |  | | | | | | | | | | | | | |  | | | | | | | | | | | | | | |  | | | | | | | | | |  | | | | | | | | | | | | | | | | | | |  | | | | | | | | | | | | | | |  | | | | | | | | | | | | | | |  | | | | | | | | | | | | |  | | | | | | | | | | | | | | | | | | | | | | | | |  | | | | | | | | |  | | | | | | | | | | | | | |  | | | | | | | | | | | | | | | | | | | | |  | | | | | | | | | | | | | | | |  | | | | | | | | | | | | | 2- No | | | | | | | | | | | | | | | | | | | | | | | | | | |  | | | | | | | | | | | | | | | | |  | | | | | | | | | | | | | | | | |  | | | | | | | | | | | | |  | | | | | | | | | | | | | |  | | | | | | | | | | | | | | | |  | | | | | | | | | | | | | | | | | |  | | | | | | | | | | | | | | | | | | |  | | | | | | | | | | | | | | | |  | | | | | | | | | | | | | | | | | | |  | | | | | | | | | | | | | | | |  | | | |
|  | | 3.Less often | | | | | | | | | | | | | | | | | | | | | | | | | | | | | | | | | | | | | | | | | | | | | | | | |  | | | | | | | | | | | |  | | | | | | | | | | | | | | |  | | | | | | | | | | | | | |  | | | | | | | | | | | | | | |  | | | | | | | | | |  | | | | | | | | | | | | | | | | | | |  | | | | | | | | | | | | | | |  | | | | | | | | | | | | | | |  | | | | | | | | | | | | |  | | | | | | | | | | | | | | | | | | | | | | | | |  | | | | | | | | |  | | | | | | | | | | | | | |  | | | | | | | | | | | | | | | | | | | | |  | | | | | | | | | | | | | | | |  | | | | | | | | | | | | | 3-No change | | | | | | | | | | | | | | | | | | | | | | | | | | | | | | | | | | | | | | | | | | | | | | | | | | | | | | | | | | | | | | | | | | | | | | | | | |  | | | | | | | | | | | | | |  | | | | | | | | | | | | | | | |  | | | | | | | | | | | | | | | | | |  | | | | | | | | | | | | | | | | | | |  | | | | | | | | | | | | | | | |  | | | | | | | | | | | | | | | | | | |  | | | | | | | | | | | | | | | |  | | | |
|  | |  | | | | | | | | | | | | | | |  | | | | |  | | | | | | | | | | | | | | | | | | | | |  | | | | | | | |  | | | | | | | | | | | |  | | | | | | | | | | | | | | |  | | | | | | | | | | | | | |  | | | | | | | | | | | | | | |  | | | | | | | | | |  | | | | | | | | | | | | | | | | | | |  | | | | | | | | | | | | | | |  | | | | | | | | | | | | | | |  | | | | | | | | | | | | |  | | | | | | | | | | | | | | | | | | | | | | | | |  | | | | | | | | |  | | | | | | | | | | | | | |  | | | | | | | | | | | | | | | | | | | | |  | | | | | | | | | | | | | | | |  | | | | | | | | | | | | |  | | | | | | | | | | | | |  | | | | | | | | | | | | | |  | | | | | | | | | | | | | | | | |  | | | | | | | | | | | | | | | | |  | | | | | | | | | | | | |  | | | | | | | | | | | | | |  | | | | | | | | | | | | | | | |  | | | | | | | | | | | | | | | | | |  | | | | | | | | | | | | | | | | | | |  | | | | | | | | | | | | | | | |  | | | | | | | | | | | | | | | | | | |  | | | | | | | | | | | | | | | |  | | | |
| **Open questions:** | | | | | | | | | | | | | | | | | | | | | | | | | | | | | | | | | | | | | | | | | | | | | | | | | | | | | | | | | | | | | | | | | | | | | | | | | | | | | |  | | | | | | | | | | | | | |  | | | | | | | | | | | | | | |  | | | | | | | | | |  | | | | | | | | | | | | | | | | | | |  | | | | | | | | | | | | | | |  | | | | | | | | | | | | | | |  | | | | | | | | | | | | |  | | | | | | | | | | | | | | | | | | | | | | | | |  | | | | | | | | |  | | | | | | | | | | | | | |  | | | | | | | | | | | | | | | | | | | | |  | | | | | | | | | | | | | | | |  | | | | | | | | | | | | |  | | | | | | | | | | | | |  | | | | | | | | | | | | | |  | | | | | | | | | | | | | | | | |  | | | | | | | | | | | | | | | | |  | | | | | | | | | | | | |  | | | | | | | | | | | | | |  | | | | | | | | | | | | | | | |  | | | | | | | | | | | | | | | | | |  | | | | | | | | | | | | | | | | | | |  | | | | | | | | | | | | | | | |  | | | | | | | | | | | | | | | | | | |  | | | | | | | | | | | | | | | |  | | | |
| **30-Suggestions for a similar larviciding campaign?** | | | | | | | | | | | | | | | | | | | | | | | | | | | | | | | | | | | | | | | | | | | | | | | | | | | | | | | | | | | | | | | | | | | | | | | | | | | | | | | | | | | | | | | | | | | | | | | | | | | | | | | | | | | | | | | | | | | | | | | | | | | | | | | | | | | | | | | | | | | | | | | | | | | | | | | | | | | | | | | | | | | | | | | | | | | | | | | | | | | | | | | | | | | | | | | | | | | | | | | | | | | | | | | | | | | | | | | | | | | | | | | | | | | | | | | | | | | | | | | | | | | | | |  | | | | | | | | | | | | | | | | |  | | | | | | | | | | | | |  | | | | | | | | | | | |  | | | | | | | | | | | | | | |  | | | | | | | | | | | | | | | | |  | | | | | | | | | | | | | | | |  | | | | | | | | | | | | |  | | | | | | | | | | | | | |  | | | | | | | | | | | | | | | | |  | | | | | | | | | | | | | | | | | |  | | | | | | | | | | | | | | | | | |  | | | | | | | | | | | | | | | |  | | | | | | | | | | | | | | | | | | |  | | | | | | | | | | | | | | | | | |  | | | |
|  | |  | | | | | | | | | | | | | | |  | | | | |  | | | | | | | | | | | | | | | | | | | | |  | | | | | | | |  | | | | | | | | | | | |  | | | | | | | | | | | | | | |  | | | | | | | | | | | | | |  | | | | | | | | | | | | | | |  | | | | | | | | | |  | | | | | | | | | | | | | | | | | | |  | | | | | | | | | | | | | | |  | | | | | | | | | | | | | | |  | | | | | | | | | | | | |  | | | | | | | | | | | | | | | | | | | | | | | | |  | | | | | | | | |  | | | | | | | | | | | | | |  | | | | | | | | | | | | | | | | | | | | |  | | | | | | | | | | | | | | | |  | | | | | | | | | | | | |  | | | | | | | | | | | | |  | | | | | | | | | | | | | |  | | | | | | | | | | | | | | | | |  | | | | | | | | | | | | | | | | |  | | | | | | | | | | | | |  | | | | | | | | | | | | | |  | | | | | | | | | | | | | | | |  | | | | | | | | | | | | | | | | | |  | | | | | | | | | | | | | | | | | | |  | | | | | | | | | | | | | | | |  | | | | | | | | | | | | | | | | | | |  | | | | | | | | | | | | | | | |  | | | |
|  | |  | | | | | | | | | | | | | | |  | | | | |  | | | | | | | | | | | | | | | | | | | | |  | | | | | | | |  | | | | | | | | | | | |  | | | | | | | | | | | | | | |  | | | | | | | | | | | | | |  | | | | | | | | | | | | | | |  | | | | | | | | | |  | | | | | | | | | | | | | | | | | | |  | | | | | | | | | | | | | | |  | | | | | | | | | | | | | | |  | | | | | | | | | | | | |  | | | | | | | | | | | | | | | | | | | | | | | | |  | | | | | | | | |  | | | | | | | | | | | | | |  | | | | | | | | | | | | | | | | | | | | |  | | | | | | | | | | | | | | | |  | | | | | | | | | | | | |  | | | | | | | | | | | | |  | | | | | | | | | | | | | |  | | | | | | | | | | | | | | | | |  | | | | | | | | | | | | | | | | |  | | | | | | | | | | | | |  | | | | | | | | | | | | | |  | | | | | | | | | | | | | | | |  | | | | | | | | | | | | | | | | | |  | | | | | | | | | | | | | | | | | | |  | | | | | | | | | | | | | | | |  | | | | | | | | | | | | | | | | | | |  | | | | | | | | | | | | | | | |  | | | |
|  | |  | | | | | | | | | | | | | | |  | | | | |  | | | | | | | | | | | | | | | | | | | | |  | | | | | | | |  | | | | | | | | | | | |  | | | | | | | | | | | | | | |  | | | | | | | | | | | | | |  | | | | | | | | | | | | | | |  | | | | | | | | | |  | | | | | | | | | | | | | | | | | | |  | | | | | | | | | | | | | | |  | | | | | | | | | | | | | | |  | | | | | | | | | | | | |  | | | | | | | | | | | | | | | | | | | | | | | | |  | | | | | | | | |  | | | | | | | | | | | | | |  | | | | | | | | | | | | | | | | | | | | |  | | | | | | | | | | | | | | | |  | | | | | | | | | | | | |  | | | | | | | | | | | | |  | | | | | | | | | | | | | |  | | | | | | | | | | | | | | | | |  | | | | | | | | | | | | | | | | |  | | | | | | | | | | | | |  | | | | | | | | | | | | | |  | | | | | | | | | | | | | | | |  | | | | | | | | | | | | | | | | | |  | | | | | | | | | | | | | | | | | | |  | | | | | | | | | | | | | | | |  | | | | | | | | | | | | | | | | | | |  | | | | | | | | | | | | | | | |  | | | |
|  | |  | | | | | | | | | | | | | | |  | | | | |  | | | | | | | | | | | | | | | | | | | | |  | | | | | | | |  | | | | | | | | | | | |  | | | | | | | | | | | | | | |  | | | | | | | | | | | | | |  | | | | | | | | | | | | | | |  | | | | | | | | | |  | | | | | | | | | | | | | | | | | | |  | | | | | | | | | | | | | | |  | | | | | | | | | | | | | | |  | | | | | | | | | | | | |  | | | | | | | | | | | | | | | | | | | | | | | | |  | | | | | | | | |  | | | | | | | | | | | | | |  | | | | | | | | | | | | | | | | | | | | |  | | | | | | | | | | | | | | | |  | | | | | | | | | | | | |  | | | | | | | | | | | | |  | | | | | | | | | | | | | |  | | | | | | | | | | | | | | | | |  | | | | | | | | | | | | | | | | |  | | | | | | | | | | | | |  | | | | | | | | | | | | | |  | | | | | | | | | | | | | | | |  | | | | | | | | | | | | | | | | | |  | | | | | | | | | | | | | | | | | | |  | | | | | | | | | | | | | | | |  | | | | | | | | | | | | | | | | | | |  | | | | | | | | | | | | | | | |  | | | |
|  | |  | | | | | | | | | | | | | | |  | | | | |  | | | | | | | | | | | | | | | | | | | | |  | | | | | | | |  | | | | | | | | | | | |  | | | | | | | | | | | | | | |  | | | | | | | | | | | | | |  | | | | | | | | | | | | | | |  | | | | | | | | | |  | | | | | | | | | | | | | | | | | | |  | | | | | | | | | | | | | | |  | | | | | | | | | | | | | | |  | | | | | | | | | | | | |  | | | | | | | | | | | | | | | | | | | | | | | | |  | | | | | | | | |  | | | | | | | | | | | | | |  | | | | | | | | | | | | | | | | | | | | |  | | | | | | | | | | | | | | | |  | | | | | | | | | | | | |  | | | | | | | | | | | | |  | | | | | | | | | | | | | |  | | | | | | | | | | | | | | | | |  | | | | | | | | | | | | | | | | |  | | | | | | | | | | | | |  | | | | | | | | | | | | | |  | | | | | | | | | | | | | | | |  | | | | | | | | | | | | | | | | | |  | | | | | | | | | | | | | | | | | | |  | | | | | | | | | | | | | | | |  | | | | | | | | | | | | | | | | | | |  | | | | | | | | | | | | | | | |  | | | |
